# Evolutionary multiplayer games on graphs with edge diversity

**DOI:** 10.1371/journal.pcbi.1006947

**Published:** 2019-04-01

**Authors:** Qi Su, Lei Zhou, Long Wang

**Affiliations:** 1 Center for Systems and Control, College of Engineering, Peking University, Beijing, China; 2 Center for Polymer Studies, Department of Physics, Boston University, Boston, Massachusetts, United States of America; University of Sussex, UNITED STATES

## Abstract

Evolutionary game dynamics in structured populations has been extensively explored in past decades. However, most previous studies assume that payoffs of individuals are fully determined by the strategic behaviors of interacting parties, and social ties between them only serve as the indicator of the existence of interactions. This assumption neglects important information carried by inter-personal social ties such as genetic similarity, geographic proximity, and social closeness, which may crucially affect the outcome of interactions. To model these situations, we present a framework of evolutionary multiplayer games on graphs with edge diversity, where different types of edges describe diverse social ties. Strategic behaviors together with social ties determine the resulting payoffs of interactants. Under weak selection, we provide a general formula to predict the success of one behavior over the other. We apply this formula to various examples which cannot be dealt with using previous models, including the division of labor and relationship- or edge-dependent games. We find that labor division can promote collective cooperation markedly. The evolutionary process based on relationship-dependent games can be approximated by interactions under a transformed and unified game. Our work stresses the importance of social ties and provides effective methods to reduce the calculating complexity in analyzing the evolution of realistic systems.

## Author summary

The outcome of an interaction often relies on not only interactants’ strategic behaviors but also genetic and physical relationships between interactants, such as genetic similarity and geographic proximity. Thus when encountering different opponents who use the same strategy, an individual may derive different payoffs. Social ties, acting as carriers of such information, are crucial to biological interactions. However, most prior studies simplify social ties as binary states (i.e., either present or absent) and ignore the information carried. Here we study evolutionary multiplayer games on graphs and introduce different types of edges to describe diverse social ties. We derive a simple rule to predict when a strategic behavior is more successful than the other. Based on this rule, we find that the labor division in eusocial insects could promote prosocial behavior. In addition, when payoff structures in different interactions are relationship-dependent, the condition for the success of one behavior can be obtained by studying interactions described by a unified payoff structure. Our work not only extends established results on the evolution of cooperation on graphs, but also shows the possibility to simplify complex and diverse interactions in real-world systems as simple and unified interactions in theoretical calculations.

## Introduction

Understanding the emergence and persistence of cooperation in the population of egoists is an enduring challenge that has inspired a myriad of studies from biology to sociology [1]. Evolutionary game theory has been widely employed to investigate this cooperation conundrum at different levels of living systems [2]. Typically, social dilemmas are depicted by two-player two-strategy games where each player can choose either to cooperate or to defect [3]. In these games, mutual cooperation brings each player a reward *R* while mutual defection a punishment *P*; when a cooperator encounters a defector, the cooperator obtains a sucker’s payoff *S* and the defector gets the temptation *T*. Different rankings of payoff entries *R*, *S*, *T*, *P* represent different social dilemmas [3]. Despite the simplicity of this representation, in the real world, many interactions occur beyond the dyadic scenarios and often involve more than two individuals. For examples, in a S. cerevisiae population, a cooperative yeast produces an enzyme to hydrolyze sucrose into monosaccharides while the most of them diffuse away and are exploited by nearby yeasts [4] (see Ref. [5, 6] for more examples in microbes and Ref. [7, 8] in human societies). Interactions in these examples are better modeled by multiplayer games [9]. Generally, multiplayer games cannot be represented by a collection of two-player games [10] whereas the latter can always be regarded as the simplest case of the former [9], making the study of multiplayer games of great importance for the evolution of cooperation [11, 12]. One particular example is the threshold public goods game [13]. It captures the strategic interactions of individuals when the provision of public goods needs a threshold surpassed. Such a threshold can be a minimum amount of funding for building national defense, a minimum height of a dam for securing the public safety, etc [8]. In this game, each individual has two options—to contribute an amount of investment to the goods pool or not to contribute. The benefit is provided only when the total investment exceeds a threshold [13].

Recent advance in exploring interaction patterns of living agents shows that populations often exhibit structural characteristics, which expands our research interests in evolutionary dynamics from traditional well-mixed to structured populations [14–25]. Graphs serve as a good tool to model such a system, where vertices of graphs represent individuals and edges specify one’s interaction and dispersal neighborhoods. In the case of weak selection where individuals’ payoffs obtained from games slightly affect their fitness or reproductive rates, evolutionary outcomes on graphs, especially the conditions for one strategy to be favored over the other, can be tackled analytically. For example, Tarnita *et al*. derive a simple condition to predict the evolutionary outcome for two-player two-strategy games [26]. This condition relies on all the payoff entries *R*, *S*, *T*, *P* and one “structure coefficient”. As shown in their work, the structure coefficient summarizes all the effects of a population structure on the condition for the success of strategies and it is independent of payoff entries. Due to the generality of the above results, calculating structure coefficients provides a convenient way to quantify the effect of population structures on the evolutionary outcome [10, 23, 27–31]. Nonetheless, the closed-form expressions of the structure coefficients are often hard to calculate under multiplayer games, even in the simplest well-mixed populations [9]. This becomes even more challenging when the population structure is taken into account. Even so, there are still a few seminal work about evolutionary multiplayer games on graphs [10, 31–35]. For example, Peña *et al*. derive the structure coefficients for evolutionary multiplayer games on finite ring graphs and infinite regular graphs [33]. Based on competition between territorial animals, Broom *et al*. develop a new modelling framework to investigate collective interactions, which is capable and flexible to compare and analyze various spatial structures [34]. McAvoy *et al*. study when a multiplayer game can be broken down into a sequence of interactions with fewer individuals and show that a simple population structure can greatly complicate the reduction [10].

Prior studies about games on graphs usually assume that social ties between individuals only indicate the presence of interactions [10, 20, 31–37]. The other relevant information associated with social ties, such as the genetic and physical relationships between interactants, is often ignored. In such cases, individuals’ strategic behaviors are the only determinant of the outcome of an interaction. Typically, in two-player interactions, if two distinct individuals take the same strategy, their common opponent obtains the same payoff when encountering each of them separately [14, 15]. When engaging in group interactions, one’s payoff relies on the number of opposing cooperators but is independent of which one is the cooperator [32, 33]. Indeed, this assumption significantly reduces the calculation complexity and thus makes it possible for many well-known results [38, 39]. However, recent studies show that overlooking the information of social ties could make theoretical predictions deviate greatly from empirical observations [40–43]. For example, people possess strong and weak social ties, such as intimate interpersonal relationships with relatives and tenuous relationships with acquaintance [43, 44]; failing to account for the tie strengths leads to a globally accelerated information diffusion and a remarkably distinct diffusion direction from that in actual networks [41, 42]. In well-mixed populations, when distinct frequencies of interactions between pairs are considered, altruistic traits can flourish whereas neglecting such information on social ties leads to the extinction of altruism [23]. Here, the second example clearly conveys that the information associated with social ties can affect the evolution of a certain behavioral trait (strategy) in a nontrivial way. Besides, we offer two other representative cases. In the example of the division of labor in colonies of eusocial insects and human societies, the production of collective benefits needs different individuals to cooperatively perform different subtasks [45–48]. When many individuals assigned one subtask cooperate, cooperation from an individual assigned another subtask is more crucial to the colony productivity than cooperation from individuals assigned the same subtask. The other situation is that the payoff structure of an interaction may be relationship-dependent [49]. It means that an individual may concurrently play various types of games with its neighbors, depending on the social tie they are connected with [50, 51]. For instance, individuals can play coordinations games (or even harmony games) with its friends and prisoner’s dilemma with strangers.

To better understand the role of social ties in the evolution of strategic behaviors, we present a comprehensive framework of evolutionary multiplayer games on graphs with edge diversity. Each type of edges describes one kind of relationship between two connected individuals, such as having the same or different task skills [46–48, 52], owning close or distinct consanguinity or geographical distance and so on. We investigate both finite and infinite regular graphs with *n* types of edges. We provide a simple condition to predict when natural selection favors one strategic behavior over the other. The condition is validated by Monte Carlo simulations. Applying it to the case of division of labor where cooperation from individuals performing different subtasks is required for producing benefits (see the example of army ants retrieving prey items [46]), we find labor division significantly lowers the barrier to establish cooperative society. Then we explore the scenario where each individual simultaneously participates in many multiplayer games and these games can differ in payoff entries or metaphors. We find evolutionary dynamics for such diverse interactions can be approximated by an evolutionary process with a unified payoff structure. This result provides us insights into simplifying complex and diverse interactions in real-world systems as simple and unified interactions in theoretical calculations. Our work also covers the evolutionary games on weighted graphs (see the example of bacterium Escherichia coli [53]). Intriguingly, in our framework, strong edges do not act as a promoter of cooperation.

## Models

**Fig 1:**
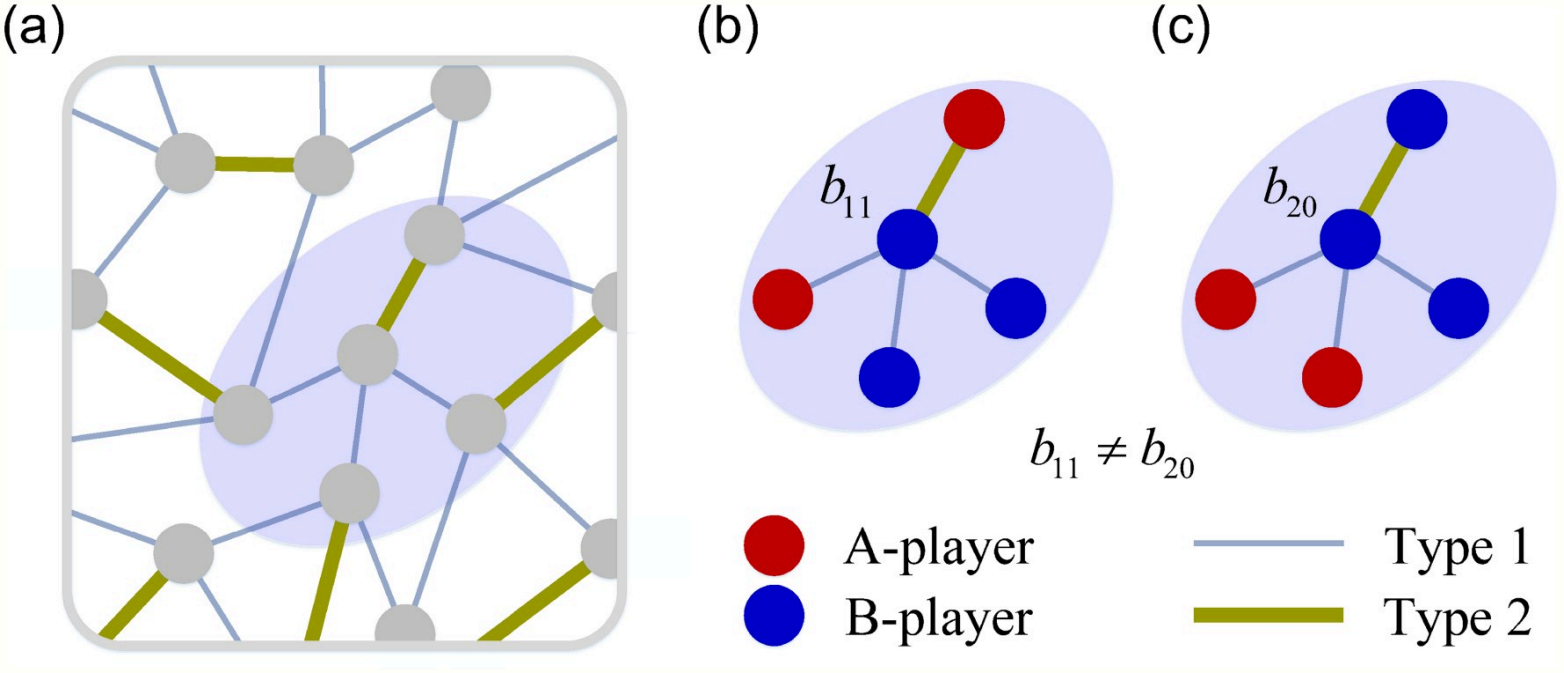
Illustration of evolutionary multiplayer games on graphs with edge diversity. (a) The population structure is depicted by a regular graph, where each node has *k* = 4 neighbors connected by two types of edges. In detail, in a typical neighborhood (the highlighted zone), the focal (i.e., centered) node has *g*_1_ = 3 edges of type 1 (thin blue lines) and *g*_2_ = 1 edge of type 2 (thick olive line). On the graph, each node is occupied by an individual. The focal individual’s payoff is determined by the state of its neighborhood, including its own strategy, all its neighbors’ strategies and the types of edges. This is different from traditional models about multiplayer games on graphs where edges are not distinguished and individuals’ payoffs are solely determined by strategies [22, 33]. We illustrate this difference by two concrete examples in subgraph (b) and (c). (b) Interacting with an A-player (red circle) and two B-players (blue circle) linked by edges of type 1, and an A-player linked by an edge of type 2, the focal B-player gains a payoff *b*_11_. (c) Interacting with two A-players and a B-player linked by edges of type 1, and a B-player linked by an edge of type 2, the focal B-player obtains a payoff *b*_20_. In our model, *b*_11_ in general differs from *b*_20_, which is in stark contrast with the case where no edge diversity is present. In the latter case, *b*_11_ = *b*_20_ since the total number of neighboring A-players is the same.

Here we briefly introduce the model of evolutionary multiplayer games on graphs with edge diversity. We first consider the stochastic evolutionary dynamics on a graph-structured population with a finite size *N* and later investigate the dynamics in infinite populations. Each individual occupies a node of a random regular graph with degree *k*. Note that this graph is determined randomly and fixed during the process of evolution. On the graph, each node is linked to *k* other nodes and each edge is assigned an edge type selected from *n* types (1 ≤ *n* ≤ *k*). Among the *k* edges connected to a node, the number of type *i* edges is *g*_*i*_, which means $\sum_{i=1}^{n}g_{i}=k$. During the evolution, in each generation, every individual obtains a payoff by interacting with *k* adjacent individuals in a single game, analogous to the setting of spatial multiplayer game in prior studies [33, 54]. In the game, each individual chooses either strategy A or strategy B. We use (*s*_1_, *s*_2_, ⋯, *s*_*n*_) denote a neighborhood state: among *g*_*i*_ neighbors connected by edges of type *i*, the number of individuals using strategy A is *s*_*i*_ while the number of those using strategy B is *g*_*i*_ − *s*_*i*_. In such a neighborhood, a focal A-player gets a payoff $a_{s_{1}s_{2}\cdots s_{n}}$ while a focal B-player gets a payoff $b_{s_{1}s_{2}\cdots s_{n}}$. Fig 1 illustrates an example of the spatial structure and Table 1 presents the payoff structure for *n* = 2. Our model can recover the traditional setting by taking *n* = 1.

**Table 1:** Payoffs for A- and B-players in multiplayer games with two types of interaction partners.

Opposing A-players (Type 1, Type 2)	(0, 0)	(0, 1)	(1, 0)	⋯	(*s*_1_, *s*_2_)	⋯	(*g*_1_ − 1, *g*_2_)	(*g*_1_, *g*_2_ − 1)	(*g*_1_, *g*_2_)
Payoff to A	*a*_00_	*a*_01_	*a*_10_	⋯	$a_{s_{1}s_{2}}$	⋯	$a_{\left(g_{1}-1\right)g_{2}}$	$a_{g_{1}\left(g_{2}-1\right)}$	$a_{g_{1}g_{2}}$
Payoff to B	*b*_00_	*b*_01_	*b*_10_	⋯	$b_{s_{1}s_{2}}$	⋯	$b_{\left(g_{1}-1\right)g_{2}}$	$b_{g_{1}\left(g_{2}-1\right)}$	$b_{g_{1}g_{2}}$

(*s*_1_, *s*_2_) denotes the neighborhood state: among *g*_1_ neighbors connected by edges of type 1, the number of neighbors using strategy A is *s*_1_ and the number of those using strategy B is *g*_1_ − *s*_1_; among *g*_2_ neighbors connected by edges of type 2, the number of neighbors using strategy A is *s*_2_ and the number of those using strategy B is *g*_2_ − *s*_2_. Within an neighborhood of (*s*_1_, *s*_2_), a focal A-player derives payoff $a_{s_{1}s_{2}}$ and a focal B-player gains payoff $b_{s_{1}s_{2}}$.

After the interaction, individual *i*’s payoff *π*_*i*_ is transformed to its reproductive rate or fitness by *F*_*i*_ = 1 − *ω* + *ωπ*_*i*_, where *ω* represents the intensity of selection, i.e., the extent to which the payoff from games influences the reproductive success. Here we consider the weak selection (*ω* ≪ 1). The population evolves according to the death-birth rule [38]. This update rule can be interpreted in the context of genetic evolution or cultural evolution [16, 23]. In the genetic context, a random individual such as *i* is selected to die. After that, one of *i*’s neighbors is chosen to reproduce an offspring with probability proportional to its reproductive rate. Then this offspring occupies the vacant site. When the death-birth rule is interpreted as a genetic process, different evolutionary outcomes may occur, depending on the meaning of the edge type. For instance, if the edge type represents genetic similarity, during the evolution, the edge type may be changed due to the gene replication and dispersal. But if the edge type represents geographic proximity, the above genetic process does not change the edge types. To avoid the contingency of evolutionary outcomes on the specific meaning of the edge types, in this paper, we thus interpret the the death-birth rule as a kind of behavior imitation in the cultural evolution. That is, a random individual *i* resolves to update its strategy, and it adopts neighbor *j*’s strategy proportionally to *j*’s fitness, i.e., with probability $F_{j}/\sum_{l\in \Omega_{i}}F_{l}$, where Ω_*i*_ is the set of *i*’s neighbors. With this interpretation, for all possible meanings of edge types we considered in this model, the process of behavior imitation does not change the edge types.

## Results

### A general condition to predict the success of one strategic behavior

In finite populations, the fixation probability is a well-established measure to quantify the evolutionary success of different traits or strategies [55]. The fixation probability *ρ*_A_ denotes the probability that a single A-player starting in a random position propagates and takes over the whole population of B-players. Analogously, *ρ*_B_ is the probability that a single B-player starting in a random position propagates and takes over the whole population of A-players. Natural selection favors strategy A over B if

$$\begin{array}{c} \rho_{\text{A}}>\rho_{\text{B}}. \end{array}$$

Using weak selection, in large random regular graphs with *n* edge types (*k* ≥ 3 and 1 ≤ *g*_*i*_ ≤ *k*), we obtain the condition under which A-players are selected over B-players (see S1 File, Section 1), given by

$$\begin{array}{c} \sum_{s_{1}=0}^{g_{1}}\sum_{s_{2}=0}^{g_{2}}\cdots \sum_{s_{n}=0}^{g_{n}}\sigma_{s_{1}s_{2}\cdots s_{n}}(a_{s_{1}s_{2}\cdots s_{n}}-b_{\left(g_{1}-s_{1}\right)\left(g_{2}-s_{2}\right)\cdots \left(g_{n}-s_{n}\right)})>0, \end{array}$$ (1)

where $\sigma_{s_{1}s_{2}\cdots s_{n}}$ (0 ≤ *s*_1_ ≤ *g*_1_, 0 ≤ *s*_2_ ≤ *g*_2_, ⋯, 0 ≤ *s*_*n*_ ≤ *g*_*n*_) is the structure coefficient that relies on population structures and update rules but is independent of payoff values $a_{s_{1}s_{2}\cdots s_{n}}$ and $b_{s_{1}s_{2}\cdots s_{n}}$. There are totally $\Pi_{i=1}^{n}\left(g_{i}+1\right)$ structure coefficients for Eq (1). All structure coefficients here are positive and we can eliminate an extra structure coefficient through dividing the sigma rule [see Eq (1)] by any one of them. $\sigma_{s_{1}s_{2}\cdots s_{n}}$ can be approximated by

$$\begin{array}{cc} \sigma_{s_{1}s_{2}\cdots s_{n}}= & \frac{{\left(k-2\right)}^{\left(k-\sum_{j=1}^{n}s_{j}\right)}}{k^{2}\left(k+1\right)\left(k+2\right)}\frac{\Pi_{j=1}^{n}(\begin{array}{c} g_{j}\\ s_{j} \end{array})}{\left(\begin{array}{c} k\\ \sum_{j=1}^{n}s_{j} \end{array}\right)}\\ & \sum_{l=0}^{k}\left(k-l\right)\{[2k+(k-2)l]\Psi (k,\sum_{j=1}^{n}s_{j},l)+[k^{2}-\left(k-2\right)l]\Phi (k,\sum_{j=1}^{n}s_{j},l)\}, \end{array}$$

where

$$\begin{array}{l} \Psi (k,i,l)=\left(\begin{array}{c} l\\ k-1-i \end{array}\right)\frac{1}{(k-2){\left(k-1\right)}^{l}}+\left(\begin{array}{c} k-1-l\\ k-i \end{array}\right)\frac{1}{{\left(k-1\right)}^{k-1-l}},\\ \Phi (k,i,l)=\left(\begin{array}{c} l\\ k-i \end{array}\right)\frac{1}{{\left(k-1\right)}^{l}}+\left(\begin{array}{c} k-1-l\\ k-1-i \end{array}\right)\frac{1}{(k-2){\left(k-1\right)}^{k-1-l}}. \end{array}$$

$a_{s_{1}s_{2}\cdots s_{n}}-b_{\left(g_{1}-s_{1}\right)\left(g_{2}-s_{2}\right)\cdots \left(g_{n}-s_{n}\right)}$ in Eq (1) indicates the “gains from flipping” [33, 35], the change in payoffs for a focal A-player who interacts with *s*_*i*_ A-players of type *i* (1 ≤ *i* ≤ *n*) in a group when all individuals change their strategies (from strategy A to strategy B or B to A) simultaneously. Considering $\sum_{s_{1}=0}^{g_{1}}\sum_{s_{2}=0}^{g_{2}}\cdots \sum_{s_{n}=0}^{g_{n}}\sigma_{s_{1}s_{2}\cdots s_{n}}=1$, $\sigma_{s_{1}s_{2}\cdots s_{n}}$ can be viewed as a probability corresponding to term $a_{s_{1}s_{2}\cdots s_{n}}-b_{\left(g_{1}-s_{1}\right)\left(g_{2}-s_{2}\right)\cdots \left(g_{n}-s_{n}\right)}$. Eq (1) thus indicates that strategy A is favored over B if the expected gain in payoffs from flipping is positive. When *n* = 1, our analytical prediction is in line with a previous study about evolutionary multiplayer games on graphs [33] (see S1 File, Section 2). To understand the structure coefficient for the case with *n* > 1, we set the sum of the number of opposing A-players to be *S*, i.e., $\sum_{j=1}^{n}s_{j}=S$. We find that $\sigma_{s_{1}s_{2}\cdots s_{n}}$ is the product of the structure coefficient corresponding to *n* = 1 (denoted *σ*_*S*_) and an additional term $\prod_{j=1}^{n}\left(\begin{array}{l} g_{j}\\ s_{j} \end{array}\right)/\left(\begin{array}{c} k\\ \sum_{j=1}^{n}s_{j} \end{array}\right)$. This term represents the probability of the configuration *s*_1_ *s*_2_⋯*s*_*n*_ to occur under a given *S*. Intuitively, with edge diversity, we distinguish A-players in the neighborhood by their types. For a given number of A-players *S*, the probability of a specific configuration (*s*_*i*_ A-players within *g*_*i*_ individuals of type *i*) indeed follows the multivariate hypergeometric distribution $\prod_{j=1}^{n}\left(\begin{array}{l} g_{j}\\ s_{j} \end{array}\right)/\left(\begin{array}{c} k\\ S \end{array}\right)$. Our result shows that the structure coefficient associated with a specific configuration for diverse edges is simply a product of the probability for this configuration to occur and the corresponding structure coefficient without distinguishing edges.

Infinite populations usually serve as a baseline model to investigate the evolutionary dynamics of a system. Therefore we conduct a consistent investigation in infinite populations. The evolutionary dynamics of multiplayer games on graphs with edge diversity can be described in terms of replicator equation [56] (see S1 File, Section 4), given by

$$\begin{array}{c} \dot{x}=\frac{\omega (k-2)x(1-x)}{k^{2}}f\left(x\right), \end{array}$$ (2)

where

$$f(x)=\sum_{s_{1}=0}^{g_{1}}\sum_{s_{2}=0}^{g_{2}}\cdots \sum_{s_{n}=0}^{g_{n}}\left[\prod_{j=1}^{n}\left(\begin{array}{c} g_{j}\\ s_{j} \end{array}\right)x^{s_{j}}{\left(1-x\right)}^{g_{j}-s_{j}}\right]\left(\Lambda_{a}-\Lambda_{b}\right),$$

$$\begin{array}{cc} \Lambda_{a}= & \sum_{r_{1}=0}^{g_{1}-s_{1}}\sum_{r_{2}=0}^{g_{2}-s_{2}}\cdots \sum_{r_{n}=0}^{g_{n}-s_{n}}[\prod_{j=1}^{n}\left(\begin{array}{c} g_{j}-s_{j}\\ r_{j} \end{array}\right)z^{r_{j}}{\left(1-z\right)}^{g_{j}-s_{j}-r_{j}}]\\ & \sum_{j=1}^{n}[(s_{j}+r_{j})a_{\left(s_{1}+r_{1}\right)\left(s_{2}+r_{2}\right)\cdots \left(s_{n}+r_{n}\right)}\\ & \;\;+(zs_{j}+\frac{r_{j}}{z})a_{\left(s_{1}+r_{1}-\delta_{1j}\right)\left(s_{2}+r_{2}-\delta_{2j}\right)\cdots \left(s_{n}+r_{n}-\delta_{nj}\right)}], \end{array}$$

$$\begin{array}{cc} \Lambda_{b}= & \sum_{r_{1}=0}^{s_{1}}\sum_{r_{2}=0}^{s_{2}}\cdots \sum_{r_{n}=0}^{s_{n}}[\prod_{j=1}^{n}\left(\begin{array}{c} s_{j}\\ r_{j} \end{array}\right)z^{r_{j}}{\left(1-z\right)}^{s_{j}-r_{j}}]\\ & \sum_{j=1}^{n}[(g_{j}-s_{j}+r_{j})b_{\left(s_{1}-r_{1}\right)\left(s_{2}-r_{2}\right)\cdots \left(s_{n}-r_{n}\right)}\\ & \;\;+(z\left(g_{j}-s_{j}\right)+\frac{r_{j}}{z})b_{\left(s_{1}-r_{1}+\delta_{1j}\right)\left(s_{2}-r_{2}+\delta_{2j}\right)\cdots \left(s_{n}-r_{n}+\delta_{nj}\right)}], \end{array}$$

*z* = 1/(*k* − 1). *δ*_*ij*_ equals to 1 if *j* = *i* and 0 otherwise. This seemingly complicated Eq (2) could be greatly simplified when applied to specific examples, from pairwise games [56] to traditional multiplayer games such as volunteer’s dilemmas [57], multiplayer stag-hunt game [13], and multiplayer snowdrift game [58].

### Applications

When strategy A represents cooperation and B defection, Eq (1) can effectively predict the success of cooperation over defection. In the following, we apply Eqs (1) and (2) to four representative examples and organize them as follows. Example 1 describes two-player games. Different from previous studies, here each type of edges are endowed with an independent payoff matrix. Example 2 presents the scenario where every individual participates in different multiplayer games concurrently. Example 3 discusses the evolutionary multiplayer games on weighted graphs, where edge weights represent interaction rates. In example 4, we study the prevailing collective activity in social insects and human societies—division of labor.

#### Example 1. Evolutionary two-player games on graphs with edge diversity

In each generation, an individual plays a two-player game with each neighbor to derive a payoff and accumulates its payoffs from all interactions. We endow each type of edges with an independent payoff matrix. The payoff matrix for interactions occurring in edges of type *i* is

$$\begin{array}{ll} & \begin{array}{cc} \text{A} & \text{B} \end{array}\\ \begin{array}{c} \text{A}\\ \text{B} \end{array} & \left(\begin{array}{ll} \alpha_{i} & \beta_{i}\\ \gamma_{i} & \theta_{i} \end{array}\right) \end{array},$$

where each value corresponds to the payoff assigned to the individual adopting a strategy in the row against its partner taking a strategy in the column. Transforming the payoff to multiplayer interactions through $a_{s_{1}s_{2}\cdots s_{n}}=\sum_{i=1}^{n}[s_{i}\alpha_{i}+\left(g_{i}-s_{i}\right)\beta_{i}]$ and $b_{s_{1}s_{2}\cdots s_{n}}=\sum_{i=1}^{n}[s_{i}\gamma_{i}+\left(g_{i}-s_{i}\right)\theta_{i}]$, we have the sigma rule from Eq (1)

$$\begin{array}{c} \sum_{i=1}^{n}{\bar{s}}_{i}\alpha_{i}+\sum_{i=1}^{n}(g_{i}-{\bar{s}}_{i})\beta_{i}-\sum_{i=1}^{n}(g_{i}-{\bar{s}}_{i})\gamma_{i}-\sum_{i=1}^{n}{\bar{s}}_{i}\theta_{i}>0, \end{array}$$

where

$$\begin{array}{c} {\bar{s}}_{i}=\sum_{s_{1}=0}^{g_{1}}\sum_{s_{2}=0}^{g_{2}}\cdots \sum_{s_{n}=0}^{g_{n}}\sigma_{s_{1}s_{2}\cdots s_{n}}s_{i}. \end{array}$$

Applying (see S1 File, Section 3)

$$\begin{array}{c} \sum_{s_{1}=0}^{g_{1}}\sum_{s_{2}=0}^{g_{2}}\cdots \sum_{s_{n}=0}^{g_{n}}\sigma_{s_{1}s_{2}\cdots s_{n}}s_{i}=\frac{g_{i}\left(k+1\right)}{2k}, \end{array}$$

we have the sigma rule for evolutionary two-player games on graphs with edge diversity

$$\begin{array}{c} \sum_{i=1}^{n}[g_{i}\left(k+1\right)\alpha_{i}+g_{i}\left(k-1\right)\beta_{i}]>\sum_{i=1}^{n}[g_{i}\left(k-1\right)\gamma_{i}+g_{i}\left(k+1\right)\theta_{i}]. \end{array}$$

Since $\sum_{i=1}^{n}g_{i}=k$, dividing both sides of the above condition by *k*(*k* − 1), we obtain the simplified condition

$$\begin{array}{c} \frac{k+1}{k-1}\bar{\alpha }+\bar{\beta }>\bar{\gamma }+\frac{k+1}{k-1}\bar{\theta }, \end{array}$$

where $\bar{\alpha }=\left(1/k\right)\sum_{i=1}^{n}g_{i}\alpha_{i}$, $\bar{\beta }=\left(1/k\right)\sum_{i=1}^{n}g_{i}\beta_{i}$, $\bar{\gamma }=\left(1/k\right)\sum_{i=1}^{n}g_{i}\gamma_{i}$, and $\bar{\theta }=\left(1/k\right)\sum_{i=1}^{n}g_{i}\theta_{i}$. The above condition suggests that for pairwise games contingent on the edges, it suffices to study a unified game with its payoff entries averaged over all the games. Note that for the unified game, the associated structure coefficient is (*k* + 1)/(*k* − 1), which coincides with that with *n* = 1 [26, 38]. Moreover, if all the games are in the form of donations games, i.e., $\alpha_{i}=B_{i}-C_{i}$, $\beta_{i}=-C_{i}$, $\gamma_{i}=B_{i}$, and *θ*_*i*_ = 0, the condition for natural selection favoring cooperation over defection is

$$\begin{array}{c} \frac{\bar{B}}{\bar{C}}>k, \end{array}$$ (3)

where $\bar{B}=\left(1/k\right)\sum_{i=1}^{n}g_{i}B_{i}$ and $\bar{C}=\left(1/k\right)\sum_{i=1}^{n}g_{i}C_{i}$. This equation thus extends a well-known B/C>k rule (B and C are respectively the benefit and cost of the donative behavior) [38] to a general $\bar{B}/\bar{C}>k$ rule where $\bar{C}$ means the average cost for cooperative behavior on all possible types of edges and $\bar{B}$ is the average benefit [59].

#### Example 2. Diverse multiplayer games

Next we consider the case that each individual is involved in many multiplayer games concurrently and these games can differ in payoff structures (game metaphors and payoff values). To model this situation, we let individuals linked by the same type of edges form a group to play a multiplayer game and thus each focal individual participates in *n* multiplayer games. We assume that any two games are independent and each individual accumulates its payoffs from all interactions, i.e.,

$$\begin{array}{cc} & a_{s_{1}s_{2}\cdots s_{n}}=a_{s_{1}}^{\left(1\right)}+a_{s_{2}}^{\left(2\right)}+\cdots +a_{s_{n}}^{\left(n\right)},\\ & b_{s_{1}s_{2}\cdots s_{n}}=b_{s_{1}}^{\left(1\right)}+b_{s_{2}}^{\left(2\right)}+\cdots +b_{s_{n}}^{\left(n\right)}, \end{array}$$

where $a_{s_{i}}^{\left(i\right)}$ ($b_{s_{i}}^{\left(i\right)}$) represents the payoff assigned to an A-player (a B-player) in the interaction with individuals linked by edges of type *i* when there are *s*_*i*_ opposing A-players. If *g*_1_ = *g*_2_ = ⋯ = *g*_*n*_ = *g*, Eq (1) can be simplified as (see S1 File, Section 3)

$$\begin{array}{c} \sum_{s=0}^{g}{\tilde{\sigma }}_{s}(\sum_{j=1}^{n}a_{s}^{\left(j\right)}-\sum_{j=1}^{n}b_{g-s}^{\left(j\right)})>0, \end{array}$$ (4)

where ${\tilde{\sigma }}_{s}=\sum_{s_{2}=0}^{g_{2}}\sum_{s_{3}=0}^{g_{3}}\cdots \sum_{s_{n}=0}^{g_{n}}\sigma_{ss_{2}\cdots s_{n}}$. For such a system, we just need *g* + 1 structure coefficients to describe the effects of population structures on the evolution of two traits. If designating

$$\begin{array}{cc} & {\bar{a}}_{s}=\frac{1}{n}\sum_{j=1}^{n}a_{s}^{\left(j\right)},\\ & {\bar{b}}_{s}=\frac{1}{n}\sum_{j=1}^{n}b_{s}^{\left(j\right)}, \end{array}$$

we have the condition for *ρ*_*A*_ > *ρ*_*B*_, given by

$$\begin{array}{c} \sum_{s=0}^{g}{\tilde{\sigma }}_{s}({\bar{a}}_{s}-{\bar{b}}_{g-s})>0. \end{array}$$ (5)

Note that ${\bar{a}}_{s}$ (${\bar{b}}_{s}$) corresponds to the payoff averaged over all games when there are *s* opposing cooperators. This further supports that while payoff structures are diverse in different interactions, the evolutionary outcome can be predicted by assuming that all interactions are governed by a unified payoff structure, i.e., the ‘average’ over all structures. Alternatively, we can rewrite Eq (4) as $\sum_{j=1}^{n}[\sum_{s=0}^{g}{\tilde{\sigma }}_{s}(a_{s}^{\left(j\right)}-b_{g-s}^{\left(j\right)})]>0$. Note that $\sum_{s=0}^{g}{\tilde{\sigma }}_{s}(a_{s}^{\left(j\right)}-b_{g-s}^{\left(j\right)})$ presents the results when all interactions described by the single payoff structure, i.e., $a_{s}^{\left(j\right)}$ and $b_{s}^{\left(j\right)}$. Therefore, the evolutionary outcome under diverse multiplayer games can be viewed as the sum of results obtained when all interactions are governed by a single payoff structure. Both the two interpretations significantly simplify the calculation complexity when the payoff forms are relation-dependent. We also confirm the above findings in infinite populations (see S1 File, Section 3).

**Fig 2:**
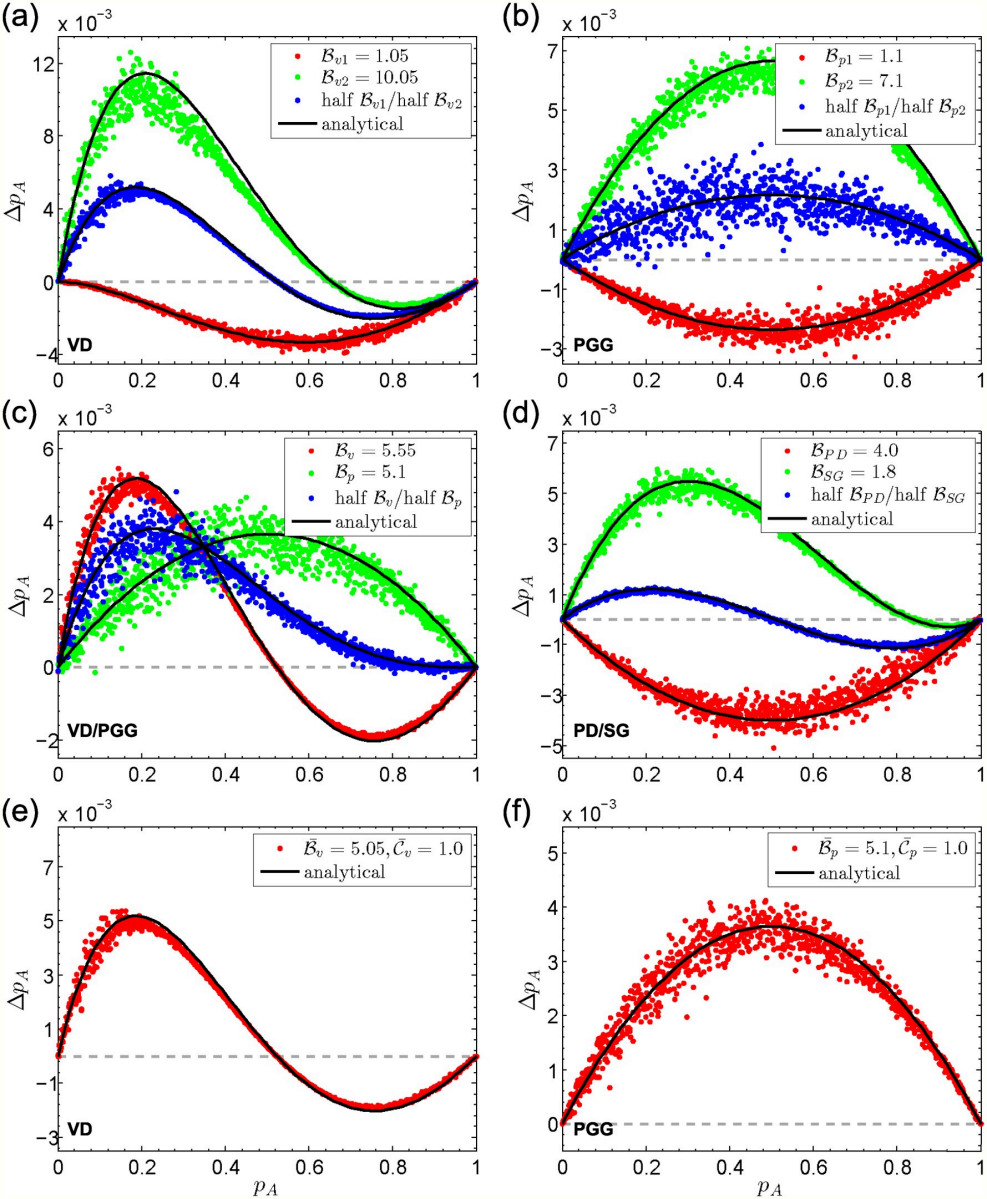
Average change (Δ*p*_A_) in the frequency of A-players (*p*_A_), in volunteer’s dilemmas (VD), public goods games (PGG), diverse multiplayer games (VD/PGG), and diverse two-player games (PD/SG). The population structure is a random regular graph with *N* = 1000, *n* = 2, and *g*_1_ = *g*_2_ = 3. In (a-c), the cost to cooperate is fixed to 1 and benefits are shown in the legend of each panel. In (d), under **PD**, a cooperator bears a cost 1 to provide its opponent with a benefit $B_{PD}$. Under each **SG**, the total cost for cooperators is 1 and the benefit for each player is $B_{SG}$. Three cases are investigated in each panel of (a-d). For example, in (a), benefits in all multiplayer interactions are $B_{v1}=1.05$ (red dots), $B_{v2}=10.05$ (green dots), or designated at equal proportions (blue dots). In (ef), both benefits and costs in each interaction are sampled according to a Gaussian distribution, with mean ${\bar{B}}_{v}=5.05$, variance 1.5 for benefits and mean ${\bar{C}}_{v}=1.0$, variance 0.25 for costs (e), mean ${\bar{B}}_{p}=5.1$ and variance 1.5 for benefits and mean ${\bar{C}}_{p}=1.0$ and variance 0.25 for costs (f). Dots represent the simulation data and lines are analytical predictions based on unified interactions with average payoffs (see S1 File, Section 6 for simulation details).

We illustrate a few examples in Fig 2, including nonlinear multiplayer game like volunteer’s dilemmas [57] and linear public goods games. In a volunteer’s dilemma, once an individual volunteers by bearing a cost $C_{v}$, each participant obtains a benefit $B_{v}$. In Fig 2a, each individual participates in two volunteer’s dilemmas in each generation. When $B_{v}=1.05$ and $C_{v}=1$ in one interaction and $B_{v}=10.05$ and $C_{v}=1$ in the other, the evolutionary dynamics can be approximated by the case where all interactions are described by a unified volunteer’s dilemma with $B_{v}=\left(1.05+10.05\right)/2$ and $C_{v}=\left(1+1\right)/2$. Alternatively, dynamics for the case with half $B_{v1}=1.05$ and half $B_{v2}=10.05$ (blue) can be viewed as the average over that with full $B_{v1}=1.05$ (red) and that with full $B_{v2}=10.05$ (green). In the public goods game, each cooperator makes an investment $C_{p}$ to yield a benefit $B_{p}$ and then the benefit is evenly distributed over all participants. Panels Fig 2b and 2c confirm above findings in linear public goods games and mixed games (half volunteer’s dilemmas and half linear public goods games).

We highlight above rules can be further extended to more general cases. When each collective interaction is endowed with an independent payoff structure (payoff structures in any two interactions centered on player *x* are independent; besides, payoff structures in any interaction centered on player *x* and those centered on *y* are uncorrelated), the collective behavior still can be predicted by an ‘average’ case over all interactions (see panels Fig 2e and 2f). Furthermore, if the numbers of participants in different collective interactions are not identical, interactions with the same number of participants can be described by their ‘average’ case. That is, if $g_{l_{1}}=g_{l_{2}}=\cdots =g_{l_{u}}\neq g_{m_{1}}=g_{m_{2}}=\cdots =g_{m_{v}}$, interactions with individuals linked by edges of type *l*_1_, *l*_2_, ⋯, *l*_*u*_ can be resolved as uniform interactions with payoff matrix ${\bar{a}}_{s}^{\left(l\right)}=\sum_{j=l_{1}}^{l_{u}}a_{s}^{\left(j\right)}/u$ and ${\bar{b}}_{s}^{\left(l\right)}=\sum_{j=l_{1}}^{l_{u}}b_{s}^{\left(j\right)}/u$. Interactions associated with edges of type *m*_1_, *m*_2_, ⋯, *m*_*v*_ can be treated as uniform interactions with payoff matrix ${\bar{a}}_{s}^{\left(m\right)}=\sum_{j=m_{1}}^{m_{v}}a_{s}^{\left(j\right)}/v$ and ${\bar{b}}_{s}^{\left(m\right)}=\sum_{j=m_{1}}^{m_{v}}b_{s}^{\left(j\right)}/v$, applicable to sufficiently large finite and infinite populations. Generally, if there are *m* different game sizes among *n* multiplayer games, i.e., *g*_1_, *g*_2_, ⋯, *g*_*m*_, satisfying *g*_*i*_ ≠ *g*_*j*_ if *i* ≠ *j* (1 ≤ *i*, *j* ≤ *m*), the number of structure coefficients needed to describe the effects of population structures decreases to $\sum_{i=1}^{m}\left(g_{i}+1\right)$. Therefore, in the absence of edge diversity (thus *m* = 1 and *g*_1_ = *k*), the number of structure coefficients is *k* + 1, in line with a previous study [33]. If game sizes for all multiplayer games are different (thus *m* = *n*), we need $\sum_{i=1}^{n}\left(g_{i}+1\right)$ structure coefficients to predict the evolutionary outcome. Table 2 summarizes the number of structure coefficients in various cases.

**Table 2:** The number of structure coefficients to predict the evolutionary outcome.

	general payoff structure	payoff structure of diverse multiplayer games
general spatial structure	$\Pi_{i=1}^{m}\left(\begin{array}{c} g_{i}+n_{i}\\ n_{i} \end{array}\right)$	$\sum_{i=1}^{m}\left(g_{i}+1\right)$
*g*_*i*_ = *g* for any 1 ≤ *i* ≤ *n*	$\left(\begin{array}{c} g+n\\ n \end{array}\right)$	*g* + 1
*g*_*i*_ ≠ *g*_*j*_ for any *i* ≠ *j*	$\Pi_{i=1}^{n}\left(g_{i}+1\right)$	$\sum_{i=1}^{n}\left(g_{i}+1\right)$

In the general spatial structure, there are *m* different values among all *g*_*i*_s (1 ≤ *i* ≤ *n*). We denote *g*_1_, *g*_2_, ⋯, *g*_*m*_ these values and *n*_*i*_ the number of value *g*_*i*_, i.e., $k=\sum_{i=1}^{m}n_{i}g_{i}$. Note that we can further eliminate an extra structure coefficient through dividing the sigma rule [see Eq (1)] by a positive structure coefficient.

#### Example 3. Evolutionary multiplayer games on weighted graphs

**Fig 3:**
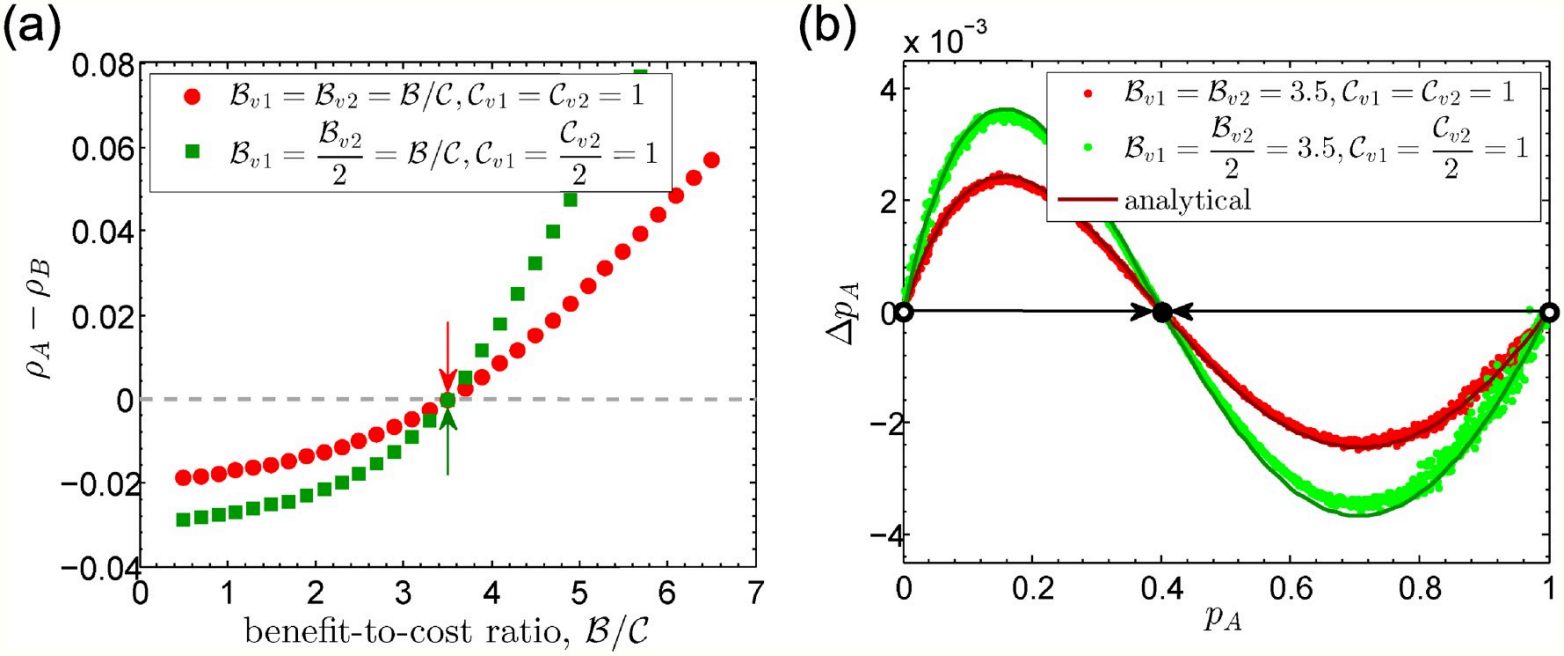
Evolutionary multiplayer games on weighted graphs. Each individual participates in two volunteer’s dilemmas and both group sizes are 3. Benefits and costs are $B_{v1}$ and $C_{v1}$ for one dilemma, $B_{v2}$ and $C_{v2}$ for the other. (a) Difference in fixation probability *ρ*_A_ − *ρ*_B_ as a function of benefit-to-cost ratio (B/C). (b) Average change (Δ*p*_A_) in the frequency of *A*−players (*p*_A_). Arrows in (a) mark the analytical benefit-to-cost ratio ${\left(B/C\right)}^{*}$ and solid lines in (b) represent analytical change in *p*_A_. Dots represent the simulation data (see S1 File, Section 6 for simulation details). Heterogeneous weights of edges do not change the critical benefit-to-cost ratio ${\left(B/C\right)}^{*}$ in the finite population (a) or the inner equilibria (black point) in the infinite population (b).

Interactions between individuals often differ in capacity, frequency, and strength [60]. Weighted graphs well incorporate these factors where weights of edges are proportional to interaction frequencies. Partly due to the simple and intuitive understanding of weighted edges, most studies about games on weighted graphs so far are based on pairwise interactions [23, 29, 61–63]. Although collective interactions can also occur at different interaction rates like two-player versions, few studies explore it. The framework proposed in this paper is also applicable to investigate the multiplayer games on weighted graphs, where different group interactions occur at different rates. Concretely, individuals linked by the same type of edges are engaged in a group interaction and these edges are endowed with a uniform weight which represents the frequency of this group interaction. Thus, a larger value of edge weight means the more frequent contact [23, 29, 61–63] or more diffusible public goods between interactants [53, 64]. Counter-intuitively, we show that strong social ties do not change the evolutionary fate of cooperation, irrespective of based on multiplayer or two-player games (see S1 File, Section 4). As shown in Fig 3a, in finite populations, strong social ties just amplify the difference in fixation probabilities (*ρ*_*A*_ − *ρ*_*B*_) while keep the critical condition B/C for *ρ*_*A*_ > *ρ*_*B*_ unchanged. Analogously, in infinite populations, strong social ties accelerate the evolutionary rate while they do not change the interior equilibria at all (Fig 3b). We can make this clear by virtue of conclusions in Example 2. In volunteer’s dilemmas, the payoff structure for interactions with individuals linked by edges of type *j* is $a_{s}^{\left(j\right)}=B_{v}^{\left(j\right)}-C_{v}^{\left(j\right)}$ for any *s*, $b_{s}^{\left(j\right)}=B_{v}^{\left(j\right)}$ for *s* > 0, and $b_{s}^{\left(j\right)}=0$ for *s* = 0. We take $B_{v}^{\left(j\right)}=\zeta_{j}B_{v}$ and $C_{v}^{\left(j\right)}=\zeta_{j}C_{v}$, where *ζ*_*j*_ denotes the weight of edges linking individuals of type *j*. From Example 2, the evolutionary dynamics can be approximated by unified interactions with payoff structure ${\bar{a}}_{s}={\bar{B}}_{v}-{\bar{C}}_{v}$ for any *s*, ${\bar{b}}_{s}={\bar{B}}_{v}$ for *s* > 0, and ${\bar{b}}_{s}=0$ for *s* = 0, where ${\bar{B}}_{v}=\sum_{j=1}^{n}B_{v}^{\left(j\right)}/n=B_{v}\sum_{j=1}^{n}\zeta_{j}/n$ and ${\bar{C}}_{v}=C_{v}\sum_{j=1}^{n}\zeta_{j}/n$. Combining Eq (5), we have the critical condition

$$\begin{array}{c} {\left(\frac{B_{v}}{C_{v}}\right)}^{*}=\frac{1}{{\tilde{\sigma }}_{g}} \end{array}$$

above which *ρ*_*A*_ > *ρ*_*B*_. Note that ${\left(B_{v}/C_{v}\right)}^{*}$ is independent of edge weights $\sum_{j=1}^{n}\zeta_{j}$.

#### Example 4. Multiplayer public goods game with division of labor

In above examples, we assume that each individual participates in many independent games and its payoff is the linear accumulation of payoffs derived in different games. But in more complicated situations, different types of neighbors affect the focal individual in a nonlinearly coupling way. It is an oversimplification if we treat the interaction between one player and different types of neighbors as the sum of several independent games. The activity of labor division in colonies of eusocial insects and human societies is a typical example. We consider a team of army ants retrieving prey items. They can do this successfully only if different kinds of ants coordinate to perform corresponding subtasks [46]. In other words, cooperation from each kind of individuals is required to produce public goods. We consider the simplest case with two kinds of individuals and the production of benefits requires at least one cooperator within each kind. We use two types of edges on graphs to model this case: edges of type 1 link the same kind of individuals and edges of type 2 link different kinds of individuals. A player obtains benefits only if in its neighborhood there are cooperative individuals along two types of edges. We consider the evolution of individuals’ behaviors (cooperation and defection) and we assume that individuals’ subtasks are fixed throughout the evolutionary process. Payoff values are given by

$$\begin{array}{c} a_{s_{1}s_{2}}=\{\begin{array}{ccc} (s_{1}+s_{2}+1)B-C & & s_{2}\geq 1,\\ -C & & \text{otherwise}, \end{array} \end{array}$$ (6)

$$\begin{array}{c} b_{s_{1}s_{2}}=\{\begin{array}{ccc} (s_{1}+s_{2})B & & s_{1}\geq 1,s_{2}\geq 1,\\ 0 & & \text{otherwise}, \end{array} \end{array}$$ (7)

where C means the personal cost for each cooperator and B is the benefit to each participant. Note that *a*_01_ is not necessarily identical to *a*_10_. The public goods increase linearly with the number of cooperators, inasmuch as the number exceeds the corresponding threshold, termed accumulative effects of payoffs. Substituting Eqs (6) and (7) into Eq (1), we have the critical benefit-to-cost ratio ${\left(B/C\right)}^{*}$ above which cooperation is favored over defection, given by

$$\begin{array}{c} {\left(B/C\right)}^{*}=\frac{1}{\sum_{s_{1}=0}^{g_{1}}\sum_{s_{2}=1}^{g_{2}}\left(s_{1}+s_{2}+1\right)\sigma_{s_{1}s_{2}}-\sum_{s_{1}=0}^{g_{1}-1}\sum_{s_{2}=0}^{g_{2}-1}\left(k-s_{1}-s_{2}\right)\sigma_{s_{1}s_{2}}}. \end{array}$$

Then we consider the scenario without division of labor. That is, benefits are produced as long as the total number of cooperators reaches a threshold. For comparison, we set the threshold to be 2. Payoffs are thus $a_{s}=\left(s+1\right)B-C$ if *s* ≥ 1 and $a_{s}=-C$ otherwise; $b_{s}=sB$ if *s* ≥ 2 and *b*_*s*_ = 0 otherwise. ${\left(B/C\right)}^{*}$ derived from Eq (1) is

$$\begin{array}{c} {\left(B/C\right)}^{*}=\frac{1}{\sum_{s=1}^{k}\left(s+1\right)\sigma_{s}-\sum_{s=0}^{k-2}\left(k-s\right)\sigma_{s}}. \end{array}$$

Furthermore, we explore the case that the public goods remain fixed as the number of cooperators increases, inasmuch as the number exceeds the corresponding threshold (thus without accumulative effects of payoffs). Payoffs are given by

$$\begin{array}{c} a_{s_{1}s_{2}}=\{\begin{array}{ccc} B-C & & s_{2}\geq 1,\\ -C & & \text{otherwise}, \end{array} \end{array}$$

$$\begin{array}{c} b_{s_{1}s_{2}}=\{\begin{array}{ccc} B & & s_{1}\geq 1,s_{2}\geq 1,\\ 0 & & \text{otherwise}. \end{array} \end{array}$$

We thus have

$$\begin{array}{c} {\left(B/C\right)}^{*}=\frac{1}{\sum_{s_{1}=0}^{g_{1}}\sum_{s_{2}=1}^{g_{2}}\sigma_{s_{1}s_{2}}-\sum_{s_{1}=0}^{g_{1}-1}\sum_{s_{2}=0}^{g_{2}-1}\sigma_{s_{1}s_{2}}}. \end{array}$$

Analogously, in the counterpart with no labor division, if we set a single threshold 2, payoffs are $a_{s}=B-C$ if *s* ≥ 1 and $a_{s}=-C$ otherwise; $b_{s}=B$ if *s* ≥ 2 and *b*_*s*_ = 0 otherwise. We have ${\left(B/C\right)}^{*}$

$$\begin{array}{c} {\left(B/C\right)}^{*}=\frac{1}{\sum_{s=1}^{k}\sigma_{s}-\sum_{s=0}^{k-2}\sigma_{s}}. \end{array}$$

**Fig 4:**
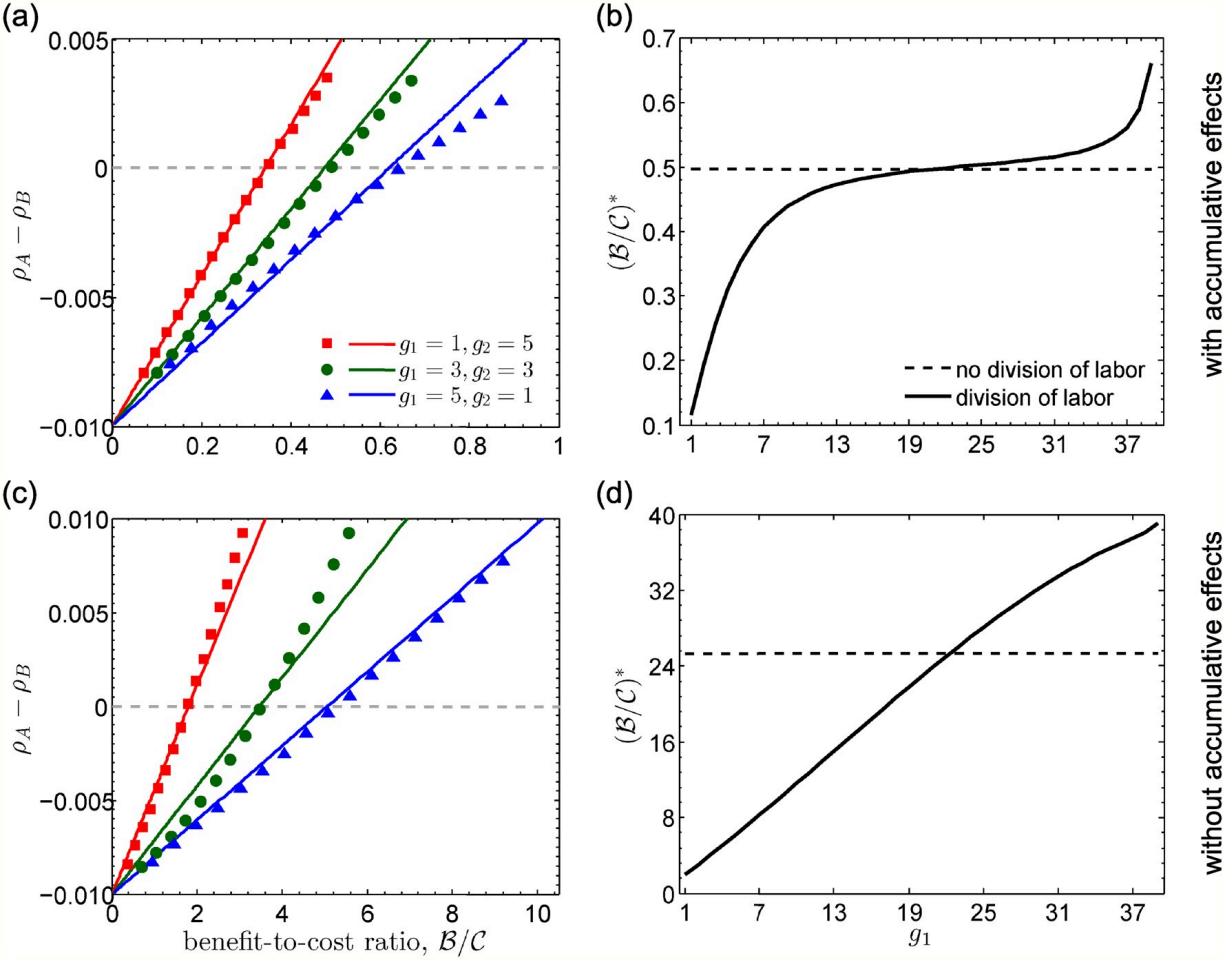
Difference in fixation probability *ρ*_A_-*ρ*_B_ and critical benefit-to-cost ratio ${\left(B/C\right)}^{*}$ for *ρ*_A_ > *ρ*_B_ as a function of *g*_1_. (ab) Division of labor with accumulative effects of payoffs (the increasing number of cooperators leads to the increasing productivity). (cd) Division of labor without accumulative effects of payoffs (the productivity remains unchanged as the increasing number of cooperators). In (a) and (c), we consider *n* = 2 and different parameters of *g*_1_ and *g*_2_. Dots presents simulation data (see S1 File, Section 6 for simulation details) and lines are analytical predictions. *ρ*_A_ − *ρ*_B_ is analytically predicted by the product of the left side of Eq (1) and the selection intensity *ω*. In (b) and (d), dash lines correspond to ${\left(B/C\right)}^{*}$ for the case with no division of labor and solid lines ${\left(B/C\right)}^{*}$ for the case with division of labor, where *g*_2_ = 40 − *g*_1_.

Panels Fig 4a and 4c show that analytical predictions of fixation probabilities are in good agreement with results by Monte Carlo simulations for the whole range of benefit-to-cost ratios and for different parameters of *g*_1_ and *g*_2_. In Fig 4b, we show that with division of labor, ${\left(B/C\right)}^{*}$ is a monotonous function of *g*_1_. Surprisingly, for small *g*_1_, i.e., *g*_1_ = 1, ${\left(B/C\right)}^{*}$ is much lower than that without introducing division of labor. Furthermore, for large *g*_1_, i.e., *g*_1_ = 39, ${\left(B/C\right)}^{*}$ is far larger than that without introducing division of labor. Therefore, the introduction of division of labor could significantly lower the barrier to establish a cooperative society, given a small number of individuals assigned the same subtask. These findings are further confirmed when the increasing cooperation does not lead to the increasing productivity (see Fig 4d).

To make these explicit, we consider a case with a sufficiently large *k* and without accumulative effects of payoffs. With no division of labor (abbreviated to “ndol”), payoffs of A- and B-players are respectively given by

$$\begin{array}{cc} \pi_{\text{A}}^{\text{ndol}} & =[1-r^{k}{\left(1-p_{\text{A}}\right)}^{k}]B-C,\\ \pi_{\text{B}}^{\text{ndol}} & =[1-{\left(1-rp_{\text{A}}\right)}^{k-1}(1+kp_{\text{A}}-2p_{\text{A}})]B, \end{array}$$

where *r* = (*k* − 2)/(*k* − 1) and *p*_A_ is the fraction of A-players. With division of labor (abbreviated to “dol”), payoffs of A- and B-players are

$$\begin{array}{cc} \pi_{\text{A}}^{\text{dol}} & =[1-r^{g_{2}}{\left(1-p_{\text{A}}\right)}^{g_{2}}]B-C,\\ \pi_{\text{B}}^{\text{dol}} & =[1-{\left(1-rp_{\text{A}}\right)}^{g_{1}}][1-{\left(1-rp_{\text{A}}\right)}^{g_{2}}]B. \end{array}$$

**Fig 5:**
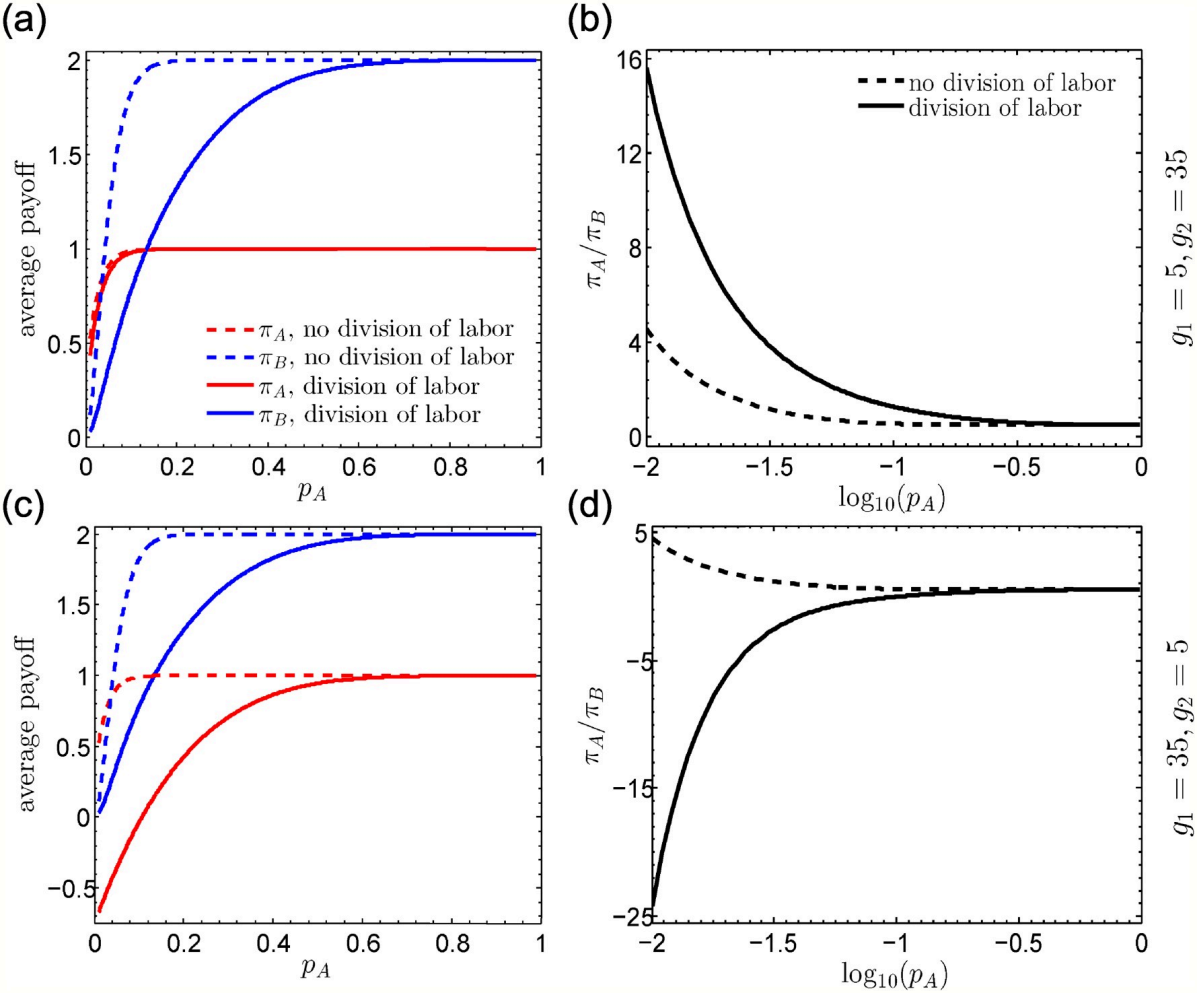
Average payoffs as a function of *p*_A_ with no division of labor (dash lines) and with division of labor (solid lines). Here the increasing number of cooperators does not lead to increasing productivity, inasmuch as the number exceeds the threshold. (a) For *g*_1_ = 5 and *g*_2_ = 35, division of labor does not affect the average payoff of A-players (*π*_A_) much while it reduces the average payoff of B-players (*π*_B_) significantly. This increases *π*_A_/*π*_B_ for the whole range of *p*_A_ (b), and thus weakens the advantages of defectors over cooperators. (c) For *g*_1_ = 35 and *g*_2_ = 5, division of labor reduces both *π*_A_ and *π*_B_ remarkably. Nevertheless, the impact to *π*_A_ is more noticeable than to *π*_B_ (d) and thus reinforces the advantages of defectors over cooperators. We take B=2 and C=1.

For 0 < *p*_A_ < 1, we have $\pi_{\text{A}}^{\text{dol}}<\pi_{\text{A}}^{\text{ndol}}$ and $\pi_{\text{B}}^{\text{dol}}<\pi_{\text{B}}^{\text{ndol}}$. Thus division of labor transiently reduces the average payoffs of both A- and B-players. This result is understandable since with the labor division the condition of producing benefits becomes more stringent. However, in terms of the long-term development and stable states, the labor division is beneficial to the evolving system. The labor division actually influences the competition between different strategic behaviors and ultimately contributes to a cooperative society, which appears to be more prosperous. To evaluate how the division of labor influences the competition between A- and B-players, we compare $\pi_{\text{A}}^{\text{dol}}/\pi_{\text{B}}^{\text{dol}}$ with $\pi_{\text{A}}^{\text{ndol}}/\pi_{\text{B}}^{\text{ndol}}$. If $\pi_{\text{A}}^{\text{dol}}/\pi_{\text{B}}^{\text{dol}}>\pi_{\text{A}}^{\text{ndol}}/\pi_{\text{B}}^{\text{ndol}}$ ($\pi_{\text{A}}^{\text{dol}}/\pi_{\text{B}}^{\text{dol}}<\pi_{\text{A}}^{\text{ndol}}/\pi_{\text{B}}^{\text{ndol}}$), division of labor enhances (weakens) the advantage of A-players relative to B-players compared with that under no division of labor. For *g*_1_ ≪ *k*, $\pi_{\text{A}}^{\text{dol}}$ approaches to $\pi_{\text{A}}^{\text{ndol}}$, indicating the impact of division of labor to A-players is negligible (see Fig 5a). $\pi_{\text{B}}^{\text{dol}}$ is the product of two terms (except B). One term, $1-{\left(1-rp_{\text{A}}\right)}^{g_{2}}$, corresponds to the probability that there are cooperators among players belonging to a different type, roughly approximating to $\pi_{\text{B}}^{\text{ndol}}/B$. The other term, $1-{\left(1-rp_{\text{A}}\right)}^{g_{1}}$, is the probability that there exist cooperators among players whose types are the same as the focal player. For *g*_1_ ≪ *k*, this term dominates the loss to B-players and weakens the advantages of defectors over cooperators. The form of $\pi_{\text{B}}^{\text{dol}}$ implies that the division of labor essentially transforms a many-player game into two coupled fewer-player games, i.e., one game in which all participants show the same type as the focal player and one game in which participants’ types are different from the focal player. When the focal player belongs to a smaller group, it is harder to free-ride on others, which makes clear positive effects of division of labor on cooperation thriving. Scenarios for *g*_2_ ≪ *k* can be analyzed analogously (see panels Fig 3c and 3d). This conclusion is still true with *n* > 2 types of edges (see S2 Fig). Our results suggest that the more specialized individuals are, namely, the less individuals are of the same type, the more cooperation will be achieved. This may explain the flourishing cooperation in the highly specialized human societies.

## Discussion

Due to variations in environment or gene, individuals own distinct social status or play different roles in colonies [65, 66]. Typically, individuals with geographic proximity and genetic similarity tend to establish closer social ties than those separated by remote geographic space or distinguished by large genetic difference. Encountering different types of individuals, one may be affected differently. Here we model the heterogeneous influence by different types of edges and develop a framework of evolutionary multiplayer games on graphs with edge diversity. Since the two-player game is the simplest multiplayer game, our findings are applicable to pairwise interactions. We make a thorough investigation in both finite and infinite populations. We provide the analytical formula of structure coefficients for random regular graphs with *n* types of edges, which effectively predicts when natural selection favors one strategic behavior over the other.

Our framework is able to address the situation where individuals concurrently face diverse social dilemmas. This is in stark contrast with the ideal assumption in most previous studies where all interactions are described by a unified game metaphor [19–22, 31–33]. In the real word, an individual may be caught in a volunteer’s dilemma with its colleagues and meanwhile it engages in public goods games with its neighbors. The tragedy of commons suggests that cooperation is often hard to persist in the public goods game. Fortunately, the public goods game is merely one of the many types of social dilemmas individuals encounter. Our work reveals that leveraging the distinct nature of diverse social dilemmas can entail an evolutionary outcome where cooperators are rescued and are able to coexist with defectors. In addition, a seminal work by McAvoy *et. al*. tells that under asymmetric two-player games the evolutionary processes behave macroscopically like that governed by symmetric games [59]. Here we confirm that irrespective of two-player or multiplayer games, the evolutionary dynamics with diverse interactions can be approximated by that governed by a single game. For more complicated cases where sizes of group interactions are different, we also provide an efficient method to simplify it. Our work greatly reduces the complexity when investigating the evolutionary dynamics in real-world systems.

Besides, multiplayer games on weighted graphs can be considered. We find that the presence of strong social ties does not always provide an evolutionary advantage to cooperators, which seems to coincide with recent findings under aspiration dynamics [63]. This contrasts with the conclusion in Ref. [23] where they show that strong ties boost cooperation most. The main difference between our work and theirs is that we do not couple the strength of interactions and the probability of replacement along an edge. In their work, a strong social tie indicates not only a higher frequency of interactions but also a more probable path for strategy dispersal. Simultaneously enhancing the strength of interactions and the likelihood of dispersal leads to a strong strategy reciprocity between individuals and thus facilitates the clustering of cooperators. However, if strong ties merely indicate frequent interactions as in our work, we show that they fail to promote cooperation, irrespective of group or pairwise interactions. Note that in our model, individuals derive payoffs only from interactions with their nearest neighbors [33, 54]. When individuals can interact with both the nearest and second-nearest neighbors, the impact of social ties on the evolution of cooperation are more complicated [64]. A further investigation along this direction may generate new insights. A prior study has considered that players’ social influence affects the strategy dispersal, which in turn modifies players’ social influence [67]. Such a coevolution actually corresponds to a dynamic structure for strategy dispersal, different from the static structure in this paper.

Within this framework, we consider how the division of labor affects the evolution of cooperation. As well known, the division of labor prevails in colonies of social insects, hunting groups of lions, and human societies [45–48, 52], where individuals are born or trained to perform specialized subtasks. Such specialization not only makes them more productive on their own subtasks but also results in synergistic effects on the overall productivity when they cooperate with each other. We here model the strategic interactions under the division of labor as a multi-threshold public goods game. The public goods are provided only when individuals of distinct types cooperate. We find that the division of labor could promote the evolution of cooperation. The reason lies in that task specialization transforms a many-player interaction into several coupled fewer-player interactions. Such a transformation helps to reduce the free-riding behaviors.

Our work also extends the research scope about the interplay between the evolution of a population and the diversity. The two basic elements of a population are individuals and social ties. Most prior studies about diversity focus on individuals’ attributes, such as the number of social ties they have, the ability to influence their opponents, etc [68, 69]. Such diversity highlights that two individuals are different when possessing different attributes. Here we stress the diversity of social ties. Social ties not just establish the connections between separated individuals. They carry a massive amount of information about two connected individuals, such as the intimacy of the interpersonal relationships, the frequency of physical contact, and even the history about previous interactions. All these are unlikely to be captured by individuals’ attributes. The example of division of labor also proved that the diversity of social ties (or edge diversity) could catalyze cooperation. Our recent work about interactive diversity is pertinent to this topic [24, 70]. Interactive diversity describes that each individual adopts independent strategies in different interactions. Thus even facing an identical strategy by two different opponents, the focal individual could be influenced differently due to its own behavior. Nevertheless, the influence difference fully depends on strategies between interactants and is unrelated to other information like genetic similarity or geographic proximity. Thus, interactive diversity does not essentially capture diverse social ties explored in this paper [37]. We wish our work could attract more work into the evolutionary dynamics along edges.

Here we stress that edge diversity proposed in this paper is different from edge multiplexity, an important terminology in social networks [71]. Although both edge multiplexity and edge diversity describe the association between individuals rather than individuals’ attributes, they have different implications. Edge multiplexity means that the relationship between two individuals is multiplex when they interact in multiple social contexts. For example, two individuals can be both friends and colleagues. But edge diversity, simply speaking, means that edges are different. Specifically, in our work, it means that different edges may carry different social, physical or genetic information between individuals, such as the interaction frequency (or rate), geographic distance or genetic similarity. It highlights the differences between edges rather than the multiplexity of the associated relationship. For example, even if an individual shares the same multiplex relationship with two other partners, due to the distinct interaction rates, the two social ties are still interpreted as different edges in terms of edge diversity.

In this paper we constrain that each social tie has symmetric effects on connected individuals. For example, if Alice is close to Bob in consanguinity or geographic sites, Bob is close to Alice. Thus the benefit that cooperative Alice brings to Bob is identical to that of cooperative Bob to Alice. A promising and challenging extension is the interactions with asymmetric social ties, such as the relationship between leaders and followers. In such case, each individual should be endowed with an independent payoff function [10, 72]. Despite much complicity in analytical calculations, we expect a further research into this realistic situation, which is bound to provide fruitful insights. We point out that our theoretical results are based on assumption of weak selection, as used by most previous theoretical studies [19–22, 32, 33, 35]. Although the assumption of weak selection is reasonable in many cases and also make this conundrum accessible to analytical calculation [73], other situations routinely encountered in social or natural science are better captured by strong selection. Thus, a further investigation with strong selection is necessary to enrich our understanding to the collective behavior in complex systems [36, 74]. Finally, in this paper, we assume that the types of edges remain unchanged throughout the evolution. This is natural in many cases, like when types of edges indicate the geographic proximity. Nevertheless, when edges’ types represent the genetic difference between linked individuals and the population evolve based on individuals’ reproduction, edges’ types evolve as well [59]. A study into the coevolution of individuals’ traits and edge types is expected.

## Supporting information

S1 Fig Analytical fixation probability is in good agreement with simulation results. Solid lines present the analytical fixation probability of cooperators (*ρ*_*A*_) and dash lines show the analytical fixation probability of defectors (*ρ*_*B*_). Dots show results by computer simulations (see S1 File, Section 6 for simulation details). Parameters in (a) follow Fig 2a and parameters in (b) follow Fig 2c.
(TIF)

S2 Fig Division of labor could reduce the free-riding behaviors for *n* > 2. On graphs with *n* types of edges, the production of benefits requires cooperation from players linked by each type of edges. Note that the focal player and its neighbors linked by edges of type 1 play the same role in producing benefits. Here the increasing number of cooperators does not lead to the increasing productivity, inasmuch as the number exceeds the threshold. (a) Difference between r* with division of labor (“dol”) and with no division of labor (“ndol”). *n* = 3 and *g*_1_ + *g*_2_ + *g*_3_ = 40. The upper right zone is invalid given a positive *g*_3_. The block dots present the configurations of *g*_1_ and *g*_2_ for which r*s with division of labor and those with no division of labor are nearly equal. (b) r* as a function of *n*. We fix ∑_1≤*i*≤*n*_ *g*_*i*_ = 40, *g*_*i*_ = 5 for 2 ≤ *i* ≤ *n* − 1, and vary *g*_1_. Both (a) and (b) show that a small value of *g*_1_ facilitates cooperation.
(TIF)

S3 FigAnalytical results qualitatively predict the evolutionary outcomes in a real-world friendship network.The details about this network are provided in S1 File, Section 5. This network consists of 2539 nodes and two types of edges, i.e., type 1 and type 2. On average, each node is linked to 4.3 other nodes by edges of type 1 and 4 other nodes by edges of type 2. In each generation, each player plays a volunteer’s dilemma with neighbors linked by edges of type 1 and it also plays a public goods game with neighbors linked by edges of type 2. Dots represent the simulation data (see S1 File, Section 6 for simulation details). Lines are analytical predictions based on Eq (2) in the main text. $C_{v}=1$ and $C_{p}=1$. Other parameter values: $B_{v}=1.1$ and $B_{v}=2$ (a); $B_{v}=8$ and $B_{v}=2$ (b); $B_{v}=8$ and $B_{v}=8$ (c). We use *g*_1_ = 4 and *g*_2_ = 4 in the theoretical calculations.(TIF)

S1 File Theoretical deviations. Calculations of fixation probabilities and structure coefficients for evolutionary multiplayer games on graphs with *n* types of edges in finite populations. Derivations of the replication equation for evolutionary multiplayer games on graphs with *n* types of edges in infinite populations.
(PDF)

S2 File Software code and data. (RAR)
