# Supplementary figures and images for: Evolutionary multiplayer games on graphs with edge diversity
Source: PLoS Comput Biol. 2019 Apr 1;15(4):e1006947. doi: 10.1371/journal.pcbi.1006947 (PMC6459562; doi:10.1371/journal.pcbi.1006947)

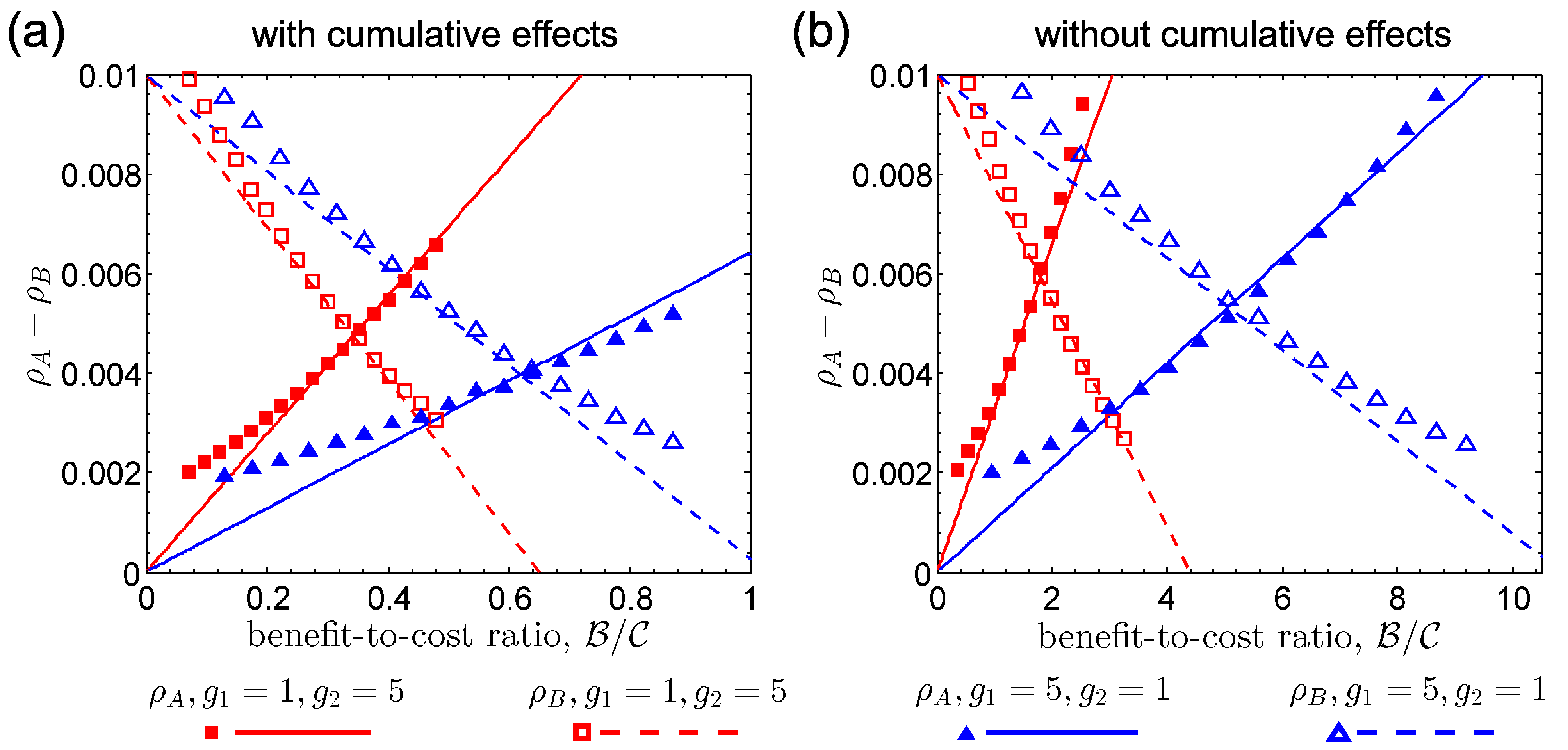

Supplement: S1 Fig — Solid lines present the analytical fixation probability of cooperators (ρA) and dash lines show the analytical fixation probability of defectors (ρB). Dots show results by computer simulations (see S1 File, Section 6 for simulation details). Parameters in (a) follow Fig 2a and parameters in (b) follow Fig 2c. (TIF) [file pcbi.1006947.s001.tif]

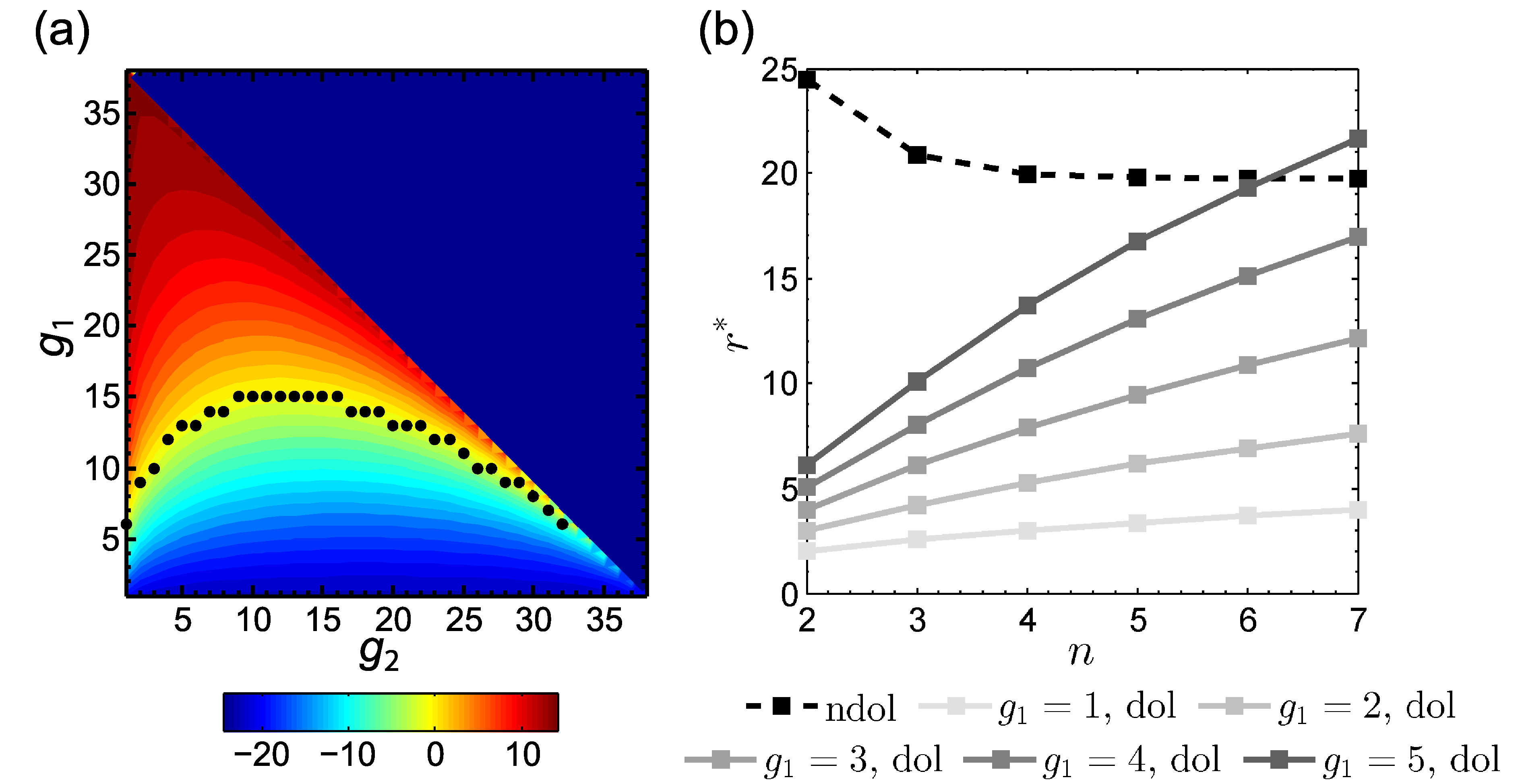

Supplement: S2 Fig — On graphs with n types of edges, the production of benefits requires cooperation from players linked by each type of edges. Note that the focal player and its neighbors linked by edges of type 1 play the same role in producing benefits. Here the increasing number of cooperators does not lead to the increasing productivity, inasmuch as the number exceeds the threshold. (a) Difference between r* with division of labor (“dol”) and with no division of labor (“ndol”). n = 3 and g1 + g2 + g3 = 40. The upper right zone is invalid given a positive g3. The block dots present the configurations of g1 and g2 for which r*s with division of labor and those with no division of labor are nearly equal. (b) r* as a function of n. We fix ∑1≤i≤n gi = 40, gi = 5 for 2 ≤ i ≤ n − 1, and vary g1. Both (a) and (b) show that a small value of g1 facilitates cooperation. (TIF) [file pcbi.1006947.s002.tif]

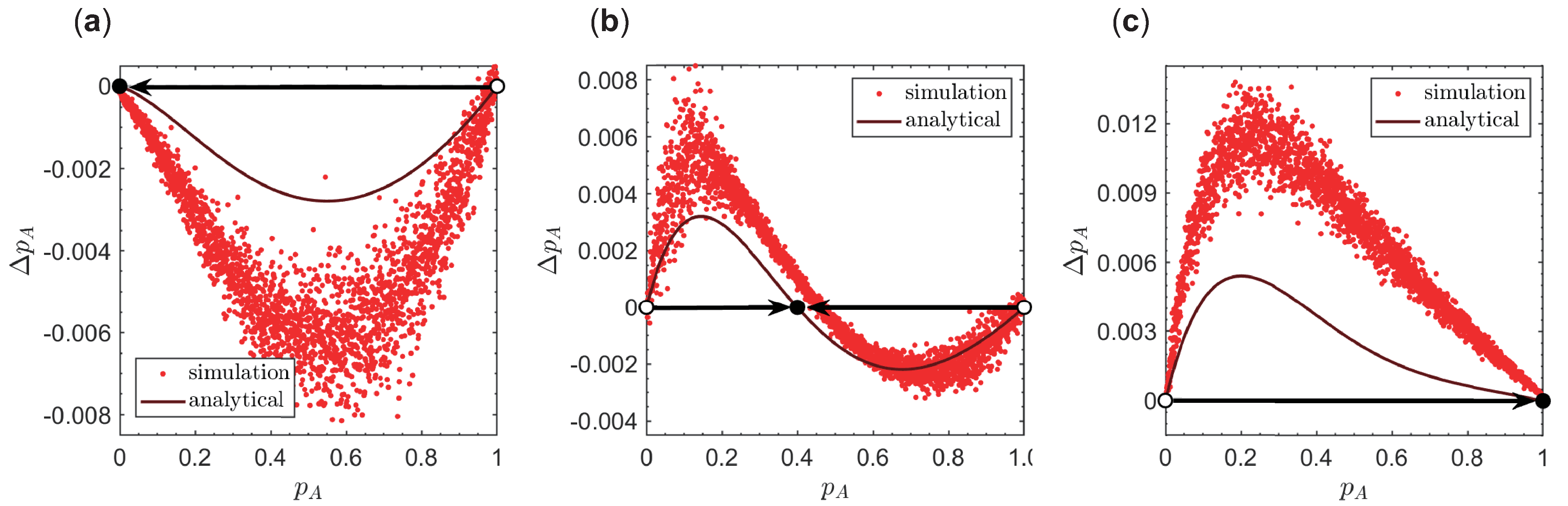

Supplement: S3 Fig — The details about this network are provided in S1 File, Section 5. This network consists of 2539 nodes and two types of edges, i.e., type 1 and type 2. On average, each node is linked to 4.3 other nodes by edges of type 1 and 4 other nodes by edges of type 2. In each generation, each player plays a volunteer’s dilemma with neighbors linked by edges of type 1 and it also plays a public goods game with neighbors linked by edges of type 2. Dots represent the simulation data (see S1 File, Section 6 for simulation details). Lines are analytical predictions based on Eq (2) in the main text. Cv=1 and Cp=1. Other parameter values: Bv=1.1 and Bv=2 (a); Bv=8 and Bv=2 (b); Bv=8 and Bv=8 (c). We use g1 = 4 and g2 = 4 in the theoretical calculations. (TIF) [file pcbi.1006947.s003.tif]
