# Supplementary material for: Evolutionary multiplayer games on graphs with edge diversity
Source: PLoS Comput Biol. 2019 Apr 1;15(4):e1006947. doi: 10.1371/journal.pcbi.1006947 (PMC6459562; doi:10.1371/journal.pcbi.1006947)
Supplement: S1 File — Calculations of fixation probabilities and structure coefficients for evolutionary multiplayer games on graphs with n types of edges in finite populations. Derivations of the replication equation for evolutionary multiplayer games on graphs with n types of edges in infinite populations. (PDF) [file pcbi.1006947.s004.pdf]

Supporting Methods: Evolutionary multiplayer games on graphs with edge diversity

Qi Su[1,2], Lei Zhou[1], Long Wang[1*]

1 Center for Systems and Control, College of Engineering, Peking University, Beijing, China

2 Center for Polymer Studies, Department of Physics, Boston University, Boston, Massachusetts, United States of America

* longwang@pku.edu.cn

1 Section 1. Fixation probability, structure coefficient, and replicator equation for evolutionary multiplayer games on graphs with n types of edges

We derive the analytical formulas based on the combination of pair approximation and diffusion theory. The method of pair approximation is formulated for infinite Cayley trees or Bethe lattices, which are regular graphs without any loops. For finite but sufficiently large random regular graphs ($N \gg k$), loops tend to be quite large, which has negligible impacts to validity of the pair approximation. Thus the obtained formulas approximate the simulated results.

1.1 Pair approximation

Let p_A and p_B be the frequencies of A-players and B-players in a population. Let p_{AA}, p_{AB}, p_{BA} and p_{BB} be the frequencies of AA, AB, BA and BB pairs. Let $q_{X|Y}$ be the conditional probability of finding an X-player given that the adjacent node is occupied by a Y-player, where X and Y are either A or B. Let G_i denote the subgraph consisting of all nodes and all edges of type i. We distinguish aforementioned variables associated with G_i by labelling (G_i), such as $p_{AA}^{(G_1)}$ the frequencies of AA pairs in G_1 and $p_{AA}^{(G_2)}$ in G_2. $p_A^{(G_i)}$ is identical to $p_A^{(G_j)}$ for any pairs i, j, and thus we simplify them as p_A. In the random regular graph with n types of edges, the degree of each node in G_i is identical (see the model in the main text) and thus we treat G_i as random regular graph. We have identities

$$p_A + p_B = 1, \tag{1}$$

$$p_{AB}^{(G_i)} = p_{BA}^{(G_i)}, \tag{2}$$

$$q_{X|Y}^{(G_i)} = \frac{p_{XY}^{(G_i)}}{p_Y}, \tag{3}$$

$$q_{A|Y}^{(G_i)} + q_{B|Y}^{(G_i)} = 1, \tag{4}$$

for any $1 \le i \le n$. Eqs (1-4) imply that the whole system can be described by $n+1$ variables, i.e. p_A and $q^{(G_i)}_{A|A}$ where $1 \le i \le n$. These notations are given by

$$\begin{aligned}
p_B &= 1 - p_A, \\
p^{(G_i)}_{AA} &= p_A q^{(G_i)}_{A|A}, \\
p^{(G_i)}_{AB} = p^{(G_i)}_{BA} &= p_A(1 - q^{(G_i)}_{A|A}), \\
p^{(G_i)}_{BB} &= 1 - 2p_A + p_A q^{(G_i)}_{A|A}, \\
q^{(G_i)}_{B|A} &= 1 - q^{(G_i)}_{A|A}, \\
q^{(G_i)}_{B|B} &= \frac{1 - 2p_A + p_A q^{(G_i)}_{A|A}}{1 - p_A}, \\
q^{(G_i)}_{A|B} &= \frac{p_A(1 - q^{(G_i)}_{A|A})}{1 - p_A}.
\end{aligned}$$

Let g_i denote the node degree in G_i. The node degree for the entire network is $k = \sum_{i=1}^{n} g_i$. Let $a_{s_1 \cdots s_n}$ be the payoff of an $A-$player that has s_i neighboring $A-$players and $g_i - s_i$ neighboring $B-$players in G_i $(1 \le i \le n)$. Let $b_{s_1 \cdots s_n}$ be the payoff of a $B-$player that has s_i neighboring $A-$players and $g_i - s_i$ neighboring $B-$players in G_i $(1 \le i \le n)$. Each individual is assigned a payoff by a single interaction with all neighbors. Then the payoff is transformed to the fitness for the evolution of system. Here the population evolves based on the Moran death-birth process [1]. In each generation, a random individual is selected to die. All neighbors compete to occupy the empty site proportional to their fitness. Other processes can be investigated analogously. In the following, we calculate the expected change of p_A and $p^{(G_i)}_{AA}$ in each step.

1.2 Updating a B-player

A B-player is selected to die with probability p_B. Its k neighbors compete to take over the vacant node. Let $k^{(G_i)}_A$ and $k^{(G_i)}_B$ denote the number of A- and B-players among g_i neighbors in G_i $(1 \le i \le n)$. We have $k^{(G_i)}_A + k^{(G_i)}_B = g_i$. The probability for such a neighborhood configuration is

$$\mathcal{B}^{g_1 \cdots g_i \cdots g_n}_{k^{(G_1)}_A \cdots k^{(G_i)}_A \cdots k^{(G_n)}_A} = \prod_{i=1}^{n} \binom{g_i}{k^{(G_i)}_A} (q^{(G_i)}_{A|B})^{k^{(G_i)}_A} (q^{(G_i)}_{B|B})^{k^{(G_i)}_B}. \tag{5}$$

Then the average fitness of an A-player connected to this dead B-player by an edge in G_i and that of a B-player are respectively given by

$$F_{A|B}^{(G_i)} = 1 - \omega + \omega \pi_{A|B}^{(G_i)},$$
$$F_{B|B}^{(G_i)} = 1 - \omega + \omega \pi_{B|B}^{(G_i)},$$

where

$$\pi_{A|B}^{(G_i)} = \sum_{s_1=0}^{g_1} \cdots \sum_{s_i=0}^{g_i-1} \cdots \sum_{s_n=0}^{g_n} \left[\prod_{j=1}^{n} \binom{g_j - \delta_{i,j}}{s_j} \left(q_{A|A}^{(G_j)}\right)^{s_j} \left(q_{B|A}^{(G_j)}\right)^{g_j-\delta_{i,j}-s_j} \right] a_{s_1 \cdots s_i \cdots s_n} \tag{6}$$

and

$$\pi_{B|B}^{(G_i)} = \sum_{s_1=0}^{g_1} \cdots \sum_{s_i=0}^{g_i-1} \cdots \sum_{s_n=0}^{g_n} \left[\prod_{j=1}^{n} \binom{g_j - \delta_{i,j}}{s_j} \left(q_{A|B}^{(G_j)}\right)^{s_j} \left(q_{B|B}^{(G_j)}\right)^{g_j-\delta_{i,j}-s_j} \right] b_{s_1 \cdots s_i \cdots s_n} \tag{7}$$

represent the expected payoffs from interactions with $\sum_{i=1}^{n} g_i$ neighbors. $\delta_{i,j} = 1$ if $j = i$ and $\delta_{i,j} = 0$ if $j \neq i$. The parameter ω denotes the intensity of selection and $w \ll 1$ means that the payoff from the game just contributes a little to one's fitness. Here we consider the weak selection.

The probability that an A-player takes over the empty site is given by

$$\begin{aligned}
&\frac{\sum_{i=1}^{n} k_A^{(G_i)} F_{A|B}^{(G_i)}}{\sum_{i=1}^{n} k_A^{(G_i)} F_{A|B}^{(G_i)} + \sum_{i=1}^{n} k_B^{(G_i)} F_{B|B}^{(G_i)}} \\
=&\frac{\sum_{i=1}^{n} k_A^{(G_i)}}{k} + \frac{\omega}{k^2} \sum_{i=1}^{n} \sum_{j=1}^{n} k_A^{(G_i)} k_B^{(G_j)} \left(\pi_{A|B}^{(G_i)} - \pi_{B|B}^{(G_j)}\right) + O(\omega^2).
\end{aligned} \tag{8}$$

Therefore, combining Eqs (5,8), p_A increases by $\frac{1}{N}$ with probability

$$\begin{aligned}
\text{Prob}\left(\Delta p_A = \frac{1}{N}\right) =& p_A \sum_{k_A^{(G_1)}=0}^{g_1} \cdots \sum_{k_A^{(G_i)}=0}^{g_i} \cdots \sum_{k_A^{(G_n)}=0}^{g_n} \mathcal{B}_{k_A^{(G_1)} \cdots k_A^{(G_i)} \cdots k_A^{(G_n)}}^{g_1 \cdots g_i \cdots g_n} \\
& \frac{\sum_{i=1}^{n} k_A^{(G_i)} F_{A|B}^{(G_i)}}{\sum_{i=1}^{n} k_A^{(G_i)} F_{A|B}^{(G_i)} + \sum_{i=1}^{n} k_B^{(G_i)} F_{B|B}^{(G_i)}} \\
=& \frac{p_B}{k} \sum_{i=1}^{n} g_i q_{A|B}^{(G_i)} + \frac{\omega p_B}{k^2} \Gamma_B + O(\omega^2),
\end{aligned}$$

where

$$\Gamma_B = \sum_{i=1}^{n} \sum_{j=1}^{n} g_i g_j q_{A|B}^{(G_i)} q_{B|B}^{(G_j)} \left(\pi_{A|B}^{(G_i)} - \pi_{B|B}^{(G_j)}\right) - \sum_{i=1}^{n} g_i q_{A|B}^{(G_i)} q_{B|B}^{(G_i)} \left(\pi_{A|B}^{(G_i)} - \pi_{B|B}^{(G_i)}\right). \tag{9}$$

Regarding pairs, if an A-player in G_i occupies the vacant site then the number of AA pairs in G_i increases by $k_A^{(G_i)}$. Given that the total number of pairs in G_i is $g_iN/2$, the probability that $p_{AA}^{(G_i)}$ increases by $2k_A^{(G_i)}/(g_iN)$ is given by

$$\text{Prob}\left(\Delta p_{AA}^{(G_i)} = \frac{2k_A^{(G_i)}}{g_iN}\right) = p_B \sum_{k_A^{(G_1)}=0}^{g_1} \cdots \sum_{k_A^{(G_{i-1})}=0}^{g_{i-1}} \sum_{k_A^{(G_{i+1})}=0}^{g_{i+1}} \cdots \sum_{k_A^{(G_n)}=0}^{g_n} \mathcal{B}^{g_1\cdots g_i\cdots g_n}_{k_A^{(G_1)}\cdots k_A^{(G_i)}\cdots k_A^{(G_n)}} \frac{\sum_{j=1}^n k_A^{(G_j)} F_{A|B}^{(G_j)}}{\sum_{j=1}^n k_A^{(G_j)} F_{A|B}^{(G_j)} + \sum_{j=1}^n k_B^{(G_j)} F_{B|B}^{(G_j)}}.$$

1.3 Updating an A-player

An A-player is selected to die with probability p_A. All k individuals, i.e., $k_A^{(G_i)}$ A-players and $k_B^{(G_i)}$ B-players in G_i $(1 \leq i \leq n)$, compete to occupy the empty site. The probability for such a neighborhood configuration is given by

$$\mathcal{A}^{g_1\cdots g_i\cdots g_n}_{k_A^{(G_1)}\cdots k_A^{(G_i)}\cdots k_A^{(G_n)}} = \prod_{i=1}^n \binom{g_i}{k_A^{(G_i)}} (q_{A|A}^{(G_i)})^{k_A^{(G_i)}} (q_{B|A}^{(G_i)})^{k_B^{(G_i)}}. \tag{10}$$

Then the average fitness of each A-player and each B-player connected to this dead A-player by an edge in G_i are respectively given by

$$F_{A|A}^{(G_i)} = 1 - \omega + \omega \pi_{A|A}^{(G_i)},$$
$$F_{B|A}^{(G_i)} = 1 - \omega + \omega \pi_{B|A}^{(G_i)},$$

where

$$\pi_{A|A}^{(G_i)} = \sum_{s_1=0}^{g_1} \cdots \sum_{s_i=0}^{g_i-1} \cdots \sum_{s_n=0}^{g_n} \left[\prod_{j=1}^n \binom{g_j - \delta_{i,j}}{s_j} \left(q_{A|A}^{(G_j)}\right)^{s_j} \left(q_{B|A}^{(G_j)}\right)^{g_j-\delta_{i,j}-s_j}\right] a_{s_1\cdots(s_i+1)\cdots s_n} \tag{11}$$

and

$$\pi_{B|A}^{(G_i)} = \sum_{s_1=0}^{g_1} \cdots \sum_{s_i=0}^{g_i-1} \cdots \sum_{s_n=0}^{g_n} \left[\prod_{j=1}^n \binom{g_j - \delta_{i,j}}{s_j} \left(q_{A|B}^{(G_j)}\right)^{s_j} \left(q_{B|B}^{(G_j)}\right)^{g_j-\delta_{i,j}-s_j}\right] b_{s_1\cdots(s_i+1)\cdots s_n} \tag{12}$$

represent the expected payoffs from interactions with $\sum_{i=1}^n g_i$ neighbors.

The probability that a B-player takes over the empty site with probability

$$\begin{aligned}&\frac{\sum_{i=1}^{n} k_B^{(G_i)} F_{B|A}^{(G_i)}}{\sum_{i=1}^{n} k_A^{(G_i)} F_{A|A}^{(G_i)} + \sum_{i=1}^{n} k_B^{(G_i)} F_{B|A}^{(G_i)}} \\ =&\frac{\sum_{i=1}^{n} k_B^{(G_i)}}{k} + \frac{\omega}{k^2} \sum_{i=1}^{n} \sum_{j=1}^{n} k_A^{(G_i)} k_B^{(G_j)} \left(\pi_{B|A}^{(G_j)} - \pi_{A|A}^{(G_i)} \right) + O(\omega^2).\end{aligned} \tag{13}$$

Therefore, combining Eqs (10) and (13), p_A decreases by $\frac{1}{N}$ with probability

$$\begin{aligned}\text{Prob}\left(\Delta p_A = -\frac{1}{N} \right) =& p_A \sum_{k_A^{(G_1)}=0}^{g_1} \cdots \sum_{k_A^{(G_i)}=0}^{g_i} \cdots \sum_{k_A^{(G_n)}=0}^{g_n} \mathcal{A}^{g_1 \cdots g_i \cdots g_n}_{k_A^{(G_1)} \cdots k_A^{(G_i)} \cdots k_A^{(G_n)}} \\ & \frac{\sum_{i=1}^{n} k_B^{(G_i)} F_{B|A}^{(G_i)}}{\sum_{i=1}^{n} k_A^{(G_i)} F_{A|A}^{(G_i)} + \sum_{i=1}^{n} k_B^{(G_i)} F_{B|A}^{(G_i)}} \\ =& \frac{p_A}{k} \sum_{i=1}^{n} g_i q_{B|A}^{(G_i)} + \frac{\omega p_A}{k^2} \Gamma_A + O(\omega^2),\end{aligned}$$

where

$$\Gamma_A = \sum_{i=1}^{n} \sum_{j=1}^{n} g_i g_j q_{A|A}^{(G_i)} q_{B|A}^{(G_j)} \left(\pi_{B|A}^{(G_j)} - \pi_{A|A}^{(G_i)} \right) - \sum_{i=1}^{n} g_i q_{A|A}^{(G_i)} q_{B|A}^{(G_i)} \left(\pi_{B|A}^{(G_i)} - \pi_{A|A}^{(G_i)} \right). \tag{14}$$

Regarding pairs, if a B-player in G_i occupies the vacant site then the number of AA pairs in G_i decreases by $k_A^{(G_i)}$ and therefore $p_{AA}^{(G_i)}$ decreases by $2k_A^{(G_i)}/(g_i N)$ with probability

$$\begin{aligned}\text{Prob}\left(\Delta p_{AA}^{(G_i)} = -\frac{2k_A^{(G_i)}}{g_i N} \right) =& p_A \sum_{k_A^{(G_1)}=0}^{g_1} \cdots \sum_{k_A^{(G_{i-1})}=0}^{g_{i-1}} \sum_{k_A^{(G_{i+1})}=0}^{g_{i+1}} \cdots \sum_{k_A^{(G_n)}=0}^{g_n} \mathcal{A}^{g_1 \cdots g_i \cdots g_n}_{k_A^{(G_1)} \cdots k_A^{(G_i)} \cdots k_A^{(G_n)}} \\ & \frac{\sum_{j=1}^{n} k_B^{(G_j)} F_{B|A}^{(G_j)}}{\sum_{j=1}^{n} k_A^{(G_j)} F_{A|A}^{(G_j)} + \sum_{j=1}^{n} k_B^{(G_j)} F_{B|A}^{(G_j)}}.\end{aligned}$$

1.4 Different time scales

Supposing that one replacement event takes place in one unit of time, we can get the time derivatives of p_A and $p_{AA}^{(G_i)}$, given by

$$\begin{aligned}\dot{p}_A =& \frac{1}{N} \cdot \text{Prob}\left(\Delta p_A = \frac{1}{N} \right) + \left(-\frac{1}{N} \right) \cdot \text{Prob}\left(\Delta p_A = -\frac{1}{N} \right) \\ =& \frac{\omega}{N k^2} \left(p_B \Gamma_B - p_A \Gamma_A \right) + O(\omega^2),\end{aligned} \tag{15}$$

$$
\begin{aligned}
\dot{p}_{AA}^{(G_i)} &= \sum_{k_A^{(G_i)}=0}^{g_i} \frac{2k_A^{(G_i)}}{g_i N} \cdot \text{Prob}\left(\Delta p_{AA}^{(G_i)} = \frac{2k_A^{(G_i)}}{g_i N}\right) + \sum_{k_A^{(G_i)}=0}^{g_i} \left(-\frac{2k_A^{(G_i)}}{g_i N}\right) \cdot \text{Prob}\left(\Delta p_{AA}^{(G_i)} = -\frac{2k_A^{(G_i)}}{g_i N}\right) \\
&= \frac{2p_A}{Nk(1-p_A)} \left[\sum_{j=1}^{n} g_j \left(1 - q_{A|A}^{(G_j)}\right)\left(p_A - q_{A|A}^{(G_i)}\right) + \left(1 - q_{A|A}^{(G_i)}\right)\left(1 + q_{A|A}^{(G_i)} - 2p_A\right)\right] + O(\omega).
\end{aligned}
\tag{16}
$$

From Eqs (15) and (16), we have

$$
\begin{aligned}
\dot{q}_{A|A}^{(G_i)} &= \frac{\mathrm{d}}{\mathrm{d}t}\left(\frac{p_{AA}^{(G_i)}}{p_A}\right) \\
&= \frac{2p_A}{Nk(1-p_A)} \left[\sum_{j=1}^{n} g_j \left(1 - q_{A|A}^{(G_j)}\right)\left(p_A - q_{A|A}^{(G_i)}\right) + \left(1 - q_{A|A}^{(G_i)}\right)\left(1 + q_{A|A}^{(G_i)} - 2p_A\right)\right] + O(\omega).
\end{aligned}
\tag{17}
$$

First, we need to clarify that by definition, the pair approximation method only describes the transient behavior of the system, i.e., when $0 < p_A < 1$; otherwise, some conditional probabilities are undefined. The two other special cases where $p_A = 0$ (the extinction of A-players) and $p_A = 1$ (the fixation of A-players) are the two absorbing states of the evolutionary process, which is beyond the scope of the pair approximation.

Rewriting Eqs (15) and (17) as a function of p_A and $q_{A|A}^{(G_i)}$, we have

$$
\begin{aligned}
\dot{p}_A &= \omega \cdot \Psi_0(p_A, q_{A|A}^{(G_1)}, \cdots, q_{A|A}^{(G_n)}) + O(\omega^2), \\
\dot{q}_{A|A}^{(G_i)} &= \Psi_i(p_A, q_{A|A}^{(G_1)}, \cdots, q_{A|A}^{(G_n)}) + O(\omega).
\end{aligned}
$$

For weak selection ($\omega \ll 1$), $q_{A|A}^{(G_i)}$ equilibrates much more quickly than p_A. Thus, this dynamical system converges rapidly onto the slow manifold defined by $\Psi_i(p_A, q_{A|A}^{(G_1)}, \cdots, q_{A|A}^{(G_n)}) = 0$ $(1 \leq i \leq n)$ (refer to chapter 11 in Ref [2]).

Next we will prove that for $0 < p_A < 1$ the system will converge to the unique slow manifold given by

$$
(q_{A|A}^{(G_1)}, q_{A|A}^{(G_2)}, \cdots, q_{A|A}^{(G_n)}) = (\frac{k-2}{k-1}p_A + \frac{1}{k-1}, \frac{k-2}{k-1}p_A + \frac{1}{k-1}, \cdots, \frac{k-2}{k-1}p_A + \frac{1}{k-1}). \tag{18}
$$

The system of equations $\Psi_i(p_A, q_{A|A}^{(G_1)}, \cdots, q_{A|A}^{(G_n)}) = 0$ $(1 \leq i \leq n)$ can be reduced to the following

equations

$$\sum_{l=1}^{n} g_l \left(1 - q_{A|A}^{(G_l)}\right)\left(p_A - q_{A|A}^{(G_i)}\right) + \left(1 - q_{A|A}^{(G_i)}\right)\left(1 + q_{A|A}^{(G_i)} - 2p_A\right) = 0 \tag{19}$$

for any i. Since $q_{A|A}^{(G_i)}$ represents the conditional probability of finding an A-player given that the adjacent node connected by an edge of type i is occupied by an A-player, we have $0 \leq q_{A|A}^{(G_i)} \leq 1$. A special case is that $q_{A|A}^{(G_i)} = 1$ for all $1 \leq i \leq n$. This leads to that the probability of finding an A-player given that the adjacent node is occupied by an A-player is $\sum_{i=1}^{n} \frac{g_i}{k} q_{A|A}^{(G_i)} = 1$, which implies that the whole population consists of only A-players, i.e., $p_A = 1$. Meanwhile, $p_A = 1$ implies that $q_{A|A}^{(G_i)} = 1$ for all $1 \leq i \leq n$. Therefore, for $0 < p_A < 1$, there exists at least one i such that $q_{A|A}^{(G_i)} < 1$. Moreover, if for some i_1, $p_A = q_{A|A}^{(G_{i_1})}$, Eq (19) implies that $\left(1 - q_{A|A}^{(G_{i_1})}\right)(1 - p_A) = (1 - p_A)^2 = 0$, which is impossible. This indicates that $p_A \neq q_{A|A}^{(G_i)}$ for any i.

We first consider the simplest case where there is one type of edges in the population. In this case, $n = 1$ and Eq (19) becomes

$$k \left(1 - q_{A|A}^{(G_1)}\right)\left(p_A - q_{A|A}^{(G_1)}\right) + \left(1 - q_{A|A}^{(G_1)}\right)\left(1 + q_{A|A}^{(G_1)} - 2p_A\right) = 0.$$

Since $q_{A|A}^{(G_1)} < 1$, we have $q_{A|A}^{(G_1)} = \frac{k-2}{k-1} p_A + \frac{1}{k-1}$.

For $2 \leq n \leq k$, to solve Eq (19), we divide Eq (19) by $p_A - q_{A|A}^{(G_i)}$ and rearrange relevant terms. Then we have

$$\frac{\left(1 - q_{A|A}^{(G_i)}\right)\left(1 + q_{A|A}^{(G_i)} - 2p_A\right)}{p_A - q_{A|A}^{(G_i)}} = -\sum_{l=1}^{n} g_l \left(1 - q_{A|A}^{(G_l)}\right) < 0, \tag{20}$$

where the inequality comes from the fact that there is at least one $q_{A|A}^{(G_l)} < 1$. This inequality further leads to $q_{A|A}^{(G_i)} < 1$ for all i. Meanwhile, from the above equation, we get

$$\frac{\left(1 - q_{A|A}^{(G_i)}\right)\left(1 + q_{A|A}^{(G_i)} - 2p_A\right)}{p_A - q_{A|A}^{(G_i)}} = \frac{\left(1 - q_{A|A}^{(G_j)}\right)\left(1 + q_{A|A}^{(G_j)} - 2p_A\right)}{p_A - q_{A|A}^{(G_j)}} \tag{21}$$

for any i and j. For $i = j$, Eq (21) always holds. For $i \neq j$, simplifying Eq (21), we have

$$\left(q_{A|A}^{(G_i)} - q_{A|A}^{(G_j)}\right)\left[\left(p_A - q_{A|A}^{(G_i)}\right)\left(p_A - q_{A|A}^{(G_j)}\right) + (1 - p_A)^2\right] = 0. \tag{22}$$

Equation (22) suggests that for any $i \neq j$ and $0 < p_A < 1$, either $q_{A|A}^{(G_i)} - q_{A|A}^{(G_j)} = 0$ or $\left(p_A - q_{A|A}^{(G_i)}\right)\left(p_A - q_{A|A}^{(G_j)}\right) + (1-p_A)^2 = 0$.

Let us assume that there exists at least one pair (i, j) $(i \neq j)$, which satisfies $\left(p_A - q_{A|A}^{(G_i)}\right)\left(p_A - q_{A|A}^{(G_j)}\right) + (1-p_A)^2 = 0$. Since $(1-p_A)^2 > 0$ for any $0 < p_A < 1$, we have $\left(p_A - q_{A|A}^{(G_i)}\right)\left(p_A - q_{A|A}^{(G_j)}\right) < 0$. This leads to either $q_{A|A}^{(G_i)} > p_A > q_{A|A}^{(G_j)}$ or $q_{A|A}^{(G_i)} < p_A < q_{A|A}^{(G_j)}$. Therefore, for $1 \leq i \leq n$, $q_{A|A}^{(G_i)}$ must have a maximum and a minimum. Denote this maximum as $q_{A|A}^{\max}$ and the minimum as $q_{A|A}^{\min}$. From the above analysis, we have $q_{A|A}^{\max} > q_{A|A}^{\min}$.

Meanwhile, from Eq (20), we have

$$-\sum_{l=1}^{n} g_l \left(1 - q_{A|A}^{(G_l)}\right) = \frac{\left(1 - q_{A|A}^{(G_i)}\right)\left(1 + q_{A|A}^{(G_i)} - 2p_A\right)}{p_A - q_{A|A}^{(G_i)}} = \frac{\left(1 - q_{A|A}^{(G_i)}\right)\left(1 - p_A - \left(p_A - q_{A|A}^{(G_i)}\right)\right)\left(p_A - q_{A|A}^{(G_j)}\right)}{\left(p_A - q_{A|A}^{(G_i)}\right)\left(p_A - q_{A|A}^{(G_j)}\right)},$$

inserting $\left(p_A - q_{A|A}^{(G_i)}\right)\left(p_A - q_{A|A}^{(G_j)}\right) = -(1-p_A)^2$ into the above equation, we have

$$\frac{\left(1 - q_{A|A}^{(G_i)}\right)\left[(1-p_A)\left(p_A - q_{A|A}^{(G_j)}\right) + (1-p_A)^2\right]}{-(1-p_A)^2} = -\left(1 - q_{A|A}^{(G_i)}\right)\left(\frac{p_A - q_{A|A}^{(G_j)}}{1-p_A} + 1\right)$$

$$= -\left(1 - q_{A|A}^{(G_i)}\right)\frac{1 - q_{A|A}^{(G_j)}}{1-p_A} = -\sum_{l=1}^{n} g_l \left(1 - q_{A|A}^{(G_l)}\right),$$

which leads to

$$\frac{\left(1 - q_{A|A}^{(G_i)}\right)\left(1 - q_{A|A}^{(G_j)}\right)}{1-p_A} = \sum_{l=1}^{n} g_l \left(1 - q_{A|A}^{(G_l)}\right)$$

Since $q_{A|A}^{\max} \neq q_{A|A}^{\min}$, they must satisfy the above equation, which reads

$$\frac{\left(1 - q_{A|A}^{\max}\right)\left(1 - q_{A|A}^{\min}\right)}{1-p_A} = \sum_{l=1}^{n} g_l \left(1 - q_{A|A}^{(G_l)}\right).$$

and also $q_{A|A}^{\max} > p_A > q_{A|A}^{\min}$. Now we have

$$\sum_{l=1}^{n} g_l \left(1 - q_{A|A}^{(G_l)}\right) = \frac{\left(1 - q_{A|A}^{\max}\right)\left(1 - q_{A|A}^{\min}\right)}{1-p_A} < 1 - q_{A|A}^{\min}.$$

Since $g_l \geq 1$ and $q_{A|A}^{(G_l)} < 1$ for all l, $\sum_{l=1}^{n} g_l \left(1 - q_{A|A}^{(G_l)}\right) \geq 1 - q_{A|A}^{\min}$, which contradicts with $\sum_{l=1}^{n} g_l \left(1 - q_{A|A}^{(G_l)}\right) < 1 - q_{A|A}^{\min}$.

Therefore, any pair (i,j) which satisfies $\left(p_A - q_{A|A}^{(G_i)}\right)\left(p_A - q_{A|A}^{(G_j)}\right) + (1-p_A)^2 = 0$ will leads to a contradiction. This means that for all i, j, $q_{A|A}^{(G_i)} = q_{A|A}^{(G_j)}$. Inserting this into Eq (19), we have that the $q_{A|A}^{(G_i)} = \frac{k-2}{k-1}p_A + \frac{1}{k-1}$, which coincides with the results for $n = 1$.

In summary, our above analysis proves that the system will rapidly converge to the unique slow manifold described by $(q_{A|A}^{(G_1)}, q_{A|A}^{(G_2)}, \cdots, q_{A|A}^{(G_n)}) = (\frac{k-2}{k-1}p_A + \frac{1}{k-1}, \frac{k-2}{k-1}p_A + \frac{1}{k-1}, \cdots, \frac{k-2}{k-1}p_A + \frac{1}{k-1})$.

Defining $z = \frac{1}{k-1}$ and using $q_{A|A}^{(G_i)} = \frac{k-2}{k-1}p_A + \frac{1}{k-1}$, we rewrite $q_{X|Y}^{(G_i)}$ as

$$q_{A|A}^{(G_i)} = p_A + z(1-p_A), \tag{23}$$

$$q_{B|A}^{(G_i)} = (1-z)(1-p_A), \tag{24}$$

$$q_{A|B}^{(G_i)} = (1-z)p_A, \tag{25}$$

$$q_{B|B}^{(G_i)} = zp_A + (1-p_A). \tag{26}$$

1.5 Diffusion approximation

Here we can describe the trajectory of p_A as a one dimensional diffusion process, where the drift coefficient corresponds the mean of Δp_A (the change in p_A in each generation) and the diffusion coefficient corresponds to the variance of Δp_A. Let $m(p_A)$ and $v(p_A)$ denote the mean and variance of Δp_A, respectively. We use the Kolmogorov/Fokker-Planck backward equation to study such a diffusion process. The fixation probability of A-players, $\phi_A(x)$ with initial frequency $p_A(t=0) = x$ satisfies the differential equation [see Eq (5.2.186) in Ref [3] and detailed derivation therein]:

$$m(x)\frac{d\phi_A(x)}{dx} + \frac{v(x)}{2}\frac{d^2\phi_A(x)}{dx^2} = 0 \tag{27}$$

with two boundary conditions $\phi_A(0) = 0$ and $\phi_A(1) = 1$. The solution for the above differential equation with boundary conditions is [see Eq (5.2.189) in Ref [3]]:

$$\phi_A(x) = \frac{\int_0^x \psi(y)dy}{\int_0^1 \psi(y)dy}, \tag{28}$$

where

$$\psi(y) = \exp\left(-\int^y \frac{2m(r)}{v(r)}dr\right). \tag{29}$$

Within a short time interval, Δt, we have

$$\begin{aligned} m(p_A) &= \frac{\mathrm{E}(\Delta p_A)}{\Delta t} \\ &= \frac{1}{N} \cdot \mathrm{Prob}\left(\Delta p_A = \frac{1}{N}\right) + \left(-\frac{1}{N}\right) \cdot \mathrm{Prob}\left(\Delta p_A = -\frac{1}{N}\right) \\ &\approx \frac{\omega}{Nk^2}\left(p_B\Gamma_B - p_A\Gamma_A\right) \end{aligned} \tag{30}$$

and

$$\begin{aligned} v(p_A) &= \frac{\mathrm{V}(\Delta p_A)}{\Delta t} \\ &= \frac{1}{\Delta t}\mathrm{E}\left[\left(\Delta p_A - \frac{\mathrm{E}\left[\Delta p_A\right]}{\Delta t}\right)^2\right]\Delta t \\ &= \left(\frac{1}{N} - \frac{\mathrm{E}\left[\Delta p_A\right]}{\Delta t}\right)^2 \cdot \mathrm{Prob}\left(\Delta p_A = \frac{1}{N}\right) \\ &\quad + \left(-\frac{1}{N} - \frac{\mathrm{E}\left[\Delta p_A\right]}{\Delta t}\right)^2 \cdot \mathrm{Prob}\left(\Delta p_A = -\frac{1}{N}\right) \\ &\quad + \left(0 - \frac{\mathrm{E}\left[\Delta p_A\right]}{\Delta t}\right)^2 \cdot \left[1 - \mathrm{Prob}\left(\Delta p_A = \frac{1}{N}\right) - \mathrm{Prob}\left(\Delta p_A = -\frac{1}{N}\right)\right] \\ &= \frac{1}{N^2} \cdot \mathrm{Prob}\left(\Delta p_A = \frac{1}{N}\right) + \frac{1}{N^2} \cdot \mathrm{Prob}\left(\Delta p_A = -\frac{1}{N}\right) + O(\omega) \\ &\approx \frac{2(k-2)p_A(1-p_A)}{N^2(k-1)}. \end{aligned} \tag{31}$$

According to Eqs (6,7,9,11,12,14,23-26), $p_B\Gamma_B - p_A\Gamma_A$ in Eq (30) is actually a polynomial in p_A. We here can write it in a form only containing variable p_A. To achieve this, we make full use of Eqs (19-22) in Supplemental Methods of Ref [4] and Appendix B of Ref [5] to obtain the following

identities:

$$\sum_{s_1=0}^{g_1}\cdots\sum_{s_i=0}^{g_i}\cdots\sum_{s_n=0}^{g_n}\left[\prod_{j=1}^{n}\binom{g_j}{s_j}\left[x+z(1-x)\right]^{s_j}\left[(1-z)(1-x)\right]^{g_j-s_j}\right]a_{s_1\cdots s_i\cdots s_n}$$
$$=\sum_{s_1=0}^{g_1}\cdots\sum_{s_i=0}^{g_i}\cdots\sum_{s_n=0}^{g_n}\left[\prod_{j=1}^{n}\binom{g_j}{s_j}x^{s_j}(1-x)^{g_j-s_j}\right]$$
$$\sum_{r_1=0}^{g_1-s_1}\cdots\sum_{r_i=0}^{g_i-s_i}\cdots\sum_{r_n=0}^{g_n-s_n}\left[\prod_{j=1}^{n}\binom{g_j-s_j}{r_j}z^{r_j}(1-z)^{g_j-s_j-r_j}\right]a_{(s_1+r_1)\cdots(s_i+r_i)\cdots(s_n+r_n)},\tag{32}$$

$$\sum_{s_1=0}^{g_1}\cdots\sum_{s_i=0}^{g_i}\cdots\sum_{s_n=0}^{g_n}\left[\prod_{j=1}^{n}\binom{g_j}{s_j}\left[(1-z)x\right]^{s_j}\left[zx+1-x\right]^{g_j-s_j}\right]a_{s_1\cdots s_i\cdots s_n}$$
$$=\sum_{s_1=0}^{g_1}\cdots\sum_{s_i=0}^{g_i}\cdots\sum_{s_n=0}^{g_n}\left[\prod_{j=1}^{n}\binom{g_j}{s_j}x^{s_j}(1-x)^{g_j-s_j}\right]$$
$$\sum_{r_1=0}^{s_1}\cdots\sum_{r_i=0}^{s_i}\cdots\sum_{r_n=0}^{s_n}\left[\prod_{j=1}^{n}\binom{s_j}{r_j}z^{r_j}(1-z)^{s_j-r_j}\right]a_{(s_1-r_1)\cdots(s_i-r_i)\cdots(s_n-r_n)},\tag{33}$$

$$x\sum_{s_1=0}^{g_1}\cdots\sum_{s_i=0}^{g_i-1}\cdots\sum_{s_n=0}^{g_n}\left[\prod_{j=1}^{n}\binom{g_j-\delta_{i,j}}{s_j}x^{s_j}(1-x)^{g_j-\delta_{i,j}-s_j}\right]a_{s_1\cdots s_i\cdots s_n}$$
$$=\sum_{s_1=0}^{g_1}\cdots\sum_{s_i=0}^{g_i}\cdots\sum_{s_n=0}^{g_n}\left[\prod_{j=1}^{n}\binom{g_j}{s_j}x^{s_j}(1-x)^{g_j-s_j}\right]\frac{s_i a_{s_1\cdots(s_i-1)\cdots s_n}}{g_i},\tag{34}$$

$$(1-x)\sum_{s_1=0}^{g_1}\cdots\sum_{s_i=0}^{g_i-1}\cdots\sum_{s_n=0}^{g_n}\left[\prod_{j=1}^{n}\binom{g_j-\delta_{i,j}}{s_j}x^{s_j}(1-x)^{g_j-\delta_{i,j}-s_j}\right]a_{s_1\cdots s_i\cdots s_n}$$
$$=\sum_{s_1=0}^{g_1}\cdots\sum_{s_i=0}^{g_i}\cdots\sum_{s_n=0}^{g_n}\left[\prod_{j=1}^{n}\binom{g_j}{s_j}x^{s_j}(1-x)^{g_j-s_j}\right]\frac{(g_i-s_i)a_{s_1\cdots s_i\cdots s_n}}{g_i}.\tag{35}$$

Here we provide detailed proof of Eqs (32) and (34). From Eq (19) in Supplemental Methods of Ref [4], we have

$$\sum_{s_j=0}^{g_j}\binom{g_j}{s_j}\left[x+z(1-x)\right]^{s_j}\left[(1-z)(1-x)\right]^{g_j-s_j}a_{s_1\cdots s_j\cdots s_n}$$
$$=\sum_{s_j=0}^{g_j}\binom{g_j}{s_j}x^{s_j}(1-x)^{g_j-s_j}\sum_{r_j=0}^{g_j-s_j}\binom{g_j-s_j}{r_j}z^{r_j}(1-z)^{g_j-s_j-r_j}a_{s_1\cdots(s_j+r_j)\cdots s_n}.$$

Repeating using this equation, we have

$$\sum_{s_1=0}^{g_1}\cdots\sum_{s_i=0}^{g_i}\cdots\sum_{s_n=0}^{g_n}\left[\prod_{j=1}^{n}\binom{g_j}{s_j}[x+z(1-x)]^{s_j}[(1-z)(1-x)]^{g_j-s_j}\right]a_{s_1\cdots s_i\cdots s_n}$$

$$=\sum_{s_1=0}^{g_1}\cdots\sum_{s_i=0}^{g_i}\cdots\sum_{s_{n-1}=0}^{g_{n-1}}\left[\prod_{j=1}^{n-1}\binom{g_j}{s_j}[x+z(1-x)]^{s_j}[(1-z)(1-x)]^{g_j-s_j}\right]$$

$$\left[\sum_{s_n=0}^{g_n}\binom{g_n}{s_n}[x+z(1-x)]^{s_n}[(1-z)(1-x)]^{g_n-s_n}a_{s_1\cdots s_i\cdots s_n}\right]$$

$$=\sum_{s_1=0}^{g_1}\cdots\sum_{s_i=0}^{g_i}\cdots\sum_{s_{n-1}=0}^{g_{n-1}}\left[\prod_{j=1}^{n-1}\binom{g_j}{s_j}[x+z(1-x)]^{s_j}[(1-z)(1-x)]^{g_j-s_j}\right]$$

$$\sum_{s_n=0}^{g_n}\binom{g_n}{s_n}x^{s_n}(1-x)^{g_n-s_n}\sum_{r_n=0}^{g_n-s_n}\binom{g_n-s_n}{r_n}z^{r_n}(1-z)^{g_n-s_n-r_n}a_{s_1\cdots s_i\cdots(s_n+r_n)}$$

$$=\sum_{s_1=0}^{g_1}\cdots\sum_{s_i=0}^{g_i}\cdots\sum_{s_{n-2}=0}^{g_{n-2}}\left[\prod_{j=1}^{n-2}\binom{g_j}{s_j}[x+z(1-x)]^{s_j}[(1-z)(1-x)]^{g_j-s_j}\right]$$

$$\sum_{s_n=0}^{g_n}\binom{g_n}{s_n}x^{s_n}(1-x)^{g_n-s_n}\sum_{r_n=0}^{g_n-s_n}\binom{g_n-s_n}{r_n}z^{r_n}(1-z)^{g_n-s_n-r_n}$$

$$\left[\sum_{s_{n-1}=0}^{g_{n-1}}\binom{g_{n-1}}{s_{n-1}}[x+z(1-x)]^{s_{n-1}}[(1-z)(1-x)]^{g_{n-1}-s_{n-1}}a_{s_1\cdots s_i\cdots s_{n-1}(s_n+r_n)}\right]$$

$$=\sum_{s_1=0}^{g_1}\cdots\sum_{s_i=0}^{g_i}\cdots\sum_{s_{n-2}=0}^{g_{n-2}}\left[\prod_{j=1}^{n-2}\binom{g_j}{s_j}[x+z(1-x)]^{s_j}[(1-z)(1-x)]^{g_j-s_j}\right]$$

$$\sum_{s_n=0}^{g_n}\binom{g_n}{s_n}x^{s_n}(1-x)^{g_n-s_n}\sum_{r_n=0}^{g_n-s_n}\binom{g_n-s_n}{r_n}z^{r_n}(1-z)^{g_n-s_n-r_n}$$

$$\sum_{s_{n-1}=0}^{g_{n-1}}\binom{g_{n-1}}{s_{n-1}}x^{s_{n-1}}(1-x)^{g_{n-1}-s_{n-1}}\sum_{r_{n-1}=0}^{g_{n-1}-s_{n-1}}\binom{g_{n-1}-s_{n-1}}{r_{n-1}}z^{r_{n-1}}(1-z)^{g_{n-1}-s_{n-1}-r_{n-1}}$$

$$a_{s_1\cdots s_i\cdots(s_{n-1}+r_{n-1})(s_n+r_n)}$$

$$=\cdots\cdots$$

$$=\sum_{s_1=0}^{g_1}\cdots\sum_{s_i=0}^{g_i}\cdots\sum_{s_n=0}^{g_n}\left[\prod_{j=1}^{n}\binom{g_j}{s_j}x^{s_j}(1-x)^{g_j-s_j}\right]$$

$$\sum_{r_1=0}^{g_1-s_1}\cdots\sum_{r_i=0}^{g_i-s_i}\cdots\sum_{r_n=0}^{g_n-s_n}\left[\prod_{j=1}^{n}\binom{g_j-s_j}{r_j}z^{r_j}(1-z)^{g_j-s_j-r_j}\right]a_{(s_1+r_1)\cdots(s_i+r_i)\cdots(s_n+r_n)}.$$

Then we prove Eq (34). From Eq (B4) in Ref [5], we have

$$x\sum_{s_i=0}^{g_i-1}\binom{g_i-1}{s_i}x^{s_i}(1-x)^{g_i-1-s_i}a_{s_1\cdots s_i\cdots s_n}=\sum_{s_i=0}^{g_i}\binom{g_i}{s_i}x^{s_i}(1-x)^{g_i-s_i}\frac{s_i a_{s_1\cdots(s_i-1)\cdots s_n}}{g_i}.$$

Using this equation, we can get

$$\begin{aligned}
&x\sum_{s_1=0}^{g_1}\cdots\sum_{s_i=0}^{g_i-1}\cdots\sum_{s_n=0}^{g_n}\left[\prod_{j=1}^{n}\binom{g_j-\delta_{i,j}}{s_j}x^{s_j}(1-x)^{g_j-\delta_{i,j}-s_j}\right]a_{s_1\cdots s_i\cdots s_n}\\
=&\sum_{s_1=0}^{g_1}\cdots\sum_{s_{i-1}=0}^{g_{i-1}}\sum_{s_{i+1}=0}^{g_{i+1}}\cdots\sum_{s_n=0}^{g_n}\left[\prod_{j\neq i}\binom{g_j}{s_j}x^{s_j}(1-x)^{g_j-s_j}\right]\\
&x\sum_{s_i=0}^{g_i-1}\binom{g_i-1}{s_i}x^{s_i}(1-x)^{g_i-1-s_i}a_{s_1\cdots s_i\cdots s_n}\\
=&\sum_{s_1=0}^{g_1}\cdots\sum_{s_{i-1}=0}^{g_{i-1}}\sum_{s_{i+1}=0}^{g_{i+1}}\cdots\sum_{s_n=0}^{g_n}\left[\prod_{j\neq i}\binom{g_j}{s_j}x^{s_j}(1-x)^{g_j-s_j}\right]\\
&\sum_{s_i=0}^{g_i}\binom{g_i}{s_i}x^{s_i}(1-x)^{g_i-s_i}\frac{s_i a_{s_1\cdots(s_i-1)\cdots s_n}}{g_i}\\
=&\sum_{s_1=0}^{g_1}\cdots\sum_{s_i=0}^{g_i}\cdots\sum_{s_n=0}^{g_n}\left[\prod_{j=1}^{n}\binom{g_j}{s_j}x^{s_j}(1-x)^{g_j-s_j}\right]\frac{s_i a_{s_1\cdots(s_i-1)\cdots s_n}}{g_i}.
\end{aligned}$$

Replacing Eqs (23-26) into Eqs (6,7,11,12) and applying Eqs (32) and (33), we have

$$\begin{aligned}
\pi_{A|B}^{(G_i)}=&\sum_{s_1=0}^{g_1}\cdots\sum_{s_i=0}^{g_i-1}\cdots\sum_{s_n=0}^{g_n}\left[\prod_{j=1}^{n}\binom{g_j-\delta_{i,j}}{s_j}p_A^{s_j}(1-p_A)^{g_j-\delta_{i,j}-s_j}\right]\\
&\sum_{r_1=0}^{g_1-s_1}\cdots\sum_{r_i=0}^{g_i-1-s_i}\cdots\sum_{r_n=0}^{g_n-s_n}\left[\prod_{j=1}^{n}\binom{g_j-\delta_{i,j}-s_j}{r_j}z^{r_j}(1-z)^{g_j-\delta_{i,j}-s_j-r_j}\right]a_{(s_1+r_1)\cdots(s_i+r_i)\cdots(s_n+r_n)},
\end{aligned}\tag{36}$$

$$\pi_{B|B}^{(G_i)} = \sum_{s_1=0}^{g_1} \cdots \sum_{s_i=0}^{g_i-1} \cdots \sum_{s_n=0}^{g_n} \left[\prod_{j=1}^{n} \binom{g_j - \delta_{i,j}}{s_j} p_A^{s_j} (1-p_A)^{g_j - \delta_{i,j} - s_j} \right]$$
$$\sum_{r_1=0}^{s_1} \cdots \sum_{r_i=0}^{s_i} \cdots \sum_{r_n=0}^{s_n} \left[\prod_{j=1}^{n} \binom{s_j}{r_j} z^{r_j} (1-z)^{s_j - r_j} \right] b_{(s_1-r_1)\cdots(s_i-r_i)\cdots(s_n-r_n)}, \tag{37}$$

$$\pi_{A|A}^{(G_i)} = \sum_{s_1=0}^{g_1} \cdots \sum_{s_i=0}^{g_i-1} \cdots \sum_{s_n=0}^{g_n} \left[\prod_{j=1}^{n} \binom{g_j - \delta_{i,j}}{s_j} p_A^{s_j} (1-p_A)^{g_j - \delta_{i,j} - s_j} \right]$$
$$\sum_{r_1=0}^{g_1-s_1} \cdots \sum_{r_i=0}^{g_i-1-s_i} \cdots \sum_{r_n=0}^{g_n-s_n} \left[\prod_{j=1}^{n} \binom{g_j - \delta_{i,j} - s_j}{r_j} z^{r_j} (1-z)^{g_j - \delta_{i,j} - s_j - r_j} \right] a_{(s_1+r_1)\cdots(s_i+r_i+1)\cdots(s_n+r_n)}, \tag{38}$$

$$\pi_{B|A}^{(G_i)} = \sum_{s_1=0}^{g_1} \cdots \sum_{s_i=0}^{g_i-1} \cdots \sum_{s_n=0}^{g_n} \left[\prod_{j=1}^{n} \binom{g_j - \delta_{i,j}}{s_j} p_A^{s_j} (1-p_A)^{g_j - \delta_{i,j} - s_j} \right]$$
$$\sum_{r_1=0}^{s_1} \cdots \sum_{r_i=0}^{s_i} \cdots \sum_{r_n=0}^{s_n} \left[\prod_{j=1}^{n} \binom{s_j}{r_j} z^{r_j} (1-z)^{s_j - r_j} \right] b_{(s_1-r_1)\cdots(s_i-r_i+1)\cdots(s_n-r_n)}. \tag{39}$$

Substituting Eqs (36-39) into Eq (30) and applying Eqs (34) and (35), we have

$$\begin{aligned} m(p_A) \approx & \frac{\omega(k-2)p_A(1-p_A)}{Nk^2} \sum_{i=1}^{n} \left[g_i q_{B|B}^{(G_i)} \left(\pi_{A|B}^{(G_i)} - \pi_{B|B}^{(G_i)} \right) + g_i q_{A|A}^{(G_i)} \left(\pi_{A|A}^{(G_i)} - \pi_{B|A}^{(G_i)} \right) \right] \\ = & \frac{\omega(k-2)p_A(1-p_A)}{Nk^2} \sum_{i=1}^{n} g_i \\ & \left\{ [zp_A + (1-p_A)] \sum_{s_1=0}^{g_1} \cdots \sum_{s_i=0}^{g_i-1} \cdots \sum_{s_n=0}^{g_n} \left[\prod_{j=1}^{n} \binom{g_j - \delta_{i,j}}{s_j} p_A^{s_j} (1-p_A)^{g_j - \delta_{i,j} - s_j} \right] c_{s_1 \cdots s_i \cdots s_n}^{(i)} \right. \\ & \left. + [p_A + z(1-p_A)] \sum_{s_1=0}^{g_1} \cdots \sum_{s_i=0}^{g_i-1} \cdots \sum_{s_n=0}^{g_n} \left[\prod_{j=1}^{n} \binom{g_j - \delta_{i,j}}{s_j} p_A^{s_j} (1-p_A)^{g_j - \delta_{i,j} - s_j} \right] d_{s_1 \cdots s_i \cdots s_n}^{(i)} \right\} \\ = & \frac{\omega(k-2)p_A(1-p_A)}{Nk^2} \sum_{s_1=0}^{g_1} \cdots \sum_{s_i=0}^{g_i} \cdots \sum_{s_n=0}^{g_n} \left[\prod_{j=1}^{n} \binom{g_j}{s_j} p_A^{s_j} (1-p_A)^{g_j - s_j} \right] e_{s_1 \cdots s_i \cdots s_n}, \end{aligned} \tag{40}$$

where

$$e_{s_1 \cdots s_i \cdots s_n} = \sum_{i=1}^{n} \left[z s_i c_{s_1 \cdots (s_i-1) \cdots s_n}^{(i)} + (g_i - s_i) c_{s_1 \cdots s_i \cdots s_n}^{(i)} + s_i d_{s_1 \cdots (s_i-1) \cdots s_n}^{(i)} + z(g_i - s_i) d_{s_1 \cdots s_i \cdots s_n}^{(i)} \right] \tag{41}$$

and

$$\begin{aligned}
&c^{(i)}_{s_1\cdots s_i\cdots s_n}\\
&=\sum_{r_1=0}^{g_1-s_1}\cdots\sum_{r_i=0}^{g_i-1-s_i}\cdots\sum_{r_n=0}^{g_n-s_n}\left[\prod_{j=1}^{n}\binom{g_j-\delta_{i,j}-s_j}{r_j}z^{r_j}(1-z)^{g_j-\delta_{i,j}-s_j-r_j}\right]a_{(s_1+r_1)\cdots(s_i+r_i)\cdots(s_n+r_n)}\\
&\quad-\sum_{r_1=0}^{s_1}\cdots\sum_{r_i=0}^{s_i}\cdots\sum_{r_n=0}^{s_n}\left[\prod_{j=1}^{n}\binom{s_j}{r_j}z^{r_j}(1-z)^{s_j-r_j}\right]b_{(s_1-r_1)\cdots(s_i-r_i)\cdots(s_n-r_n)},
\end{aligned}\tag{42}$$

$$\begin{aligned}
&d^{(i)}_{s_1\cdots s_i\cdots s_n}\\
&=\sum_{r_1=0}^{g_1-s_1}\cdots\sum_{r_i=0}^{g_i-1-s_i}\cdots\sum_{r_n=0}^{g_n-s_n}\left[\prod_{j=1}^{n}\binom{g_j-\delta_{i,j}-s_j}{r_j}z^{r_j}(1-z)^{g_j-\delta_{i,j}-s_j-r_j}\right]a_{(s_1+r_1)\cdots(s_i+r_i+1)\cdots(s_n+r_n)}\\
&\quad-\sum_{r_1=0}^{s_1}\cdots\sum_{r_i=0}^{s_i}\cdots\sum_{r_n=0}^{s_n}\left[\prod_{j=1}^{n}\binom{s_j}{r_j}z^{r_j}(1-z)^{s_j-r_j}\right]b_{(s_1-r_1)\cdots(s_i-r_i+1)\cdots(s_n-r_n)}.
\end{aligned}\tag{43}$$

Denoting

$$H(p_A)=\sum_{s_1=0}^{g_1}\cdots\sum_{s_i=0}^{g_i}\cdots\sum_{s_n=0}^{g_n}\left[\prod_{j=1}^{n}\binom{g_j}{s_j}p_A^{s_j}(1-p_A)^{g_j-s_j}\right]e_{s_1\cdots s_i\cdots s_n}\tag{44}$$

and substituting Eqs (31) and (40) into Eqs (28) and (29), for $\omega\ll 1$, we have

$$\begin{aligned}
\phi_A(x)=&\frac{\int_0^x\exp\left(-\frac{\omega N(k-1)}{k^2}\int_0^y H(r)dr\right)dy}{\int_0^1\exp\left(-\frac{\omega N(k-1)}{k^2}\int_0^y H(r)dr\right)dy}\\
=&x+\frac{\omega N(k-1)}{k^2}\left(x\int_0^1\int_0^y H(r)drdy-\int_0^x\int_0^y H(r)drdy\right)+O(\omega^2).
\end{aligned}\tag{45}$$

Based on the integral property of Bernstein polynomial (see Eq (18) in Ref [6])

$$\int_0^y\binom{g}{i}r^i(1-r)^{g-i}dr=\frac{1}{g+1}\sum_{j=i+1}^{g+1}\binom{g+1}{j}y^j(1-y)^{g+1-j},\tag{46}$$

we have

$$\begin{aligned}
\int_0^x \int_0^y H(r)drdy =& \int_0^x \int_0^y \sum_{s_1=0}^{g_1} \cdots \sum_{s_i=0}^{g_i} \cdots \sum_{s_n=0}^{g_n} \left[\prod_{j=1}^{n} \binom{g_j}{s_j} r^{s_j} (1-r)^{g_j - s_j} \right] e_{s_1 \cdots s_i \cdots s_n} drdy \\
=& \int_0^x \sum_{s_1=0}^{g_1} \cdots \sum_{s_i=0}^{g_i} \cdots \sum_{s_n=0}^{g_n} \frac{\prod_{j=1}^{n} \binom{g_j}{s_j}}{\binom{\sum_{j=1}^{n} g_j}{\sum_{j=1}^{n} s_j}} e_{s_1 \cdots s_i \cdots s_n} \\
& \int_0^y \binom{\sum_{j=1}^{n} g_j}{\sum_{j=1}^{n} s_j} r^{\sum_{j=1}^{n} s_j} (1-r)^{\sum_{j=1}^{n} g_j - \sum_{j=1}^{n} s_j} drdy \\
=& \frac{1}{\sum_{j=1}^{n} g_j + 1} \int_0^x \sum_{s_1=0}^{g_1} \cdots \sum_{s_i=0}^{g_i} \cdots \sum_{s_n=0}^{g_n} \frac{\prod_{j=1}^{n} \binom{g_j}{s_j}}{\binom{\sum_{j=1}^{n} g_j}{\sum_{j=1}^{n} s_j}} e_{s_1 \cdots s_i \cdots s_n} \\
& \sum_{l=\sum_{j=1}^{n} s_j + 1}^{\sum_{j=1}^{n} g_j + 1} \binom{\sum_{j=1}^{n} g_j + 1}{l} y^l (1-y)^{\sum_{j=1}^{n} g_j + 1 - l} dy \\
=& \frac{1}{(\sum_{j=1}^{n} g_j + 1)(\sum_{j=1}^{n} g_j + 2)} \sum_{s_1=0}^{g_1} \cdots \sum_{s_i=0}^{g_i} \cdots \sum_{s_n=0}^{g_n} \frac{\prod_{j=1}^{n} \binom{g_j}{s_j}}{\binom{\sum_{j=1}^{n} g_j}{\sum_{j=1}^{n} s_j}} e_{s_1 \cdots s_i \cdots s_n} \\
& \sum_{l=\sum_{j=1}^{n} s_j + 1}^{\sum_{j=1}^{n} g_j + 1} \sum_{m=l+1}^{\sum_{j=1}^{n} g_j + 2} \binom{\sum_{j=1}^{n} g_j + 2}{m} x^m (1-x)^{\sum_{j=1}^{n} g_j + 2 - m} \\
=& \frac{1}{(k+1)(k+2)} \sum_{m=0}^{k+2} \binom{k+2}{m} x^m (1-x)^{k+2-m} \\
& \sum_{l=0}^{m-1} \sum_{s_1=0}^{l-1} \cdots \sum_{s_i=0}^{l-1-\sum_{j=1}^{i-1} s_j} \cdots \sum_{s_n=0}^{l-1-\sum_{j=1}^{n-1} s_j} \frac{\prod_{j=1}^{n} \binom{g_j}{s_j}}{\binom{k}{\sum_{j=1}^{n} s_j}} e_{s_1 \cdots s_i \cdots s_n}
\end{aligned} \tag{47}$$

and

$$\begin{aligned}
\int_0^1 \int_0^y H(r)drdy =& \frac{1}{(k+1)(k+2)} \sum_{l=0}^{k+1} \sum_{s_1=0}^{l-1} \cdots \sum_{s_i=0}^{l-1-\sum_{j=1}^{i-1} s_j} \cdots \sum_{s_n=0}^{l-1-\sum_{j=1}^{n-1} s_j} \frac{\prod_{j=1}^{n} \binom{g_j}{s_j}}{\binom{k}{\sum_{j=1}^{n} s_j}} e_{s_1 \cdots s_i \cdots s_n} \\
=& \frac{1}{(k+1)(k+2)} \sum_{l=0}^{k} \sum_{s_1=0}^{l} \cdots \sum_{s_i=0}^{l-\sum_{j=1}^{i-1} s_j} \cdots \sum_{s_n=0}^{l-\sum_{j=1}^{n-1} s_j} \frac{\prod_{j=1}^{n} \binom{g_j}{s_j}}{\binom{k}{\sum_{j=1}^{n} s_j}} e_{s_1 \cdots s_i \cdots s_n} \\
=& \frac{1}{(k+1)(k+2)} \sum_{s_1=0}^{g_1} \cdots \sum_{s_i=0}^{g_i} \cdots \sum_{s_n=0}^{g_n} \frac{\prod_{j=1}^{n} \binom{g_j}{s_j}}{\binom{k}{\sum_{j=1}^{n} s_j}} \left(k+1-\sum_{j=1}^{n} s_j \right) e_{s_1 \cdots s_i \cdots s_n}.
\end{aligned}$$

Extending Eq (47) and taking $x = 1/N$ ($N \gg 1$), we get

$$
\begin{aligned}
&\frac{1}{(k+1)(k+2)}\left[0+0+\binom{k+2}{2}x^2(1-x)^k e_{0\cdots 0\cdots 0}+\cdots\right]\\
=&\frac{1}{(k+1)(k+2)}\binom{k+2}{2}x^2(1-x)^k e_{0\cdots 0\cdots 0}+O(x^3)\\
=&\frac{e_{0\cdots 0\cdots 0}}{2N^2}+O(\frac{1}{N^3}).
\end{aligned} \tag{48}
$$

Finally, we get the fixation probability $\rho_A = \phi_A(1/N)$ for $N \gg 1$, given by

$$
\begin{aligned}
\rho_A \approx&\frac{1}{N}+\frac{\omega N(k-1)}{k^2}\Big[\frac{1}{N(k+1)(k+2)}\sum_{s_1=0}^{g_1}\cdots\sum_{s_i=0}^{g_i}\cdots\sum_{s_n=0}^{g_n}\frac{\prod_{j=1}^{n}\binom{g_j}{s_j}}{\binom{k}{\sum_{j=1}^{n}s_j}}\left(k+1-\sum_{j=1}^{n}s_j\right)e_{s_1\cdots s_i\cdots s_n}\\
&-\frac{e_{0\cdots 0\cdots 0}}{2N^2}\Big]\\
\approx&\frac{1}{N}+\frac{\omega(k-1)}{k^2(k+1)(k+2)}\sum_{s_1=0}^{g_1}\cdots\sum_{s_i=0}^{g_i}\cdots\sum_{s_n=0}^{g_n}\frac{\prod_{j=1}^{n}\binom{g_j}{s_j}}{\binom{k}{\sum_{j=1}^{n}s_j}}\left(k+1-\sum_{j=1}^{n}s_j\right)e_{s_1\cdots s_i\cdots s_n}.
\end{aligned} \tag{49}
$$

1.6 Fixation probabilities, sigma rule and structure coefficients

Equation (49) shows that $\rho_A > \frac{1}{N}$ only if

$$
\sum_{s_1=0}^{g_1}\cdots\sum_{s_i=0}^{g_i}\cdots\sum_{s_n=0}^{g_n}\frac{\prod_{j=1}^{n}\binom{g_j}{s_j}}{\binom{k}{\sum_{j=1}^{n}s_j}}\left(k+1-\sum_{j=1}^{n}s_j\right)e_{s_1\cdots s_i\cdots s_n} > 0. \tag{50}
$$

From Eqs (41-43), $e_{s_1\cdots s_i\cdots s_n}$ is linear in $a_{s_1\cdots s_i\cdots s_n}$ and $b_{s_1\cdots s_i\cdots s_n}$. Thus the left side of formula (50) is linear in $a_{s_1\cdots s_i\cdots s_n}$ and $b_{s_1\cdots s_i\cdots s_n}$, implying that there are $\alpha_{s_1\cdots s_i\cdots s_n}$ and $\beta_{s_1\cdots s_i\cdots s_n}$ such that

$$
\begin{aligned}
&\sum_{s_1=0}^{g_1}\cdots\sum_{s_i=0}^{g_i}\cdots\sum_{s_n=0}^{g_n}\frac{\prod_{j=1}^{n}\binom{g_j}{s_j}}{\binom{k}{\sum_{j=1}^{n}s_j}}\left(k+1-\sum_{j=1}^{n}s_j\right)e_{s_1\cdots s_i\cdots s_n}\\
=&\sum_{s_1=0}^{g_1}\cdots\sum_{s_i=0}^{g_i}\cdots\sum_{s_n=0}^{g_n}\left(\alpha_{s_1\cdots s_i\cdots s_n}a_{s_1\cdots s_i\cdots s_n}+\beta_{s_1\cdots s_i\cdots s_n}b_{s_1\cdots s_i\cdots s_n}\right).
\end{aligned} \tag{51}
$$

Hence, we rewrite ρ_A and the fixation probability of a single mutant ρ_B as

$$
\begin{aligned}
\rho_A \approx&\frac{1}{N}+\frac{\omega(k-1)}{k^2(k+1)(k+2)}\\
&\sum_{s_1=0}^{g_1}\cdots\sum_{s_i=0}^{g_i}\cdots\sum_{s_n=0}^{g_n}\left(\alpha_{s_1\cdots s_i\cdots s_n}a_{s_1\cdots s_i\cdots s_n}+\beta_{s_1\cdots s_i\cdots s_n}b_{s_1\cdots s_i\cdots s_n}\right)
\end{aligned} \tag{52}
$$

and

$$\rho_B \approx \frac{1}{N} + \frac{\omega(k-1)}{k^2(k+1)(k+2)} \sum_{s_1=0}^{g_1} \cdots \sum_{s_i=0}^{g_i} \cdots \sum_{s_n=0}^{g_n} \Big(\alpha_{s_1 \cdots s_i \cdots s_n} b_{(g_1-s_1)\cdots(g_i-s_i)\cdots(g_n-s_n)} + \beta_{s_1 \cdots s_i \cdots s_n} a_{(g_1-s_1)\cdots(g_i-s_i)\cdots(g_n-s_n)} \Big). \tag{53}$$

Thus, under weak selection, we have

$$\begin{aligned}
& \rho_A > \rho_B \\
\Longleftrightarrow & \sum_{s_1=0}^{g_1} \cdots \sum_{s_i=0}^{g_i} \cdots \sum_{s_n=0}^{g_n} \left(\alpha_{s_1 \cdots s_i \cdots s_n} a_{s_1 \cdots s_i \cdots s_n} + \beta_{s_1 \cdots s_i \cdots s_n} b_{s_1 \cdots s_i \cdots s_n} \right) \\
& > \sum_{s_1=0}^{g_1} \cdots \sum_{s_i=0}^{g_i} \cdots \sum_{s_n=0}^{g_n} \left(\alpha_{s_1 \cdots s_i \cdots s_n} b_{(g_1-s_1)\cdots(g_i-s_i)\cdots(g_n-s_n)} + \beta_{s_1 \cdots s_i \cdots s_n} a_{(g_1-s_1)\cdots(g_i-s_i)\cdots(g_n-s_n)} \right) \\
\Longleftrightarrow & \sum_{s_1=0}^{g_1} \cdots \sum_{s_i=0}^{g_i} \cdots \sum_{s_n=0}^{g_n} \left(\alpha_{s_1 \cdots s_i \cdots s_n} - \beta_{(g_1-s_1)\cdots(g_i-s_i)\cdots(g_n-s_n)} \right) a_{s_1 \cdots s_i \cdots s_n} \\
& + \left(\beta_{(g_1-s_1)\cdots(g_i-s_i)\cdots(g_n-s_n)} - \alpha_{s_1 \cdots s_i \cdots s_n} \right) b_{(g_1-s_1)\cdots(g_i-s_i)\cdots(g_n-s_n)} > 0 \\
\Longleftrightarrow & \sum_{s_1=0}^{g_1} \cdots \sum_{s_i=0}^{g_i} \cdots \sum_{s_n=0}^{g_n} \left(\alpha_{s_1 \cdots s_i \cdots s_n} - \beta_{(g_1-s_1)\cdots(g_i-s_i)\cdots(g_n-s_n)} \right) \left(a_{s_1 \cdots s_i \cdots s_n} - b_{(g_1-s_1)\cdots(g_i-s_i)\cdots(g_n-s_n)} \right) > 0 \\
\Longleftrightarrow & \sum_{s_1=0}^{g_1} \cdots \sum_{s_i=0}^{g_i} \cdots \sum_{s_n=0}^{g_n} \sigma_{s_1 \cdots s_i \cdots s_n} \left(a_{s_1 \cdots s_i \cdots s_n} - b_{(g_1-s_1)\cdots(g_i-s_i)\cdots(g_n-s_n)} \right) > 0.
\end{aligned} \tag{54}$$

Equation (54) is termed "sigma rule" and its coefficients

$$\sigma_{s_1 \cdots s_i \cdots s_n} = \alpha_{s_1 \cdots s_i \cdots s_n} - \beta_{(g_1-s_1)\cdots(g_i-s_i)\cdots(g_n-s_n)} \tag{55}$$

are the structure coefficients. Here we refer to the method in Ref [4] to calculate $\alpha_{s_1 \cdots s_i \cdots s_n}$ and $\beta_{s_1 \cdots s_i \cdots s_n}$. For a multiplayer game with $a_{s_1 \cdots s_i \cdots s_n} = \prod_{i=1}^{n} \delta_{\tilde{s}_i, s_i}$ (only $a_{\tilde{s}_1 \cdots \tilde{s}_i \cdots \tilde{s}_n} = 1$ and all others are 0) and $b_{s_1 \cdots s_i \cdots s_n} = 0$, by Eq (51), we have

$$\alpha_{\tilde{s}_1 \cdots \tilde{s}_i \cdots \tilde{s}_n} = \sum_{s_1=0}^{g_1} \cdots \sum_{s_i=0}^{g_i} \cdots \sum_{s_n=0}^{g_n} \frac{\prod_{j=1}^{n} \binom{g_j}{s_j}}{\binom{k}{\sum_{j=1}^{n} s_j}} \left(k + 1 - \sum_{j=1}^{n} s_j \right) e_{s_1 \cdots s_i \cdots s_n}^{\tilde{s}_1 \cdots \tilde{s}_i \cdots \tilde{s}_n}, \tag{56}$$

where $e_{s_1 \cdots s_i \cdots s_n}^{\tilde{s}_1 \cdots \tilde{s}_i \cdots \tilde{s}_n}$ denotes the coefficient $e_{s_1 \cdots s_i \cdots s_n}$ with $a_{s_1 \cdots s_i \cdots s_n} = \prod_{i=1}^{n} \delta_{\tilde{s}_i, s_i}$ and $b_{s_1 \cdots s_i \cdots s_n} = 0$ for any combination $s_1 \cdots s_i \cdots s_n$. Analogously, $c_{s_1 \cdots s_i \cdots s_n}^{\tilde{s}_1 \cdots \tilde{s}_i \cdots \tilde{s}_n, (i)}$ and $d_{s_1 \cdots s_i \cdots s_n}^{\tilde{s}_1 \cdots \tilde{s}_i \cdots \tilde{s}_n, (i)}$ respectively correspond to

$c^{(i)}_{s_1\cdots s_i\cdots s_n}$ and $d^{(i)}_{s_1\cdots s_i\cdots s_n}$ with $a_{s_1\cdots s_i\cdots s_n} = \prod_{i=1}^{n} \delta_{\tilde{s}_i, s_i}$ and $b_{s_1\cdots s_i\cdots s_n} = 0$. From Eqs (42) and (43), we have

$$\begin{aligned} c^{\tilde{s}_1\cdots\tilde{s}_i\cdots\tilde{s}_n,(i)}_{s_1\cdots s_i\cdots s_n} =& \prod_{j=1}^{n} \binom{g_i - \delta_{i,j} - s_i}{\tilde{s}_i - s_i} z^{\tilde{s}_i - s_i} (1-z)^{g_i - \delta_{i,j} - \tilde{s}_i} \\ =& \binom{g_1 - s_1}{g_1 - \tilde{s}_1} \cdots \binom{g_i - 1 - s_i}{g_i - 1 - \tilde{s}_i} \cdots \binom{g_n - s_n}{g_n - \tilde{s}_n} \frac{(k-2)^{k-1-\sum_{j=1}^{n} \tilde{s}_j}}{(k-1)^{k-1-\sum_{j=1}^{n} s_j}} \end{aligned} \tag{57}$$

and

$$\begin{aligned} d^{\tilde{s}_1\cdots\tilde{s}_i\cdots\tilde{s}_n,(i)}_{s_1\cdots s_i\cdots s_n} =& \prod_{j=1}^{n} \binom{g_i - \delta_{i,j} - s_i}{\tilde{s}_i - \delta_{i,j} - s_i} z^{\tilde{s}_i - \delta_{i,j} - s_i} (1-z)^{g_i - \tilde{s}_i} \\ =& \binom{g_1 - s_1}{g_1 - \tilde{s}_1} \cdots \binom{g_i - 1 - s_i}{g_i - \tilde{s}_i} \cdots \binom{g_n - s_n}{g_n - \tilde{s}_n} \frac{(k-2)^{k-\sum_{j=1}^{n} \tilde{s}_j}}{(k-1)^{k-1-\sum_{j=1}^{n} s_j}}. \end{aligned} \tag{58}$$

Substituting Eqs (41,57,58) into Eq (56), we obtain

$$
\begin{aligned}
\alpha_{\tilde{s}_1\cdots\tilde{s}_i\cdots\tilde{s}_n} =& \sum_{s_1=0}^{g_1}\cdots\sum_{s_i=0}^{g_i}\cdots\sum_{s_n=0}^{g_n} \frac{\prod_{j=1}^{n}\binom{g_j}{s_j}}{\binom{k}{\sum_{j=1}^{n} s_j}} \left(k+1-\sum_{j=1}^{n} s_j\right) \\
& \sum_{i=1}^{n}\left[z s_i c^{\tilde{s}_1\cdots\tilde{s}_i\cdots\tilde{s}_n,(i)}_{s_1\cdots(s_i-1)\cdots s_n} + (g_i-s_i) c^{\tilde{s}_1\cdots\tilde{s}_i\cdots\tilde{s}_n,(i)}_{s_1\cdots s_i\cdots s_n} + s_i d^{\tilde{s}_1\cdots\tilde{s}_i\cdots\tilde{s}_n,(i)}_{s_1\cdots(s_i-1)\cdots s_n} + z(g_i-s_i) d^{\tilde{s}_1\cdots\tilde{s}_i\cdots\tilde{s}_n,(i)}_{s_1\cdots s_i\cdots s_n}\right] \\
=& \sum_{s_1=0}^{g_1}\cdots\sum_{s_i=0}^{g_i}\cdots\sum_{s_n=0}^{g_n}\sum_{i=1}^{n}\left[\frac{\prod_{j=1}^{n}\binom{g_j}{s_j+\delta_{i,j}}}{\binom{k}{\sum_{j=1}^{n}(s_j+\delta_{i,j})}}\left(k-\sum_{j=1}^{n} s_j\right)(s_i+1) z c^{\tilde{s}_1\cdots\tilde{s}_i\cdots\tilde{s}_n,(i)}_{s_1\cdots s_i\cdots s_n}\right. \\
& + \frac{\prod_{j=1}^{n}\binom{g_j}{s_j}}{\binom{k}{\sum_{j=1}^{n} s_j}}\left(k+1-\sum_{j=1}^{n} s_j\right)(g_i-s_i) c^{\tilde{s}_1\cdots\tilde{s}_i\cdots\tilde{s}_n,(i)}_{s_1\cdots s_i\cdots s_n} \\
& + \frac{\prod_{j=1}^{n}\binom{g_j}{s_j+\delta_{i,j}}}{\binom{k}{\sum_{j=1}^{n}(s_j+\delta_{i,j})}}\left(k-\sum_{j=1}^{n} s_j\right)(s_i+1) d^{\tilde{s}_1\cdots\tilde{s}_i\cdots\tilde{s}_n,(i)}_{s_1\cdots s_i\cdots s_n} \\
& \left. + \frac{\prod_{j=1}^{n}\binom{g_j}{s_j}}{\binom{k}{\sum_{j=1}^{n} s_j}}\left(k+1-\sum_{j=1}^{n} s_j\right)(g_i-s_i) z d^{\tilde{s}_1\cdots\tilde{s}_i\cdots\tilde{s}_n,(i)}_{s_1\cdots s_i\cdots s_n}\right] \\
=& \sum_{s_1=0}^{g_1}\cdots\sum_{s_i=0}^{g_i}\cdots\sum_{s_n=0}^{g_n} \frac{\prod_{j=1}^{n}\binom{g_j}{s_j}}{\binom{k}{\sum_{j=1}^{n} s_j}} \sum_{i=1}^{n}\left[\frac{g_i-s_i}{k-1}\left(k^2-(k-2)\sum_{j=1}^{n} s_j\right) c^{\tilde{s}_1\cdots\tilde{s}_i\cdots\tilde{s}_n,(i)}_{s_1\cdots s_i\cdots s_n}\right. \\
& \left. + \frac{g_i-s_i}{k-1}\left(2k+(k-2)\sum_{j=1}^{n} s_j\right) d^{\tilde{s}_1\cdots\tilde{s}_i\cdots\tilde{s}_n,(i)}_{s_1\cdots s_i\cdots s_n}\right] \qquad (59)
\end{aligned}
$$

$$
\begin{aligned}
=& \frac{(k-2)^{k-1-\sum_{j=1}^{n}\tilde{s}_j}}{k-1} \sum_{s_1=0}^{g_1}\cdots\sum_{s_i=0}^{g_i}\cdots\sum_{s_n=0}^{g_n} \frac{\prod_{j=1}^{n}\binom{g_j}{s_j}\binom{g_j-s_j}{g_j-\tilde{s}_j}}{\binom{k}{\sum_{j=1}^{n} s_j}} \\
& \left[\frac{\left(k^2-(k-2)\sum_{j=1}^{n} s_j\right)\left(k-\sum_{j=1}^{n}\tilde{s}_j\right)}{(k-1)^{k-1-\sum_{j=1}^{n} s_j}}\right. \\
& \left. + \frac{(k-2)\left(2k+(k-2)\sum_{j=1}^{n} s_j\right)\left(\sum_{j=1}^{n}\tilde{s}_j-\sum_{j=1}^{n} s_j\right)}{(k-1)^{k-1-\sum_{j=1}^{n} s_j}}\right]. \qquad (60)
\end{aligned}
$$

We make full use of a following identity to simplify Eq (60):

$$
\begin{aligned}
& \sum_{s_1=0}^{g_1}\sum_{s_2=0}^{g_2}\cdots\sum_{s_n=0}^{g_n} \frac{\prod_{j=1}^{n}\binom{g_j}{s_j}\binom{g_j-s_j}{g_j-\tilde{s}_j}}{\binom{\sum_{j=1}^{n} g_j}{\sum_{j=1}^{n} s_j}} \Theta\left(\sum_{j=1}^{n} s_j, \sum_{j=1}^{n}\tilde{s}_j\right) \\
=& \frac{\prod_{j=1}^{n}\binom{g_j}{\tilde{s}_j}}{\binom{\sum_{j=1}^{n} g_j}{\sum_{j=1}^{n}\tilde{s}_j}} \sum_{l=0}^{\sum_{j=1}^{n} g_j} \binom{\sum_{j=1}^{n} g_j - l}{\sum_{j=1}^{n} g_j - \sum_{j=1}^{n}\tilde{s}_j} \Theta\left(l, \sum_{j=1}^{n}\tilde{s}_j\right), \qquad (61)
\end{aligned}
$$

where $\Theta\left(\sum_{j=1}^n s_j, \sum_{j=1}^n \tilde{s}_j\right)$ is a function of $\sum_{j=1}^n s_j$ and $\sum_{j=1}^n \tilde{s}_j$. We here give a brief proof for this identity. First we investigate the case with $n=2$.

$$
\begin{aligned}
&\sum_{s_1=0}^{g_1}\sum_{s_2=0}^{g_2}\frac{\binom{g_1}{s_1}\binom{g_1-s_1}{g_1-\tilde{s}_1}\binom{g_2}{s_2}\binom{g_2-s_2}{g_2-\tilde{s}_2}}{\binom{g_1+g_2}{s_1+s_2}}\Theta(s_1+s_2,\tilde{s}_1+\tilde{s}_2)\\
=&\sum_{s_1=0}^{g_1}\sum_{s_2=0}^{g_2}\frac{g_1!}{s_1!(g_1-s_1)!}\frac{g_2!}{s_2!(g_2-s_2)!}\frac{(g_1-s_1)!}{(g_1-\tilde{s}_1)!(\tilde{s}_1-s_1)!}\frac{(g_2-s_2)!}{(g_2-\tilde{s}_2)!(\tilde{s}_2-s_2)!}\\
&\qquad\frac{(s_1+s_2)!(g_1+g_2-s_1-s_2)!}{(g_1+g_2)!}\Theta(s_1+s_2,\tilde{s}_1+\tilde{s}_2)\\
=&\sum_{s_1=0}^{g_1}\sum_{s_2=0}^{g_2}\frac{g_1!g_2!}{(g_1+g_2)!}\frac{(s_1+s_2)!}{s_1!s_2!}\frac{(\tilde{s}_1+\tilde{s}_2-s_1-s_2)!}{(\tilde{s}_1-s_1)!(\tilde{s}_2-s_2)!}\frac{(g_1+g_2-\tilde{s}_1-\tilde{s}_2)!}{(g_1-\tilde{s}_1)!(g_2-\tilde{s}_2)!}\\
&\qquad\frac{(g_1+g_2-s_1-s_2)!}{(g_1+g_2-\tilde{s}_1-\tilde{s}_2)!(\tilde{s}_1+\tilde{s}_2-s_1-s_2)!}\Theta(s_1+s_2,\tilde{s}_1+\tilde{s}_2)\\
=&\frac{1}{\binom{g_1+g_2}{g_1}}\sum_{s_1=0}^{g_1}\sum_{s_2=0}^{g_2}\binom{s_1+s_2}{s_1}\binom{\tilde{s}_1+\tilde{s}_2-s_1-s_2}{\tilde{s}_1-s_1}\binom{g_1+g_2-\tilde{s}_1-\tilde{s}_2}{g_1-\tilde{s}_1}\binom{g_1+g_2-s_1-s_2}{g_1+g_2-\tilde{s}_1-\tilde{s}_2}\\
&\qquad\Theta(s_1+s_2,\tilde{s}_1+\tilde{s}_2)\\
=&\frac{1}{\binom{g_1+g_2}{g_1}}\sum_{l=0}^{g_1+g_2}\sum_{s_1=0}^{l}\binom{l}{s_1}\binom{\tilde{s}_1+\tilde{s}_2-l}{\tilde{s}_1-s_1}\binom{g_1+g_2-\tilde{s}_1-\tilde{s}_2}{g_1-\tilde{s}_1}\binom{g_1+g_2-l}{g_1+g_2-\tilde{s}_1-\tilde{s}_2}\Theta(l,\tilde{s}_1+\tilde{s}_2)\\
=&\frac{1}{\binom{g_1+g_2}{g_1}}\sum_{l=0}^{g_1+g_2}\binom{\tilde{s}_1+\tilde{s}_2}{\tilde{s}_1}\binom{g_1+g_2-\tilde{s}_1-\tilde{s}_2}{g_1-\tilde{s}_1}\binom{g_1+g_2-l}{g_1+g_2-\tilde{s}_1-\tilde{s}_2}\Theta(l,\tilde{s}_1+\tilde{s}_2)\\
=&\frac{\binom{g_1}{\tilde{s}_1}\binom{g_2}{\tilde{s}_2}}{\binom{g_1+g_2}{\tilde{s}_1+\tilde{s}_2}}\sum_{l=0}^{g_1+g_2}\binom{g_1+g_2-l}{g_1+g_2-\tilde{s}_1-\tilde{s}_2}\Theta(l,\tilde{s}_1+\tilde{s}_2).
\end{aligned}
\tag{62}
$$

Then we extend the identity in case $n=2$ to any n. We decompose this long equation (see terms in

square brackets) and use Eq (62) repeatedly. Finally, we can complete the proof and obtain Eq (61).

$$
\begin{aligned}
&\sum_{s_1=0}^{g_1}\sum_{s_2=0}^{g_2}\cdots\sum_{s_n=0}^{g_n}\frac{\binom{g_1}{s_1}\binom{g_2}{s_2}\cdots\binom{g_n}{s_n}}{\binom{g_1+g_2+\cdots+g_n}{s_1+s_2+\cdots+s_n}}\binom{g_1-s_1}{g_1-\tilde{s}_1}\binom{g_2-s_2}{g_2-\tilde{s}_2}\cdots\binom{g_n-s_n}{g_n-\tilde{s}_n}\Theta\left(\sum_{j=1}^{n}s_j,\sum_{j=1}^{n}\tilde{s}_j\right)\\
=&\sum_{s_1=0}^{g_1}\sum_{s_2=0}^{g_2}\cdots\sum_{s_{n-2}=0}^{g_{n-2}}\left[\sum_{s_{n-1}=0}^{g_{n-1}}\sum_{s_n=0}^{g_n}\frac{\binom{g_{n-1}}{s_{n-1}}\binom{g_n}{s_n}}{\binom{g_{n-1}+g_n}{s_{n-1}+s_n}}\binom{g_{n-1}-s_{n-1}}{g_{n-1}-\tilde{s}_{n-1}}\binom{g_n-s_n}{g_n-\tilde{s}_n}\right]\frac{\binom{g_1}{s_1}\binom{g_2}{s_2}\cdots\binom{g_{n-2}}{s_{n-2}}}{\binom{g_1+g_2+\cdots+g_n}{s_1+s_2+\cdots+s_{n-2}+(s_{n-1}+s_n)}}\\
&\cdot\binom{g_{n-1}+g_n}{s_{n-1}+s_n}\binom{g_1-s_1}{g_1-\tilde{s}_1}\binom{g_2-s_2}{g_2-\tilde{s}_2}\cdots\binom{g_{n-2}-s_{n-2}}{g_{n-2}-\tilde{s}_{n-2}}\Theta\left(\sum_{j=0}^{n-2}s_j+(s_{n-1}+s_n),\sum_{j=1}^{n}\tilde{s}_j\right)\\
=&\sum_{s_1=0}^{g_1}\sum_{s_2=0}^{g_2}\cdots\sum_{s_{n-2}=0}^{g_{n-2}}\left[\frac{\binom{g_{n-1}}{\tilde{s}_{n-1}}\binom{g_n}{\tilde{s}_n}}{\binom{g_{n-1}+g_n}{\tilde{s}_{n-1}+\tilde{s}_n}}\sum_{l=0}^{g_{n-1}+g_n}\binom{g_{n-1}+g_n-l}{g_{n-1}+g_n-\tilde{s}_{n-1}-\tilde{s}_n}\right]\frac{\binom{g_1}{s_1}\binom{g_2}{s_2}\cdots\binom{g_{n-2}}{s_{n-2}}}{\binom{g_1+g_2+\cdots+g_n}{s_1+s_2+\cdots+s_{n-2}+l}}\\
&\cdot\binom{g_{n-1}+g_n}{l}\binom{g_1-s_1}{g_1-\tilde{s}_1}\binom{g_2-s_2}{g_2-\tilde{s}_2}\cdots\binom{g_{n-2}-s_{n-2}}{g_{n-2}-\tilde{s}_{n-2}}\Theta\left(\sum_{j=0}^{n-2}s_j+l,\sum_{j=1}^{n}\tilde{s}_j\right)\\
=&\frac{\binom{g_{n-1}}{\tilde{s}_{n-1}}\binom{g_n}{\tilde{s}_n}}{\binom{g_{n-1}+g_n}{\tilde{s}_{n-1}+\tilde{s}_n}}\sum_{s_1=0}^{g_1}\sum_{s_2=0}^{g_2}\cdots\sum_{s_{n-3}=0}^{g_{n-3}}\left[\sum_{s_{n-2}=0}^{g_{n-2}}\sum_{l=0}^{g_{n-1}+g_n}\frac{\binom{g_{n-2}}{s_{n-2}}\binom{g_{n-1}+g_n}{l}}{\binom{g_{n-2}+g_{n-1}+g_n}{l+s_{n-2}}}\binom{g_{n-1}+g_n-l}{g_{n-1}+g_n-\tilde{s}_{n-1}-\tilde{s}_n}\right.\\
&\left.\binom{g_{n-2}-s_{n-2}}{g_{n-2}-\tilde{s}_{n-2}}\right]\cdot\binom{g_{n-2}+g_{n-1}+g_n}{l+s_{n-2}}\frac{\binom{g_1}{s_1}\binom{g_2}{s_2}\cdots\binom{g_{n-3}}{s_{n-3}}}{\binom{g_1+g_2+\cdots+g_n}{s_1+s_2+\cdots+(s_{n-2}+l)}}\binom{g_1-s_1}{g_1-\tilde{s}_1}\binom{g_2-s_2}{g_2-\tilde{s}_2}\cdots\binom{g_{n-3}-s_{n-3}}{g_{n-3}-\tilde{s}_{n-3}}\\
&\cdot\Theta\left(\sum_{j=0}^{n-3}s_j+(s_{n-2}+l),\sum_{j=1}^{n}\tilde{s}_j\right)\\
=&\cdots\cdots\\
=&\frac{\prod_{j=1}^{n}\binom{g_j}{\tilde{s}_j}}{\binom{\sum_{j=1}^{n}g_j}{\sum_{j=1}^{n}\tilde{s}_j}}\sum_{l=0}^{\sum_{j=1}^{n}g_j}\binom{\sum_{j=1}^{n}g_j-l}{\sum_{j=1}^{n}g_j-\sum_{j=1}^{n}\tilde{s}_j}\Theta\left(l,\sum_{j=1}^{n}\tilde{s}_j\right).
\end{aligned}
$$

Applying Eq (61) to Eq (60) and taking $\sum_{j=1}^{n}g_j=k$, we have

$$
\begin{aligned}
\alpha_{\tilde{s}_1\tilde{s}_2\cdots\tilde{s}_n}=&\frac{(k-2)^{k-1-\sum_{j=1}^{n}\tilde{s}_j}}{k-1}\frac{\prod_{j=1}^{n}\binom{g_j}{\tilde{s}_j}}{\binom{k}{\sum_{j=1}^{n}\tilde{s}_j}}\sum_{l=0}^{k}(k-l)\left[\binom{k-1-l}{k-1-\sum_{j=1}^{n}\tilde{s}_j}\frac{k^2-(k-2)l}{(k-1)^{k-1-l}}\right.\\
&\left.+\binom{k-1-l}{k-\sum_{j=1}^{n}\tilde{s}_j}\frac{(2k+(k-2)l)(k-2)}{(k-1)^{k-1-l}}\right].
\end{aligned}
\tag{63}
$$

Analogously, we use a multi-player game with $a_{s_1\cdots s_i\cdots s_n}=0$ and $b_{s_1\cdots s_i\cdots s_n}=\prod_{i=1}^{n}\delta_{\tilde{s}_i,s_i}$ (only $b_{\tilde{s}_1\cdots\tilde{s}_i\cdots\tilde{s}_n}=1$ and all others are 0) to calculate $\beta_{\tilde{s}_1\cdots\tilde{s}_i\cdots\tilde{s}_n}$. $\beta_{\tilde{s}_1\cdots\tilde{s}_i\cdots\tilde{s}_n}$ also has the form of Eq (59).

Using Eq (51) and referring to Eqs (56-60), we have

$$
\begin{aligned}
c_{s_1\cdots s_i\cdots s_n}^{\tilde{s}_1\cdots\tilde{s}_i\cdots\tilde{s}_n,(i)} &= -\prod_{j=1}^{n}\binom{s_j}{s_j-\tilde{s}_j} z^{s_j-\tilde{s}_j}(1-z)^{\tilde{s}_j} \\
&= -\binom{s_1}{s_1-\tilde{s}_1}\cdots\binom{s_i}{s_i-\tilde{s}_i}\cdots\binom{s_n}{s_n-\tilde{s}_n}\frac{(k-2)^{\sum_{j=1}^n \tilde{s}_j}}{(k-1)^{\sum_{j=1}^n s_j}}
\end{aligned}
\tag{64}
$$

and

$$
\begin{aligned}
d_{s_1\cdots s_i\cdots s_n}^{\tilde{s}_1\cdots\tilde{s}_i\cdots\tilde{s}_n,(i)} &= -\prod_{j=1}^{n}\binom{s_j}{s_j-\tilde{s}_j+\delta_{i,j}} z^{s_j-\tilde{s}_j+\delta_{i,j}}(1-z)^{\tilde{s}_j-\delta_{i,j}} \\
&= -\binom{s_1}{s_1-\tilde{s}_1}\cdots\binom{s_i}{s_i-\tilde{s}_i+1}\cdots\binom{s_n}{s_n-\tilde{s}_n}\frac{(k-2)^{\sum_{j=1}^n \tilde{s}_j-1}}{(k-1)^{\sum_{j=1}^n s_j}}.
\end{aligned}
\tag{65}
$$

Here we give two identities, which can be derived in an analogous way to Eq (61), i.e.,

$$
\begin{aligned}
&\sum_{s_1=0}^{g_1}\sum_{s_2=0}^{g_2}\cdots\sum_{s_n=0}^{g_n}\frac{\prod_{j=1}^n\binom{g_j}{s_j}\binom{s_j}{s_j-\tilde{s}_j}}{\binom{\sum_{j=1}^n g_j}{\sum_{j=1}^n s_j}}\Theta\left(\sum_{j=1}^n s_j,\sum_{j=1}^n \tilde{s}_j\right) \\
=&\frac{\prod_{j=1}^n\binom{g_j}{\tilde{s}_j}}{\binom{\sum_{j=1}^n g_j}{\sum_{j=1}^n \tilde{s}_j}}\sum_{l=0}^{\sum_{j=1}^n g_j}\binom{l}{\sum_{j=1}^n \tilde{s}_j}\Theta\left(l,\sum_{j=1}^n \tilde{s}_j\right)
\end{aligned}
\tag{66}
$$

and

$$
\begin{aligned}
&\sum_{s_1=0}^{g_1}\sum_{s_2=0}^{g_2}\cdots\sum_{s_n=0}^{g_n}\frac{\prod_{j=1}^n\binom{g_j}{s_j}\binom{s_j}{s_j-\tilde{s}_j+\delta_{i,j}}}{\binom{\sum_{j=1}^n g_j}{\sum_{j=1}^n s_j}}(g_i-s_i)\Theta\left(\sum_{j=1}^n s_j,\sum_{j=1}^n \tilde{s}_j\right) \\
=&\frac{\tilde{s}_i}{\sum_j \tilde{s}_j}\frac{\prod_{j=1}^n\binom{g_j}{\tilde{s}_j}}{\binom{\sum_{j=1}^n g_j}{\sum_{j=1}^n \tilde{s}_j}}\sum_{l=0}^{\sum_{j=1}^n g_j}\binom{l}{\sum_{j=1}^n \tilde{s}_j-1}\Theta\left(l,\sum_{j=1}^n \tilde{s}_j\right).
\end{aligned}
\tag{67}
$$

Substituting Eqs (64) and (65) into Eq (59) and applying Eqs (66,67), we have

$$
\begin{aligned}
\beta_{\tilde{s}_1\tilde{s}_2\cdots\tilde{s}_n} =& -\frac{(k-2)^{\sum_{j=1}^n \tilde{s}_j}}{k-1}\frac{\Pi_{j=1}^n\binom{g_j}{\tilde{s}_j}}{\binom{k}{\sum_{j=1}^n \tilde{s}_j}}\sum_{l=0}^{k}(k-l)\Bigg[\binom{l}{\sum_{j=1}^n \tilde{s}_j}\frac{k^2-(k-2)l}{(k-1)^l} \\
&+\binom{l}{\sum_{j=1}^n \tilde{s}_j-1}\frac{2k+(k-2)l}{(k-2)(k-1)^l}\Bigg].
\end{aligned}
\tag{68}
$$

Substituting Eq (63) and Eq (68) into Eq (55), we have

$$
\begin{aligned}
\sigma_{s_1s_2\cdots s_n} =& \frac{(k-2)^{(k-\sum_{j=1}^n s_j)}}{k-1}\frac{\Pi_{j=1}^n\binom{g_j}{s_j}}{\binom{k}{\sum_{j=1}^n s_j}} \\
&\sum_{l=0}^{k}(k-l)\left\{\left[k^2-(k-2)l\right]\Phi\left(k,\sum_{j=1}^n s_j,l\right)+\left[2k+(k-2)l\right]\Psi\left(k,\sum_{j=1}^n s_j,l\right)\right\},
\end{aligned}
\tag{69}
$$

where

$$\Phi(k,i,l) = \binom{k-1-l}{k-1-i}\frac{1}{(k-2)(k-1)^{k-1-l}} + \binom{l}{k-i}\frac{1}{(k-1)^{l}},$$
$$\Psi(k,i,l) = \binom{k-1-l}{k-i}\frac{1}{(k-1)^{k-1-l}} + \binom{l}{k-1-i}\frac{1}{(k-2)(k-1)^{l}}.$$

Combining Eqs (52-54) and normalizing $\sigma_{s_1 s_2 \cdots s_n}$ by dividing $(k^2(k+1)(k+2))$, we have

$$\rho_A - \rho_B = \omega \sum_{s_1=0}^{g_1}\sum_{s_2=0}^{g_2}\cdots\sum_{s_n=0}^{g_n} \sigma_{s_1 s_2 \cdots s_n}\left(a_{s_1 s_2 \cdots s_n} - b_{(g_1-s_1)(g_2-s_2)\cdots(g_n-s_n)}\right), \tag{70}$$

where $\sigma_{s_1 s_2 \cdots s_n}$ is the normalized structure coefficients, given by

$$\begin{aligned}\sigma_{s_1 s_2 \cdots s_n} =& \frac{(k-2)^{(k-\sum_{j=1}^{n} s_j)}}{k^2(k+1)(k+2)} \frac{\prod_{j=1}^{n}\binom{g_j}{s_j}}{\binom{k}{\sum_{j=1}^{n} s_j}} \\ & \sum_{l=0}^{k}(k-l)\left\{\left[k^2-(k-2)l\right]\Phi\left(k,\sum_{j=1}^{n} s_j, l\right) + \left[2k+(k-2)l\right]\Psi\left(k,\sum_{j=1}^{n} s_j, l\right)\right\}.\end{aligned} \tag{71}$$

This equation corresponds to Eq (1) in the main text.

1.7 Replicator equation

Infinite populations usually serve as a baseline model to investigate the evolutionary dynamics of a system. Therefore we conduct a consistent investigation in infinite populations. The evolutionary dynamics of multiplayer games on graphs with edge diversity can be described in terms of replicator equation. Substituting Eqs (42) and (43) into Eq (40) and applying Eq (34), we have the replicator equation for evolutionary multiplayer games on graphs with n types of edges

$$\dot{x} = \frac{\omega(k-2)x(1-x)}{k^2} f(x), \tag{72}$$

where

$$f(x) = \sum_{s_1=0}^{g_1}\sum_{s_2=0}^{g_2}\cdots\sum_{s_n=0}^{g_n}\left[\prod_{j=1}^{n}\binom{g_j}{s_j} x^{s_j}(1-x)^{g_j-s_j}\right](\Lambda_a - \Lambda_b), \tag{73}$$

$$
\Lambda_a = \sum_{r_1=0}^{g_1-s_1} \sum_{r_2=0}^{g_2-s_2} \cdots \sum_{r_n=0}^{g_n-s_n} \left[\prod_{j=1}^{n} \binom{g_j - s_j}{r_j} z^{r_j} (1-z)^{g_j-s_j-r_j} \right]
$$

$$
\sum_{j=1}^{n} \left[(s_j + r_j)\, a_{(s_1+r_1)(s_2+r_2)\cdots(s_n+r_n)} + \left(z s_j + \frac{r_j}{z} \right) a_{(s_1+r_1-\delta_{1j})(s_2+r_2-\delta_{2j})\cdots(s_n+r_n-\delta_{nj})} \right],
$$

$$
\Lambda_b = \sum_{r_1=0}^{s_1} \sum_{r_2=0}^{s_2} \cdots \sum_{r_n=0}^{s_n} \left[\prod_{j=1}^{n} \binom{s_j}{r_j} z^{r_j} (1-z)^{s_j-r_j} \right]
$$

$$
\sum_{j=1}^{n} \left[(g_j - s_j + r_j)\, b_{(s_1-r_1)(s_2-r_2)\cdots(s_n-r_n)} + \left(z(g_j - s_j) + \frac{r_j}{z} \right) b_{(s_1-r_1+\delta_{1j})(s_2-r_2+\delta_{2j})\cdots(s_n-r_n+\delta_{nj})} \right].
$$

This seemingly complicated Eq (73) could be greatly simplified when applied to specific examples, such as traditional multiplayer games or pairwise games on graphs [7].

2 Section 2. Recover the previous results as a specific case with $n = 1$

We can recover previous results in Ref [4] as a specific case by taking $g_1 = k$ and $g_i = 0$ for $i \neq 1$, and thus rewrite the structure coefficient as

$$\sigma_s = \frac{(k-2)^{(k-s)}}{k^2(k+1)(k+2)} \sum_{l=0}^{k} (k-l) \left\{ \left[k^2 - (k-2)l\right] \Phi(k,s,l) + \left[2k + (k-2)l\right] \Psi(k,s,l) \right\},$$

where

$$\Phi(k,i,l) = \binom{k-1-l}{k-1-i} \frac{1}{(k-2)(k-1)^{k-1-l}} + \binom{l}{k-i} \frac{1}{(k-1)^l},$$
$$\Psi(k,i,l) = \binom{k-1-l}{k-i} \frac{1}{(k-1)^{k-1-l}} + \binom{l}{k-1-i} \frac{1}{(k-2)(k-1)^l}.$$

We can also recover the previous results by assuming that two $A-$players belonging to different types have an identical impact to their common opponent. Plainly, $a_{s_1 s_2 \cdots s_n}$ and $b_{s_1 s_2 \cdots s_n}$ are unchanged if $\sum_j s_j$ is fixed. Then the sum of $\sigma_{s_1 s_2 \cdots s_n}$ for all configurations satisfying $\sum_{j=1}^n s_j = s$ corresponds to the structure coefficient of term $a_s - b_{k-s}$. Hence, we have

$$\begin{aligned}
\sigma_s =& \sum_{\sum_{j=1}^n s_j = s} \sigma_{s_1 s_2 \cdots s_n} \\
=& \sum_{\sum_{j=1}^n s_j = s} \frac{(k-2)^{(k-1-s)}}{k^2(k+1)(k+2)} \frac{\Pi_{j=1}^n \binom{g_j}{s_j}}{\binom{k}{\sum_{j=1}^n s_j}} \\
& \sum_{l=0}^{k} (k-l) \left\{ \left[k^2 - (k-2)l\right] \Phi(k,s,l) + \left[2k + (k-2)l\right] \Psi(k,s,l) \right\} \\
=& \frac{(k-2)^{(k-1-s)}}{k^2(k+1)(k+2)} \sum_{l=0}^{k} (k-l) \left\{ \left[k^2 - (k-2)l\right] \Phi(k,s,l) + \left[2k + (k-2)l\right] \Psi(k,s,l) \right\}. \qquad (74)
\end{aligned}$$

3 Section 3. Diverse multiplayer games

The number of edges of type i is g_i. We designate m the number of different values among all g_is $(1 \le i \le n)$, l_j $(1 \le j \le m)$ the m corresponding values, and n_j the number of edge types having l_j edges. Accordingly, we have $\sum_{j=1}^{m} n_j = n$ and $\sum_{j=1}^{m} l_j n_j = k$. For a clear description, we designate $g_i = l_j$ for $i \in V_j = \left[\sum_{u=1}^{j-1} n_u + 1, \sum_{u=1}^{j} n_u\right]$. Here we investigate a scenario where each individual plays different games with different individuals simultaneously. We let individuals linked by the same type of edges form a group to play a multiplayer game. Games defined in different types of edges are independent and thus could be different in both game metaphors and payoff entries. Then the payoff can be reduced to

$$a_{s_1 s_2 \cdots s_n} = a_{s_1}^{(1)} + a_{s_2}^{(2)} + \cdots + a_{s_n}^{(n)}, \tag{75}$$

$$b_{s_1 s_2 \cdots s_n} = b_{s_1}^{(1)} + b_{s_2}^{(2)} + \cdots + b_{s_n}^{(n)}. \tag{76}$$

$a_{s_i}^{(i)}$ $(b_{s_i}^{(i)})$ represents the payoff of an $A-$player (a $B-$player) obtained from the interaction with individuals connected by edges of type i where there are s_i opposing A-players.

3.1 Finite populations

We first investigate how the independence of payoffs obtained in different games affects the “sigma rule” [see Eq (54)]. Using the above notations, we have

$$\begin{aligned}
&\sum_{s_1=0}^{g_1} \sum_{s_2=0}^{g_2} \cdots \sum_{s_n=0}^{g_n} \sigma_{s_1 s_2 \cdots s_n} \left(a_{s_1 s_2 \cdots s_n} - b_{(g_1-s_1)(g_2-s_2)\cdots(g_n-s_n)}\right) > 0 \\
\Longleftrightarrow &\sum_{s_1=0}^{g_1} \sum_{s_2=0}^{g_2} \cdots \sum_{s_n=0}^{g_n} \sigma_{s_1 s_2 \cdots s_n} \sum_{i=1}^{n} (a_{s_i}^{(i)} - b_{g_i-s_i}^{(i)}) > 0 \\
\Longleftrightarrow &\sum_{s_1=0}^{g_1} \sum_{s_2=0}^{g_2} \cdots \sum_{s_n=0}^{g_n} \sigma_{s_1 s_2 \cdots s_n} \sum_{j=1}^{m} \sum_{i \in V_j} (a_{s_i}^{(i)} - b_{g_i-s_i}^{(i)}) > 0 \\
\Longleftrightarrow &\sum_{j=1}^{m} \sum_{i \in V_j} \sum_{s_1=0}^{g_1} \sum_{s_2=0}^{g_2} \cdots \sum_{s_n=0}^{g_n} \sigma_{s_1 s_2 \cdots s_n} (a_{s_i}^{(i)} - b_{g_i-s_i}^{(i)}) > 0.
\end{aligned} \tag{77}$$

Here we analyze the case for $j = 1$, $V_1 = [1, n_1]$, and $g_1 = g_2 = \cdots = g_{n_1} = l_1$. Other cases can be calculated analogously. We have

$$\begin{aligned}
&\sum_{i\in V_1}\sum_{s_1=0}^{g_1}\sum_{s_2=0}^{g_2}\cdots\sum_{s_n=0}^{g_n}\sigma_{s_1s_2\cdots s_n}(a_{s_i}^{(i)} - b_{g_i-s_i}^{(i)}) \\
=&\sum_{s_1=0}^{g_1}(a_{s_1}^{(1)} - b_{g_1-s_1}^{(1)})\sum_{s_2=0}^{g_2}\sum_{s_3=0}^{g_3}\cdots\sum_{s_n=0}^{g_n}\sigma_{s_1s_2\cdots s_n} + \sum_{s_2=0}^{g_2}(a_{s_2}^{(2)} - b_{g_2-s_2}^{(2)})\sum_{s_1=0}^{g_1}\sum_{s_3=0}^{g_3}\cdots\sum_{s_n=0}^{g_n}\sigma_{s_1s_2\cdots s_n} + \cdots \\
&+ \sum_{s_{n_1}=0}^{g_{n_1}}(a_{s_{n_1}}^{(n_1)} - b_{g_{n_1}-s_{n_1}}^{(n_1)})\sum_{s_1=0}^{g_1}\cdots\sum_{s_{n_1-1}=0}^{g_{n_1-1}}\sum_{s_{n_1+1}=0}^{g_{n_1+1}}\cdots\sum_{s_n=0}^{g_n}\sigma_{s_1s_2\cdots s_n}. \qquad (78)
\end{aligned}$$

From Eq (74), we have

$$\sigma_{s_1s_2\cdots s_n} = \frac{\binom{k}{\sum_{j=1}^n s_j}}{\Pi_{j=1}^n\binom{g_j}{s_j}}\sigma_{\sum_{j=1}^n s_j}. \qquad (79)$$

Especially, for $g_i = g_j$, $\sigma_{s_1s_2\cdots s_n}$ remains unchanged after exchanging the i_{th} and the j_{th} subscripts of $\sigma_{s_1s_2\cdots s_n}$, i.e., $\sigma_{s_1\cdots s_{i-1}s_is_{i+1}\cdots s_{j-1}s_js_{j+1}\cdots s_n} = \sigma_{s_1\cdots s_{i-1}s_js_{i+1}\cdots s_{j-1}s_is_{j+1}\cdots s_n}$. Denoting

$$\sum_{s_2=0}^{g_2}\sum_{s_3=0}^{g_3}\cdots\sum_{s_n=0}^{g_n}\sigma_{s_1s_2\cdots s_n} = \tilde{\sigma}_{s_1}^{(1)}, \qquad (80)$$

we have

$$\sum_{s_1=0}^{g_1}\sum_{s_3=0}^{g_3}\cdots\sum_{s_n=0}^{g_n}\sigma_{s_1s_2\cdots s_n} = \sum_{s_1=0}^{g_1}\sum_{s_3=0}^{g_3}\cdots\sum_{s_n=0}^{g_n}\sigma_{s_2s_1\cdots s_n} = \tilde{\sigma}_{s_2}^{(1)} \qquad (81)$$

and

$$\sum_{s_1=0}^{g_1}\cdots\sum_{s_{n_1-1}=0}^{g_{n_1-1}}\sum_{s_{n_1+1}=0}^{g_{n_1+1}}\cdots\sum_{s_n=0}^{g_n}\sigma_{s_1s_2\cdots s_n} = \tilde{\sigma}_{s_{n_1}}^{(1)}. \qquad (82)$$

Overall, Eq (78) can be rewritten as

$$\begin{aligned}
&\sum_{s_1=0}^{g_1}(a_{s_1}^{(1)} - b_{g_1-s_1}^{(1)})\tilde{\sigma}_{s_1}^{(1)} + \sum_{s_2=0}^{g_2}(a_{s_2}^{(2)} - b_{g_2-s_2}^{(2)})\tilde{\sigma}_{s_2}^{(1)} + \cdots + \sum_{s_{n_1}=0}^{g_{n_1}}(a_{s_{n_1}}^{(n_1)} - b_{g_{n_1}-s_{n_1}}^{(n_1)})\tilde{\sigma}_{s_{n_1}}^{(1)} \\
=&\sum_{s=0}^{l_1}\tilde{\sigma}_s^{(1)}\left(\sum_{i\in V_1}a_s^{(i)} - \sum_{i\in V_1}b_{l_1-s}^{(i)}\right). \qquad (83)
\end{aligned}$$

Substituting Eq (83) into Eq (77), we have

$$\sum_{j=1}^{m}\sum_{s=0}^{l_j}\tilde{\sigma}_s^{(j)}\left(\sum_{i\in V_j}a_s^{(i)} - \sum_{i\in V_j}b_{l_j-s}^{(i)}\right) > 0, \qquad (84)$$

where

$$\tilde{\sigma}_s^{(j)} = \sum_{s_1=0}^{g_1} \cdots \sum_{s_{r-1}=0}^{g_{r-1}} \sum_{s_{r+1}=0}^{g_{r+1}} \cdots \sum_{s_n=0}^{g_n} \sigma_{s_1 \cdots s_{r-1} s s_{r+1} \cdots s_n} \tag{85}$$

and $r = \sum_{u=1}^{j-1} n_u + 1$. Thus the effects of the population structure are captured by $\sum_{j=1}^{m}(l_j + 1)$ structure coefficients. Especially, for $g_1 = g_2 = \cdots = g_n = g$, $\rho_A > \rho_B$ is equivalent to

$$\sum_{s=0}^{g} \tilde{\sigma}_s^{(1)} \left(\sum_{i=1}^{n} a_s^{(i)} - \sum_{i=1}^{n} b_{g-s}^{(i)} \right) > 0. \tag{86}$$

Replacing $\tilde{\sigma}_s^{(1)}$ with $\tilde{\sigma}_s$ in Eq (86), we get Eq (4) in the main text. Using Eqs (52), (53), (63) and (68), we find that for sufficient large populations the fixation probabilities (both ρ_A and ρ_B) under diverse multiplayer games can be approximated by assuming players playing a unified game, where the payoff structure correspond to the average over all games.

3.2 Infinite populations

We proceed with the study of diverse multiplayer games in infinite populations. Applying Eqs (75) and (76) into Eq (73), we have

$$\begin{aligned}
f(x) =& \sum_{j=1}^{n} \sum_{s_j=0}^{g_j} \binom{g_j}{s_j} x^{s_j} (1-x)^{g_j - s_j} \sum_{r_j=0}^{g_j - s_j} \binom{g_j - s_j}{r_j} z^{r_j} (1-z)^{g_j - s_j - r_j} \\
& \left[\left[(1+z)(k - g_j) + s_j + r_j \right] a_{s_j + r_j}^{(j)} + \left(z s_j + \frac{r_j}{z} \right) a_{s_j + r_j - 1}^{(j)} \right] \\
& - \sum_{j=1}^{n} \sum_{s_j=0}^{g_j} \binom{g_j}{s_j} x^{s_j} (1-x)^{g_j - s_j} \sum_{r_j=0}^{s_j} \binom{s_j}{r_j} z^{r_j} (1-z)^{s_j - r_j} \\
& \left[\left[(1+z)(k - g_j) + g_j - s_j + r_j \right] b_{s_j - r_j}^{(j)} + \left(z(g_j - s_j) + \frac{r_j}{z} \right) b_{s_j - r_j + 1}^{(j)} \right] \\
=& \sum_{i=1}^{m} \sum_{s=0}^{l_i} \binom{l_i}{s} x^s (1-x)^{l_i - s} \sum_{r=0}^{l_i - s} \binom{l_i - s}{r} z^r (1-z)^{l_i - s - r} \\
& \left[\left[(1+z)(k - l_i) + s + r \right] \sum_{j \in V_i} a_{s+r}^{(j)} + \left(z s + \frac{r}{z} \right) \sum_{j \in V_i} a_{s+r-1}^{(j)} \right] \\
& - \sum_{i=1}^{m} \sum_{s=0}^{l_i} \binom{l_i}{s} x^s (1-x)^{l_i - s} \sum_{r=0}^{s} \binom{s}{r} z^r (1-z)^{s-r} \\
& \left[\left[(1+z)(k - l_i) + l_i - s + r \right] \sum_{j \in V_i} b_{s-r}^{(j)} + \left(z(l_i - s) + \frac{r}{z} \right) \sum_{j \in V_i} b_{s-r+1}^{(j)} \right].
\end{aligned} \tag{87}$$

Furthermore, if we introduce two notations

$$\bar{a}_s^{(i)} = \frac{1}{n_i} \sum_{j \in V_i} a_s^{(j)},$$
$$\bar{b}_s^{(i)} = \frac{1}{n_i} \sum_{j \in V_i} b_s^{(j)},$$

which correspond to the average of payoff values over games of same sizes, i.e., g_js are identical for $j \in V_i$. In other words, for games of same sizes, we can use the average of their payoff values to approximate the evolutionary dynamics. Especially, for $g_1 = g_2 = \cdots = g_n = g$, the evolutionary dynamics can be approximated by a unified payoff structure

$$\bar{a}_s = \frac{1}{n} \sum_{j=1}^{n} a_s^{(j)},$$
$$\bar{b}_s = \frac{1}{n} \sum_{j=1}^{n} b_s^{(j)}.$$

We end this section by an example of evolutionary games on weighted networks with $g_1 = g_2 = \cdots = g_n = g$. We endow the j_{th} type of edges a weight ζ_j. The payoff structure is

$$a_s^{(j)} = \zeta_j a_s, \tag{88}$$
$$b_s^{(j)} = \zeta_j b_s, \tag{89}$$

where a_s (b_s) is a function of s. Substituting Eqs (88) and (89) into Eq (87), we have that

$$\begin{aligned} f(x) = & \sum_{j=1}^{n} \zeta_j \cdot \sum_{s=0}^{g} \binom{g}{s} x^s (1-x)^{g-s} \\ & \left\{ \sum_{r=0}^{g-s} \binom{g-s}{r} z^r (1-z)^{g-s-r} \left[\left[(1+z)(k-g) + s + r \right] a_{s+r} + \left(zs + \frac{r}{z} \right) a_{s+r-1} \right] \right. \\ & \left. - \sum_{r=0}^{s} \binom{s}{r} z^r (1-z)^{s-r} \left[\left[(1+z)(k-g) + g - s + r \right] b_{s-r} + \left(z(g-s) + \frac{r}{z} \right) b_{s-r+1} \right] \right\}. \end{aligned} \tag{90}$$

Equation (90) shows that the values of x satisfying $f(x) = 0$ are independent of ζ_j for any j. Thus, nonuniform strength of interactions does not affect the evolutionary dynamics.

4 Section 4. Sigma rule and structure coefficient for evolutionary two-player games on graphs with n types of edges

In evolutionary two-player games on graphs, interactions occurring in each type of edges are assigned a payoff matrix. The payoff matrix for interactions occurring in edges of type i is

$$\begin{array}{c} \\ \text{A} \\ \text{B} \end{array}\begin{array}{c} \begin{array}{cc} \text{A} & \text{B} \end{array} \\ \begin{pmatrix} \alpha_i & \beta_i \\ \gamma_i & \theta_i \end{pmatrix} \end{array},$$

where each value corresponds to the payoff assigned to the individual adopting a strategy in the row against its partner taking a strategy in the column. Transforming the payoff to multiplayer interactions through $a_{s_1 s_2 \cdots s_n} = \sum_{i=1}^{n} [s_i \alpha_i + (g_i - s_i)\beta_i]$ and $b_{s_1 s_2 \cdots s_n} = \sum_{i=1}^{n} [s_i \gamma_i + (g_i - s_i)\theta_i]$, we have the sigma rule from Eq (55)

$$\sum_{i=1}^{n} \bar{s}_i \alpha_i + \sum_{i=1}^{n} (g_i - \bar{s}_i) \beta_i - \sum_{i=1}^{n} (g_i - \bar{s}_i) \gamma_i - \sum_{i=1}^{n} \bar{s}_i \theta_i > 0, \tag{91}$$

where

$$\bar{s}_i = \sum_{s_1=0}^{g_1} \sum_{s_2=0}^{g_2} \cdots \sum_{s_n=0}^{g_n} \sigma_{s_1 s_2 \cdots s_n} s_i.$$

Here we show how to get $\bar{s}_i$ relying on a previous study [8]. Assuming that interactions along all edges except edges of type i bring no benefits ($\alpha_j = 0, \beta_j = 0, \gamma_j = 0, \theta_j = 0$ for $j \neq i$), Eq (91) can be rewritten as

$$\rho_A > \rho_B \Longleftrightarrow \bar{s}_i \alpha_i + (g_i - \bar{s}_i) \beta_i - (g_i - \bar{s}_i) \gamma_i - \bar{s}_i \theta_i > 0. \tag{92}$$

From the perspective of separated interaction graph and replacement graph, in the interaction graph, these edges corresponding to interactions with no payoffs seem to be removed, leading to asymmetric interaction and replacement graphs. From Ref [8], we have

$$\rho_A > \rho_B \Longleftrightarrow (k+1)\alpha_i + (k-1)\beta_i - (k-1)\gamma_i - (k+1)\theta_i > 0. \tag{93}$$

Comparing Eqs (92) and (93), we have

$$\bar{s}_i = \frac{g_i(k+1)}{2k}. \tag{94}$$

Thus we have sigma rule shown in the main text

$$\sum_{i=1}^{n} \left[g_i(k+1)\alpha_i + g_i(k-1)\beta_i \right] > \sum_{i=1}^{n} \left[g_i(k-1)\gamma_i + g_i(k+1)\theta_i \right].$$

5 Section 5. Simulation data in a real-world friendship network

Here we ran simulations in a real-world friendship network [9] and we show that our theoretical results qualitatively predict the evolutionary outcomes.

This friendship network is built from an in-school friendship nomination questionnaire implemented in 1994/1995. In this survey, each student listed his/her 5 best female and 5 best male friends. Meanwhile, the interaction frequency between any two students was also recorded, which varies from 1 to 6. In the network, each node represents a student and a directed edge from node i to j means that i chose j as his/her best friend. A larger edge weight indicates more interactions. There are in total 2539 nodes and 12969 edges. In the current study, for simplicity, we use this network in such ways:

(1) treat the network as undirected and assign each edge with a weight corresponding to the average of interaction frequencies from node i to j and that from node j to i;

(2) set a threshold and classify all edges into two categories: edges with a weight above the threshold are of type 1 and the rest are of type 2.

Taking a threshold of 1.5, we then have a friendship network with two types of edges. On average, a node is linked to 4.3 neighbors by edges of type 1 and to 4 neighbors by edges of type 2. We consider a representative example where each individual plays a volunteer's game with neighbors linked by edges of type 1 and plays a public goods game with neighbors linked by edges of type 2. Other examples can be analyzed analogously.

Figure S3 shows the average change (Δp_A) in the frequency of A-players (p_A) in this real-world network. Our analytical results qualitatively predict the evolutionary direction, such as the dominance of defection (Fig. S3a), the coexistence of defection and cooperation (Fig. S3b) and the dominance of cooperation (Fig. S3c). Within expectation, there are deviations between the simulation data and analytical results. The reason lies in the following:

(1) the pair approximation method we employed, in theory, applies only to large random regular graphs, where each node has exact k neighbors and there are no loops. However, this real-world social network is degree-heterogeneous: a few nodes have 27 neighboring nodes while some other

nodes have only 1 neighboring node. Besides, the clustering coefficient of this network is 0.142, which means that there are a lot of short loops.

(2) In our theoretical analysis, the node degree is an integer. But in the practical social network, a node is connected to 4.3 neighboring nodes by edges of type 1 on average. We choose the approximate value of 4 in the theoretical calculation, which also causes the deviation.

6 Section 6. Computer simulations

Network generation: We present the procedure to produce a random regular graph with n types of edges, where the number of edges of type i linked to each node is g_i $(1 \leq i \leq n)$. We take $g_1 \geq 2$. Given values of g_i, we first construct a random regular graph of degree g_1 and make sure that it is connected. All edges in this graph are assigned to be type 1. Then we augment this graph by increasing the degree of all nodes by g_2. All edges added in this step are assigned to be type 2. Repeating this procedure for $n-1$ times where the augment degree is g_{i+1} in the i_{th} augment, we assign the edges added in the i_{th} augment to be type $i+1$. Finally, we generate a random regular graph with degree $\sum_{i=1}^{n} g_i$.

Fixation probability ρ_{A}: In a generated random regular graph with $N = 200$ and $n = 2$ (g_1 and g_2 are given in corresponding figures), a random node is selected to be A-player and the rest are B-players. The system evolves as described in Models with selection intensity $\omega = 0.01$. The evolution does not end until all nodes turn to A-players or B-players. Repeating graph generation and subsequent system evolution for 10^7 runs, ρ_{A} is the fraction of times where A-players reach fixations. ρ_{B} is calculated analogously.

Replicator equation: In a generated random regular graph with $N = 1000$ and $g_1 = 3$, $g_2 = 3$, a random value of f is sampled uniformly from the interval $[0, 1]$. Then each node is initiated to be a cooperator with probability f and a defector otherwise. The system evolves as described in Models with selection intensity $\omega = 0.01$. We term a time step during which the population updates N times. Let $p_{\text{A}}(t)$ denote the frequency of A-players at time step t and $p_{\text{A}}(0)$ the initial frequency of A-players. Let $\Delta p_{\text{A}}(t)$ denote the change in frequency of A-players within a time step starting at time step t, i.e., $\Delta p_{\text{A}}(t) = p_{\text{A}}(t+1) - p_{\text{A}}(t)$. $\Delta p_{\text{A}}(t)$ is associated with $p_{\text{A}}(t)$ and is recorded. Each simulation runs 100000 time steps. The graph generation, sample of f, and subsequent system evolution, are repeated for 50000 times (50000 simulations) if there is an inner equilibria, which can be predicted by Eq (72), and for 1000000 times if there is no any inner equilibria. Finally, Δp_{A} corresponding to p_{A} is the average of recorded $\Delta p_{\text{A}}(t)$, as plotted in Figs 2 and 3 in the main text. We also ran simulations in a real-world friendship network (refer to Ref. [9]) and we show that our theoretical results qualitatively predict the evolutionary outcomes (see S3 Fig).

References

1. Ohtsuki H, Hauert C, Lieberman E, Nowak MA. A simple rule for the evolution of cooperation on graphs and social networks. Nature. 2006;441:502–505. doi:10.1038/nature04605.

2. Khalil HK. Nonlinear Systems. Prentice Hall; 2001.

3. Gardiner CW. Handbook of Stochastic Methods. Springer; 2004.

4. Peña J, Wu B, Arranz J, Traulsen A. Evolutionary games of multiplayer cooperation on graphs. PLoS Computational Biology. 2016;12(8):e1005059. doi:10.1371/journal.pcbi.1005059.

5. Peña J, Nöldeke G, Lehmann L. Evolutionary dynamics of collective action in spatially structured populations. Journal of Theoretical Biology. 2015;382:122–136. doi:10.1016/j.jtbi.2015.06.039.

6. Farouki RT. The Bernstein polynomial basis: A centennial retrospective. Computer Aided Geometric Design. 2012;29(6):379–419. doi:10.1016/j.cagd.2012.03.001.

7. Ohtsuki H, Nowak MA. The replicator equation on graphs. Journal of Theoretical Biology. 2006;243(1):86–97. doi:10.1016/j.jtbi.2006.06.004.

8. Ohtsuki H, Nowak MA, Pacheco JM. Breaking the symmetry between interaction and replacement in evolutionary dynamics on graphs. Physical Review Letters. 2007;98:108106. doi:10.1103/PhysRevLett.98.108106.

9. Moody, J. Peer influence groups: identifying dense clusters in large networks. Social Networks. 2001;23(4):261–283. doi:10.1016/S0378-8733(01)00042-9.
